# From Concepts to Inhibitors: A Blueprint for Targeting Protein–Protein Interactions

**DOI:** 10.1021/acs.chemrev.5c00046

**Published:** 2025-06-24

**Authors:** Seong Ho Hong, Thu Nguyen, Joseph F. Ongkingco, Alex Nazzaro, Paramjit S. Arora

**Affiliations:** Department of Chemistry, 5894 New York University, New York, New York 10003, United States

## Abstract

Protein–protein interactions are no longer considered undruggable because of the conceptual and technical advances that allow inhibitors to be generated using rational design principles and high-throughput screening methods. Here we review the concepts and approaches that have underpinned the progress in this field. We begin by assessing what makes a protein surface more tractable than others with a focus on the recent success in targeting Ras, which has long served as a poster child of a therapeutically important yet undruggable target. We discuss computational approaches to dissect protein surfaces to design macrocycles and miniprotein ligands. Traditional drug discovery has benefitted from leveraging natural products but this benefit has not extended to the design of ligands for protein surfaces because few natural products have been characterized as inhibitors of protein complexes. However, nature does provide a template in the form of binding epitopes of partner proteins. We review design of protein structure mimics that enable rational design of inhibitors through multiple weak contacts. Lastly, we focus on contemporary screening methods that are being merged with constrained peptides to offer unprecedented side chain diversity on conformationally defined scaffolds. We will focus on the concepts underlying advancements in the field rather than the application of these concepts and technologies that have led to inhibitors of specific interactions.

## Graphical abstract

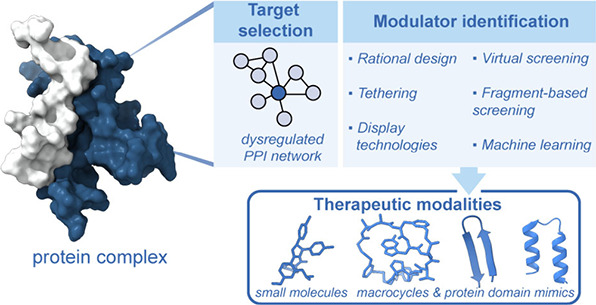

## 1 Introduction

In 1987, Nobel laureate Arthur Kornberg articulated a poignant observation: “We now have the paradox of the two cultures, chemistry and biology, growing farther apart even as they discover more common ground.”^1^ Over the past three decades, there has been a growing integration of chemistry and biology, contrary to Kornberg’s apprehension, with collaborative efforts leading to significant biomedical advances. The field of chemical biology, built on this convergence of chemistry and biology, has offered new avenues to probe and target biomolecular interactions with synthetic ligands. Chemoproteomics reagents and bioorthogonal chemistry are now routinely employed in biological groups to decipher the complexity of interaction networks. The revolution in Omics sciences has revealed a wealth of targets that can be interrogated for next-generation therapeutics. This review focuses on the development of synthetic ligands that can modulate protein–protein interaction (PPI) networks to build on the success of the omics revolution. PPIs have been termed *undruggable*,^2^ but advances in structural biology and computational chemistry, together with innovations in chemical design and synthesis have shepherded an exciting era in drug discovery. Herein, we aim to provide a roadmap for newcomers to the field interested in developing ligands for protein surfaces (Figure 1).

Interactions of proteins with binding partners governs a range of processes from signal transduction, gene expression, and cell cycle. Dysregulation of these fundamental mechanisms leads to disease; thus, PPIs represent attractive targets for therapeutic intervention. However, compared to traditional drug targets,^3^ such as enzymes, GPCRs, or nuclear receptors, PPIs have proven to be more difficult to drug. Two factors that raise the difficulty in targeting PPIs over enzymes and GPCRs include (i) Enzymes and GPCRs often possesses sculpted binding sites naturally suited for small molecules and (ii) Nature has offered scaffolds that guide drug discovery efforts, especially for GPCRs and Enzymes;^4,5^ in contrast, proteins often use large, flat interfaces to engage other proteins (Figure 2A–C), and classes of natural products that engage PPIs are not well identified. These two factors represent critical challenges in the development of specific ligands for interacting protein surfaces. In addition, the transient and context-dependent nature of PPIs (Figure 2D), coupled with the lack of structural information and understanding of the PPI interactome, further complicates discovery of PPI modulators.^6,7^ Addressing these challenges has required advances in rational design and screening approaches. Successful examples of small molecule modulators of PPIs have been limited, though a few have progressed to clinical trials.

A paradigm shifting PPI modulator was demonstrated by scientists at Abbvie to target antiapopototic protein B-cell lymphoma 2 (BCL2). BCL2 binds the BH3 domain of pro-apoptotic Bax via an α-helical sequence (Figure 3A). A BH3 mimetic, Venetoclax, was discovered by a fragment-based drug discovery (FBDD) campaign and functions by inhibiting BCL-2 mediated PPIs, which restores the apoptotic pathway in cancer cells and leads to death. No longer just a clinical candidate, Venetoclax has demonstrated high efficacy in leukemia patients, particularly those with chronic lymphocytic leukemia.^8^

KRas represents a recent example of a protein that was previously termed undruggable but has now yielded to drug design (Figure 3B).^9–13^ The critical role of KRas as an oncogenic protein has been known for several decades,^14^ but a lack of a defined binding site has stifled inhibitor design. An innovative fragment screening approach, termed protein tethering (*vide infra*),^15,16^ revealed a covalent small molecule that engages mutant G12C KRas.^17,18^ Leads developed from this approach have demonstrated effective inhibition of KRas activation (GTP bound form) by irreversibly locking the protein in its inactive, GDP-bound conformation. This mechanism disrupts downstream signaling pathway, ultimately inhibiting cancer cell proliferation.^13^ Sotorasib has transitioned from a clinical candidate to an approved therapy, demonstrating efficacy in treating patients with KRas G12C mutated cancers, particularly nonsmall cell lung cancer (NSCLC).

This review aims to serve as a guide for newcomers in the field of chemistry, particularly those interested in designing inhibitors for *intracellular* PPIs. In the following sections, we will discuss methods for identifying PPI that may be amenable to synthetic inhibitors. Using KRas as an example, we will discuss the attributes that make certain protein targets and their interfaces promising for therapeutic intervention. Computational experimental approaches to choose potential modalities as inhibitors for different protein interfaces are highlighted. Proteins often employ folded domains to recognize binding partners and mimicry of these folded regions has led to rational design approaches to inhibit PPIs. We discuss the role of protein mimics as synthetic epitopes for inhibitor design. Finally, we describe screening approaches to identify and optimize inhibitors, with an emphasis on how these screening methods are merging with protein mimicry strategies. These diverse yet complementary approaches offer the necessary tools that underpin ongoing efforts in academia and industry for targeting undruggable space of PPIs.

**Figure 1:**
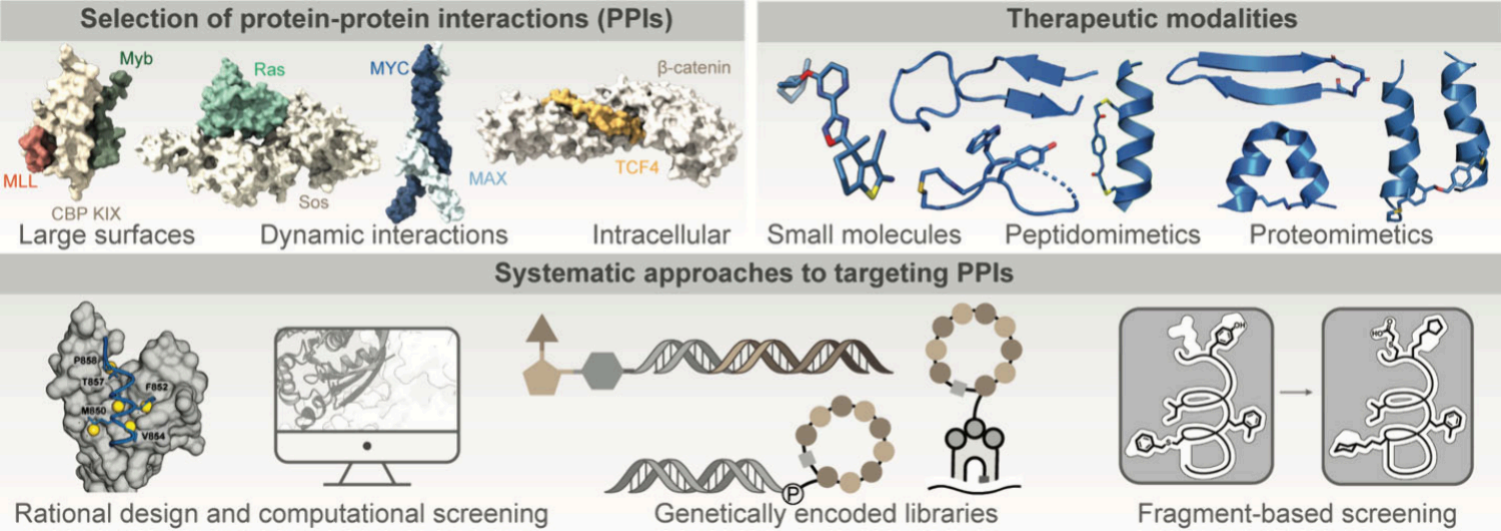
Blueprint for targeting protein–protein interactions. In this review, we discuss computational and experimental approaches to identify targetable protein interfaces and rational design and screening approaches to develop small molecule and peptide modulators of PPIs.

**Figure 2:**
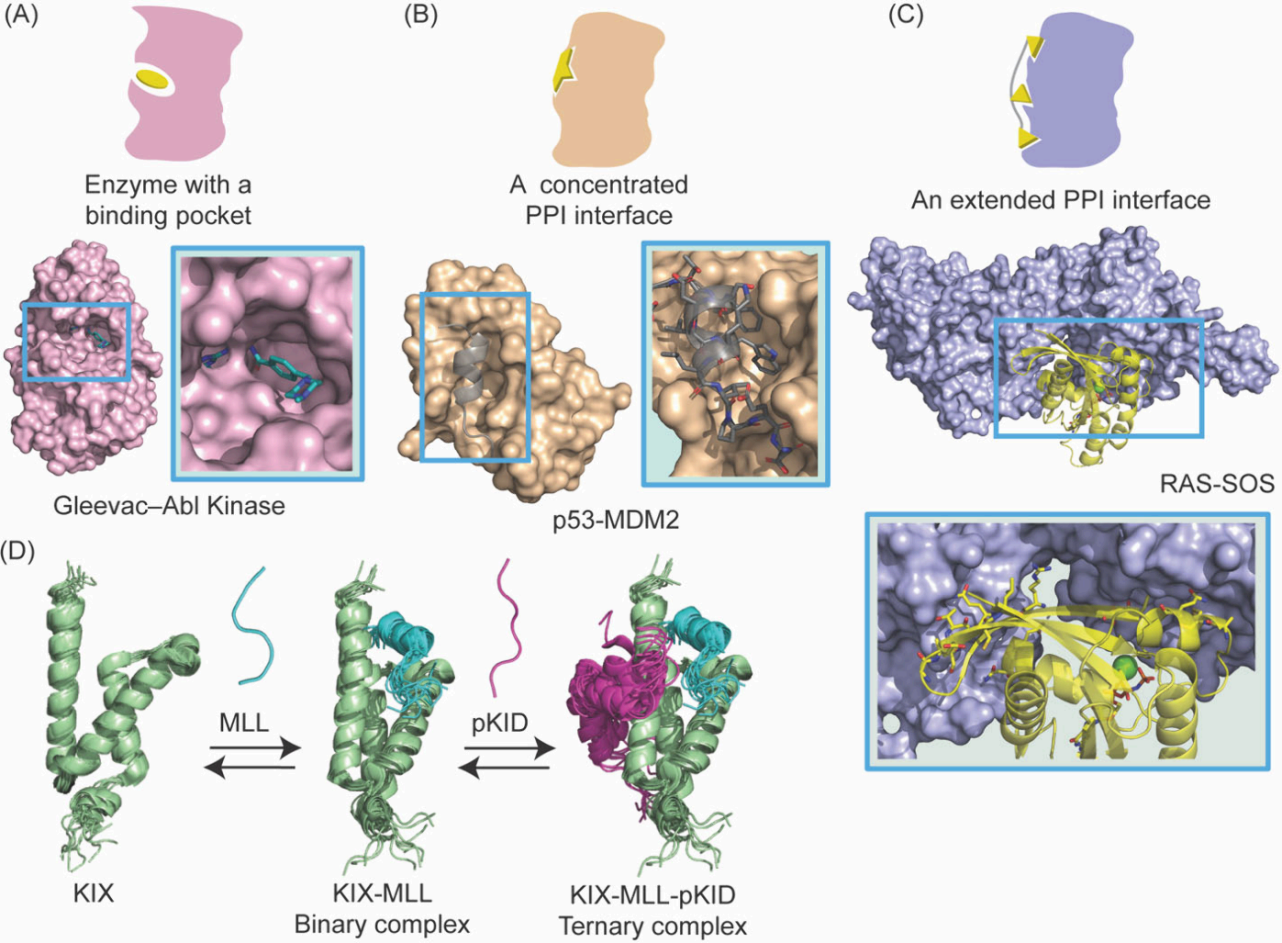
Two critical challenges in development of PPI inhibitors are that the binding interfaces are larger than those accommodated by small molecules and the protein complexes are often dynamic. (A) Traditional protein drug targets, such as GPCRs and enzymes, often feature deep hydrophobic binding pockets as illustrated by the binding of Gleevec to Abl kinase (left, PDB: 1XBB). (B, C) The large interfaces typical of PPIs are illustrated by binding of Ras to its regulator Sos (bottom, PDB: 1BKD). There are examples of PPIs where a single secondary structure provides the critical binding epitope for complex formation. In such cases, as illustrated by the complex of tumor suppressor protein p53 with E3-ligase MDM2 (right, PDB: 1YCR), the binding interfaces can be reminiscent of conventional drug pockets. (D) The ternary complex of coactivator KIX to transcription factors MLL and pKID highlights the dynamic nature of PPIs (PDB: 2LXT). Conformational dynamics lead to changes in binding sites to complicate inhibitor design.

**Figure 3:**
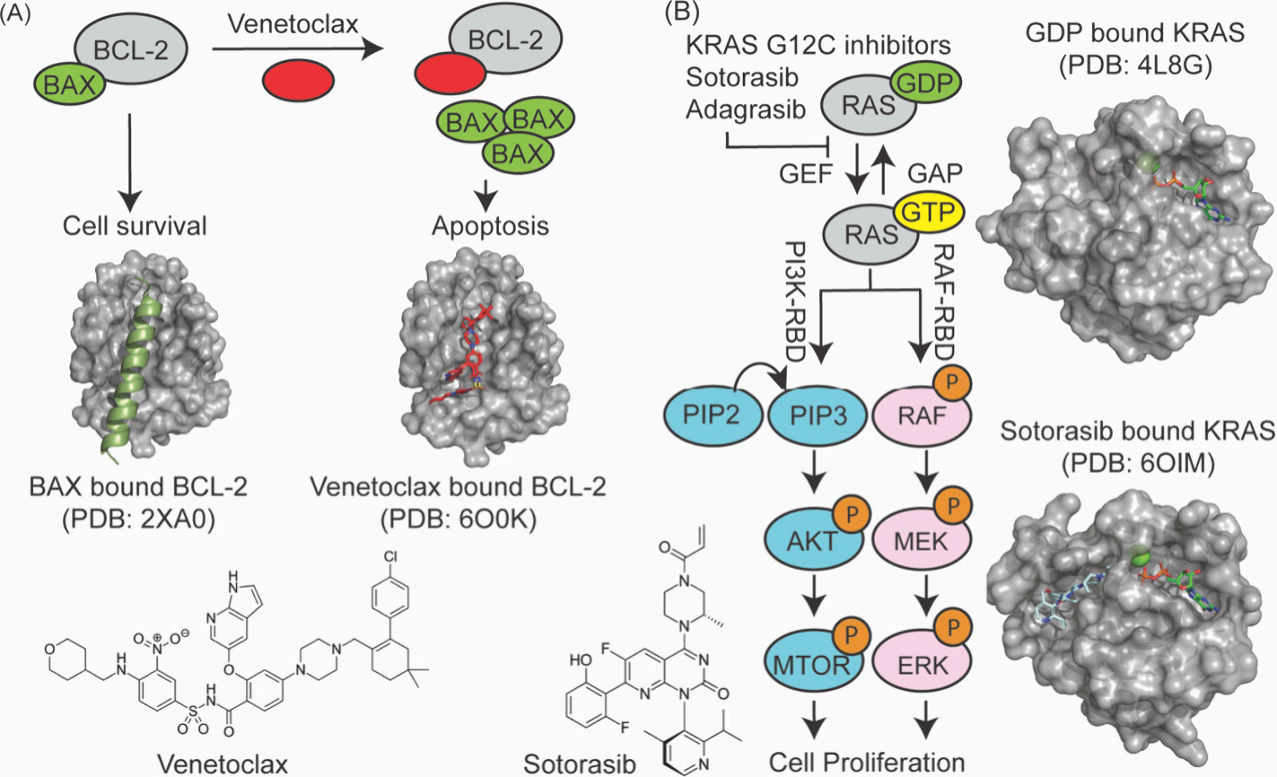
Two clinically successful small molecule inhibitors of PPIs. (A) Venetoclax is designed to mimic a BH3 helix and inhibit association of the antiapoptotic BCL2 protein with pro-apoptotic BH3 family proteins (e.g., BAX). The lead further developed to Venetoclax was discovered using an NMR-based fragment screening assay. (B) Sotorasib is a covalent inhibitor of the G12C mutant of KRAS; this compound was based on small molecules found from a protein-tethering fragment screening approach. Covalent targeting of Ras leads to inhibition proliferative signaling.

This review focuses on the modulation of intracellular PPIs. The extracellular proteins that engage in complex formation with other proteins have proven to be an attractive arena for antibody therapeutics and peptide hormone mimics. Immune checkpoint inhibitors illustrates the success of antibodies and mimics thereof.^19^ Tumor cells avoid destruction by T cells by engaging its programmed cell death protein 1 (PD1) with the tumor cells’ programmed cell death protein ligand 1 (PD-L1).^20^ Complexation of PD-1 to PD-L1, signals the T cell to spare the target cell, allowing the tumor to evade the immune system. Checkpoint inhibitors were developed to inhibit the interaction between PD-L1 and PD1.^21^ Peptide-derived GLP-1 agonists have revolutionized the field of diabetes treatment and obesity and present a classical case of extracellular protein–protein interactions between hormones and cellular receptor.^22,23^ We direct the reader to excellent reviews of recent advances in the modulation of extracellular PPIs modulation by antibodies and miniproteins.^24,25^

### 1.1 *Drugging the Undruggable*: Lessons from Targeting of Ras

Proteomic and bioinformatic analyses have unveiled over 60,000 binary human protein–protein interactions (PPIs) involving 9,094 proteins.^26^ Determining the importance of each interaction at the cellular level or even individual interaction’s function is a complex task. Some interactions or biological pathways are more frequently dysregulated and are more likely to contribute to specific disease states.^27^ Target specific PPI inhibitors can be used as probes to dissect signaling pathways or as tools to discover drug candidates.^28^ Therefore, selection of a target is often driven by biological need; however, chemists may approach a target from a ligand perspective, i.e. they have access to natural products that present certain epitopes, etc.^29^ In an academic setting, protein targets are often selected because (1) they represent an unmet clinical need,^30–33^ (2) the fundamental mechanistic pathway triggered by a PPI has been studied but questions remain that an inhibitor may resolve, (3) proteins can be easily expressed allowing biochemical and screening studies, and (4) structural biology efforts have revealed high resolution structures of individual proteins or their complex allowing rational design efforts. In the following section, we will discuss how these criteria make Ras an attractive PPI inhibition target. Lessons from targeting Ras are likely to be applicable to other challenging PPIs because Ras lacks a specific binding groove, has a range of binding partners, and, significantly, is a dynamic protein receptor.

### 1.2 KRas: A High Value PPI Target with Defined Challenges

In the past decade, Small GTPase Ras protein has been discussed in over 18,500 publications (2014 to 2024 PubMed searched by Title/Abstract). Roughly 2,700 publications have focused on the development of Ras inhibitors during the same period. In comparison, only 155 publications have focused small GPTase Rab inhibitor even though there are a greater number of subfamilies of Rab, and their involvement in various disease prognosis has been reported.^34^ This focused attention on Ras should prompt a set of important questions: (a) What makes Ras such an intriguing target compared to others? (b) Why is it important to discover a PPI inhibitor for Ras? and, (c) What modalities could be employed to discover leads for Ras?”

The Ras protein family consists of four isoforms: H-Ras, N-Ras, K-Ras4a, and K-Ras4b.^35^ All four RAS isoforms consist of two subdomain: the GTPase domain (G-domain) spanning residues 1–166, a short (20 residue) C-terminal hypervariable region that localizes these proteins onto the membrane (Figure 4). The first half of the G-domain is identical in the four isoforms; the second half shares >80% sequence. Although all four isoforms can be mutated, KRas accounts for the vast majority of mutated Ras (>80%) in the solid tumor and represents the most studied Ras protein. KRas mutations are commonly observed in pancreatic ductal adenocarcinoma (PDAC), colorectal cancer (CLC), and nonsmall cell lung cancer (NSCLC).^9,36^ Specifically, PDAC features >90% KRas mutation and PDAC patients suffer from near 80% mortality rates.^37,38^ Excellent reviews have discussed KRas’ role as a oncogenic driver.^9,11^

Therefore, activation of Ras protein or Ras involved PPI network has been studied extensively. Briefly, Ras exists in two membrane-bound forms: a GDP bound *Off* state and a GTP bound *On* state. Activation of Ras protein is initiated by phosphorylation of Receptor tyrosine kinase (Rtk), which is itself activated by different growth factors. Phosphorylated Rtk binds to the SH2 domain of growth factor receptor-bound protein 2 (Grb2), which recruits Son of sevenless (Sos) to the membrane-anchored Ras. Sos is a guanine nucleotide exchange factor (GEF) and activates Ras by catalyzing the exchange of guanosine diphosphate (GDP) nucleotide with GTP.^39^ Association with GTP rearranges Ras conformation and turns it into a substrate for Rapidly accelerating fibrosarcoma (Raf). The Ras-Raf complexation leads to another series of PPIs governing the well-defined RAF-MEK-ERK cell-proliferation signaling pathway.^40^ As a small GTPase, Ras signaling has a self-timer: it is supposed to be turned off by hydrolysis of GTP back to GDP and it is often aided by GTPase activating protein (GAP). However, single mutations in Ras inhibit GTP hydrolysis leading to constitutively active proliferative signaling. Mutated Ras represents a “clean” oncology target because the mutation is directly responsible for the activated signaling.^11,41,42^ Ras thus meets the first two criteria we have listed above: first, mutated oncogenic KRas is a target with unmet clinical need; and second, KRas is a target with a well-defined mechanistic pathway or mechanism of action. Altogether, inhibiting KRas appears to be a promising strategy to provide a therapeutic window for KRas-driven cancers.

Despite its significance in tumor development and the extensive research on its mechanisms, Ras has long been considered “undruggable” mainly due to its unique structural features. First, Ras lacks a prominent groove or deep pocket on its surface that can be targeted to deactivate it. Although there is a binding site for guanine nucleotides (GTP/GDP), design of nucleotide mimics as competitive inhibitors to GTP is not a practical consideration because of the high binding affinity of GTP to Ras (picomolar) and the high cellular concentration of GTP (0.5 mM). In fact, the nucleotide-free form of Ras has only been observed in complex with its binding partner SOS (Figure 4). The structure of the active site without a nucleotide has not been reported, complicating design of competitive inhibitors. However, efforts to target KRas with PPI inhibitors have persisted because KRas is a stable protein that can be easily expressed and high resolution structures of Ras have been available for decades^43^ with numerous biochemical assays described to identify ligands that engage with KRas. Defined structures of interacting partners enable mimics of each domain to be developed as PPI inhibitors. Identification of key interacting residues can be utilized as a starting point for inhibitor design.^10,17^

**Figure 4:**
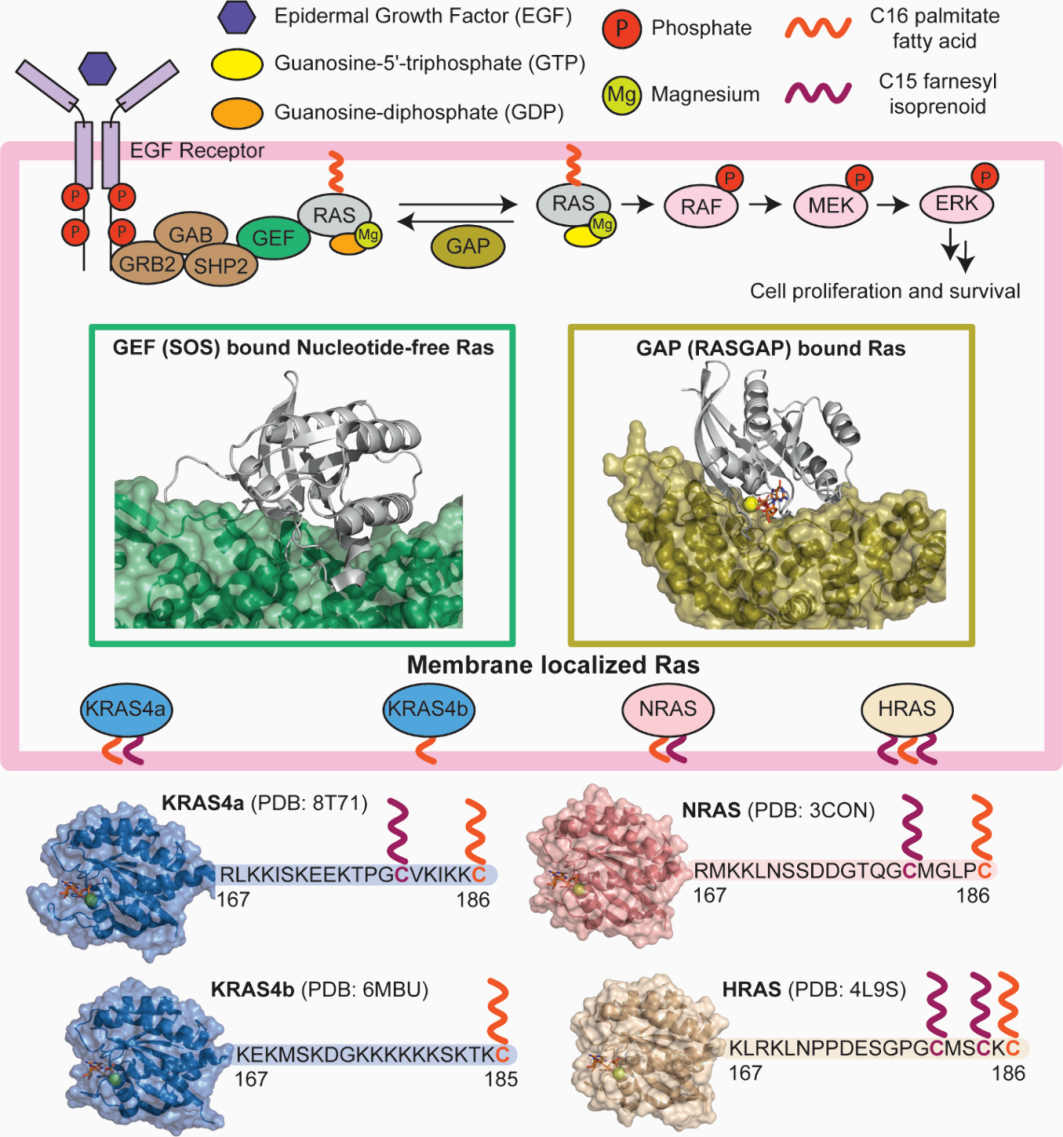
Ras signaling and isoforms. (Top) Ras signaling is triggered by binding of a ligand to the extracellular domain of a growth factor receptor/receptor tyrosine kinase (e.g., epidermal growth factor). Binding of the ligand leads to dimerization and phosphorylation of the receptor tyrosine kinase. Phosphorylation of the receptor creates binding sites for GRB2 that recruits a guanine exchange factor, Sos, to the membrane and in proximity to the receptor bound Ras. Sos catalyzes exchange of GDP nucleotide on Ras to GTP, which triggers downstream signaling. (Bottom) Ras exists in four distinct isoforms: HRAS, NRAS, KRAS4a, and KRAS4b, which are derived from three genes. All four RAS isoforms are nearly identical (>82% sequence identity) in their GTPase domain (G-domain), residues 1–166. The C-terminal ∼20 residues constitute the hypervariable region (HVR) of Ras and are substrates for enzymes that anchor Ras to the membrane.^35^

### 1.3 Targeting KRas: A Protein with Multiple Potential Inhibition Sites and Diverse Classes of Ligands

The longstanding consensus that Ras lacks a conventional binding pocket notwithstanding, numerous unique modalities have been developed to drug KRas. Efforts ranging from traditional small molecules to monoclonal antibody showcase how each type of ligand modality engages unique surfaces on this protein (Figure 5). Small molecules that bind the conformationally dynamic nucleotide binding “switch” regions of KRas protein have been extensively explored across academia and industry (Figure 3B). Pioneering studies by Wells, Shokat, and co-workers utilized a fragment tethering approach^15^ to identify a cryptic pocket that only becomes accessible when GDP is bound to KRas with a G12C mutation.^17,18^ Presence of the nucleophilic cysteine residue enabled discovery of compound 12, which covalently reacts with the protein (Figure 5C). Identification of the cryptic pocket enabled discovery of other electrophiles that to engage reactive residues at the G12X position.^44–47^ Another exciting approach for Ras targeting is represented by RMC-4998, which is a molecular glue that binds to chaperon protein cyclophilin A and covalently links G12C Ras by sculpting its unique neomorphic interface, thereby complexing Ras with cyclophilin A and blocking its effector binding interface (Figure 5D).^48^

**Figure 5:**
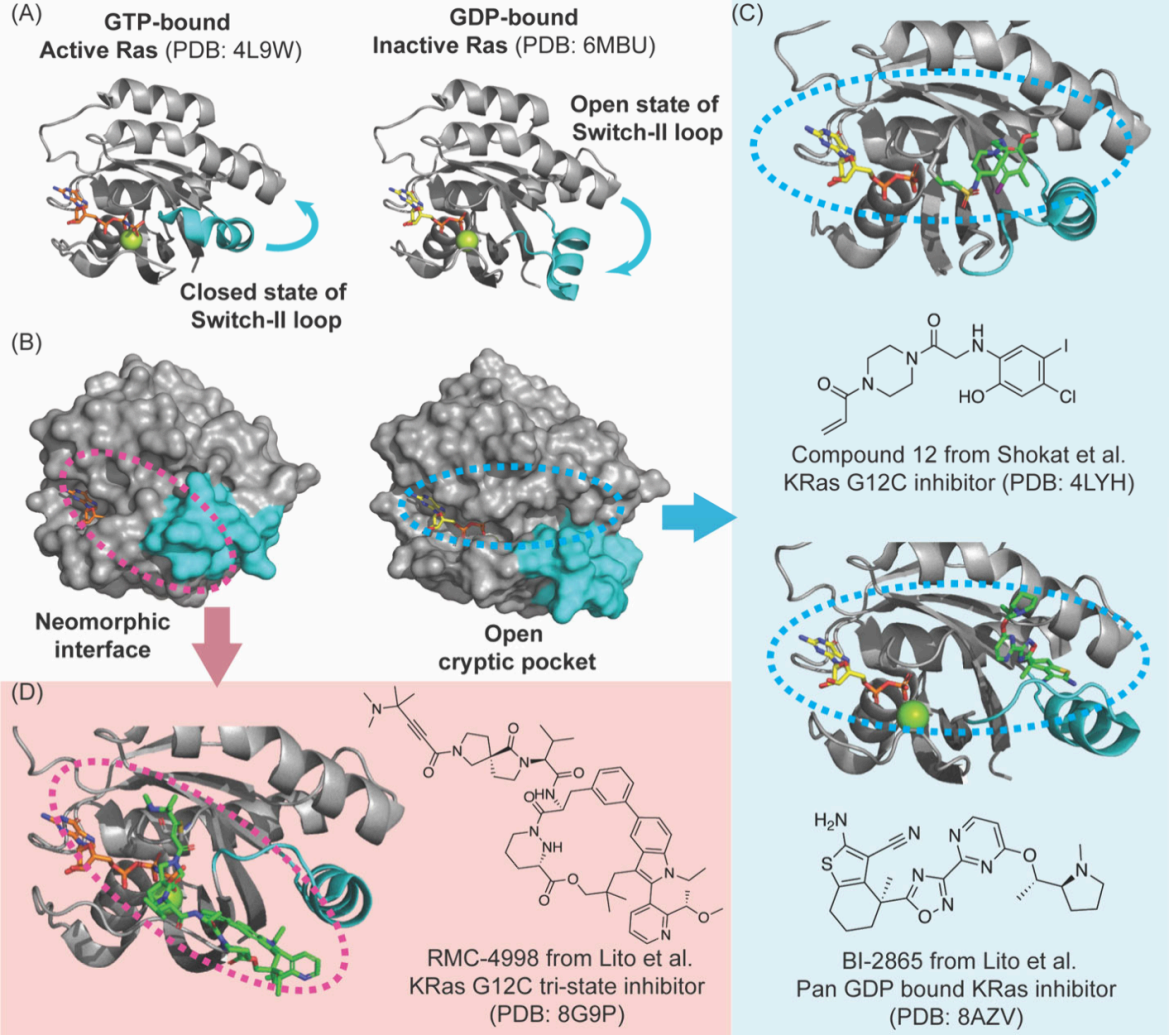
Conformational dynamics of Ras. (A) Structural changes in Ras are intimately linked to its functional state. The switch regions of Ras change conformations between the GDP-bound *off* state and the GTP-bound *on* state. (B) The plasticity of Ras is leveraged by different inhibitors. (C, D) Small molecule inhibitors can access the cryptic pocket or new pockets.

An alternative approach to leveraging cryptic pockets on protein surface to identify binders is to mimic elements of natural ligands of proteins, i.e., other proteins. Since Ras is activated by Sos, a rational approach to develop Ras binders involves designing synthetic protein mimics that replicate the binding epitope of Sos. Sos uses an α-helical hairpin domain to engage the switch region of Ras. Recent efforts from our group have demonstrated that peptides mimicking either a single Sos helix^49^ or the helix dimer^50^ that comprises the helical hairpin can effectively modulate Ras–Sos complex formation and influence Ras signaling (Figure 6).

**Figure 6:**
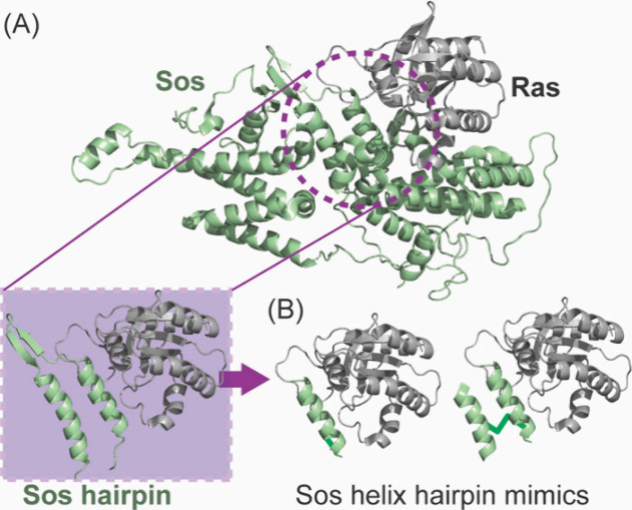
Sos mimics as Ras ligands. Schematic representation based on PDB 1NVW showing (A) Sos utilizes a helical hairpin to engage Ras. (B) Mimics of this helical hairpin have been shown to modulate Ras signaling.

A third approach for the discovery of Ras ligands has focused on macrocyclic peptides and miniproteins isolated from diverse libraries. B4-27, which can selectively bind to GTP-bound forms of wild type Ras, was identified using bicyclic peptide library.^51^ Another cyclic peptide, KRpep-2, was discovered by Tani et al. by utilizing randomized T7 phage display. Crystal structure analysis shows that the KRpep-2 binding site slightly overlaps with those of the small molecules discussed above (Figure 7A).^52^ Cyclic peptide LUNA18, which was discovered using mRNA display technology, also binds the Switch II region (Figure 7B) but induces a different conformational change in KRas than other macrocycles.^53^ Engineered proteins and antibody fragments have also been explored as Ras ligands, although the low cellular uptake of these large molecules remains a liability for intracellular targets.^54,55^ Monobodies, derived from libraries of FN3 domain of human fibronectin domain, and DARPins, which are designed ankyrin repeat proteins, have been shown allosterically modulate Ras (Figure 7C).^56–59^ Similarly, helical miniproteins from the avian pancreatic polypeptide (aPP) were randomized using yeast surface display to target the Ras effector domain (Figure 7C, right).^60^

**Figure 7:**
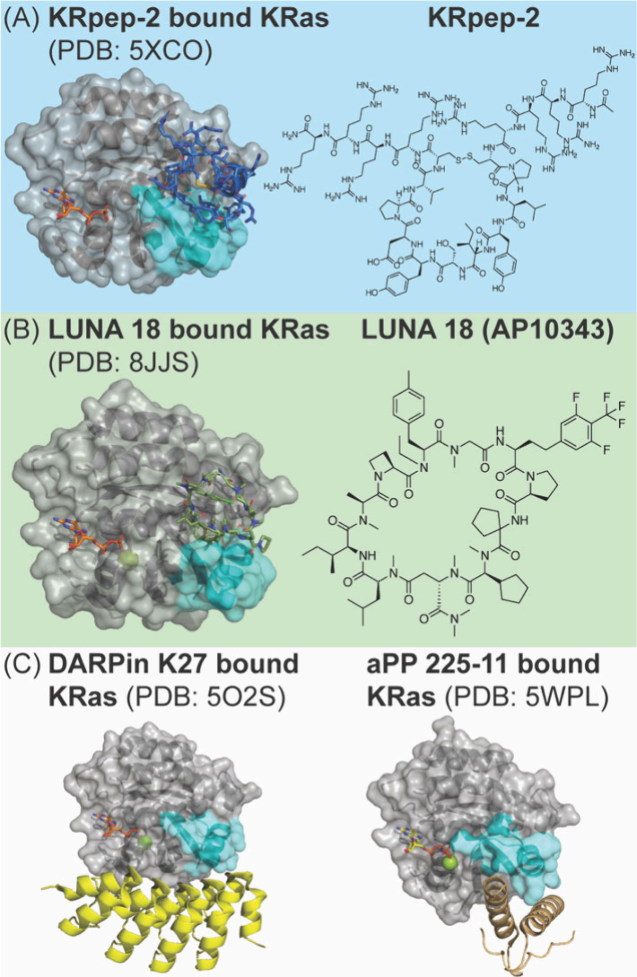
Ras engagement by macrocyclic peptides and miniproteins from libraries. Structure models of (A, B) KRPep-2 and Luna 18 macrocycles, and (C) Miniprotein DARPin and avian pancreatic polypeptide (aPP) show modes for allosteric modulation on Ras.

KRas is no longer considered undruggable. Small molecule candidates targeting the G12C mutant have now entered clinic, while promising leads for other KRas mutations are in advanced stages of development. Several novel therapeutic modalities have emerged, each targeting distinct surfaces on Ras. Small molecules and cyclic peptides primarily focus on the “switch-II” pocket of KRas, while engineered proteins engage “flat and broad” surfaces, suggesting their potential to make multiple contacts on the protein surface. These approaches mark a significant leap forward in KRas-targeted therapy. Allosteric and orthosteric modulation of Ras also illustrates that targeting different surfaces on a protein leads to different outcomes, indicating the critical role of conformational dynamics of protein surface. In the age of induced protein degradation, it is also tempting to consider that rigorous understanding of protein dynamics may be bypassed as long as a highly specific ligand for a protein can be accessed.^61^ In the next sections, we will discuss the improved design and selection methods that have provided ligands for Ras and how these methods will allow discovery of potent inhibitors for other PPIs.

## 2 Are Ras-Targeting Strategies Transferrable to Other PPIs?

Targeting of PPIs presents a conundrum: protein surfaces present at the interfaces typically lack binding pockets required for small molecule binding. In mutant G12C KRas, this challenge was overcome because (a) a cryptic pocket was found and (b) this pocket was near a nucleophilic protein residue for covalent modification. Small molecules are limited in the number of contacts they can make with the target, and a handful of noncovalent bonds do not provide the requisite affinity in the absence of a hydrophobic molecular pocket. One approach for inhibiting PPIs in the absence of deep hydrophobic pockets is to develop covalent inhibitors. Covalent targeting provides a classical drug discovery approach to gaining potency. Several classes of drugs that complex with the target through an irreversible interaction have been reported;^62–64^ however, covalent targeting suffers unique drawbacks. Beyond nonselective reactivity, a challenge with covalent inhibitors is that the nucleophilic protein residue may be mutated away as a resistance pathway. A critical concern with the small molecule covalent ligands for Ras is that resistance is quickly growing to these electrophiles.^65,66^ In the absence of small molecule binding pockets, one approach to identify binding sites on a target protein is to examine its PPIs and specifically focus on those mediated by protein secondary structures. Protein secondary structures are intimate elements of protein folding and structure but also serve as the key recognition epitopes in biomolecular complexes (Figure 8). Interactions of proteins with other proteins, DNA, and RNA are often governed by single secondary structures displaying a handful of contact residues.

**Figure 8:**
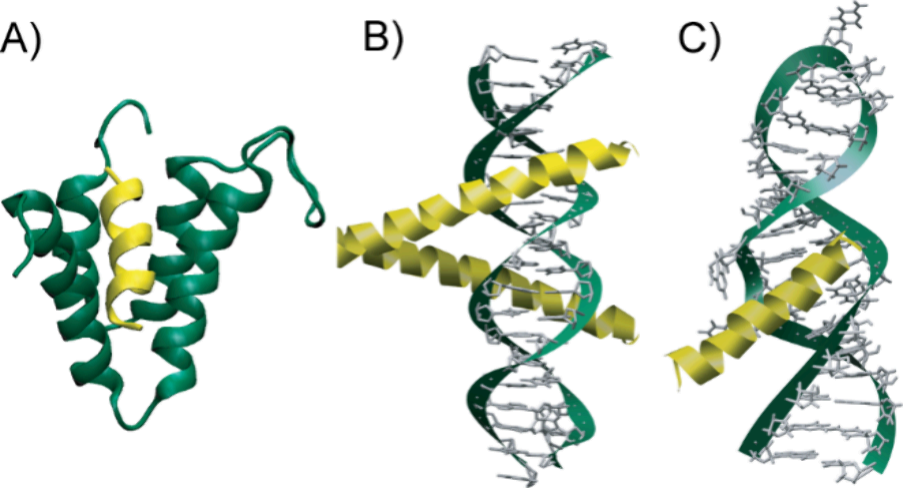
Secondary structures such as α-helices and β-strands/sheets serve as binding epitopes at interfaces of proteins with other biomolecules. The examples illustrate α-helices mediating protein–protein and protein–nucleic acids interactions. Structural models show (A) corepressor Sin3B bound with transcription factor Mad (PDB: 1E91); (B) GCN4 region of leucine zipper bound to DNA (PDB: 1YSA) and (C) HIV-1 rev peptide-RRE RNA complex (PDB: 1ETF).

Analysis suggests that although protein interfaces are large, often a small subset of the residues contributes significantly toward their binding free energy.^67–70^ These “hot spot” residues are commonly located on secondary structures in proteins.^71–73^ It has been demonstrated that synthetic molecules that recapitulate such hot spots can inhibit chosen interfaces with high affinity and specificity.^74–85^ Therefore, identification of PPIs that are mediated by secondary structures provides a potential entry to small molecule PPI inhibitors.

### 2.1 Identification of Inhibitable Protein Complexes from Structural Analysis

The current interest in PPIs as drug targets began with the successful inhibition of model PPIs, using low molecular weight synthetic molecules.^33,86,87^ The p53-MDM2 complex served as the early poster child for these efforts, yielding small molecule inhibitors like MI-219 and Nutlin-3.^88–91^ The BH3/Bcl-2 interaction has also been targeted using small molecule ligands, such as venetoclax.^92–96^ This preliminary success in targeting protein–protein interactions gave rise to an important question: *What types of PPIs are “inhibitable?”* A number of studies have focused on addressing this important question by gauging the “inhibitability” of protein complexes.^97^ Our group focused on computationally analyzing high resolution protein complexes in the Protein Data Bank to identify all PPIs that are mediated by secondary structures (α-helices and β-strands).^98–102^ These analyses used computational alanine scanning as the main metric to define important contact residues.^103–106^

Alanine scanning mutagenesis offers a powerful approach for identifying hot spot residues (Figure 9).^105^ For example, in the well-studied p53/MDM2 interaction, three residues (F19, W23, and L26) from a helix in the p53 activation domain reside in a deep hydrophobic groove (Figure 2B and 9A).^91^ Mutation of any of these residues to alanine leads to a significant (>2 kcal/mol) decrease in the stability of the resulting complex.^107^ Similar alanine scanning results are obtained with pro-apoptotic partners of the antiapoptotic protein Bcl-xL (Figure 9B).^93^ The complex between transcription factor p53 and its regulator MDM2 is inhibited by nutlins (Figure 9C),^89,108^ and that of Bcl2/BH3 by venetoclax and A-385358, an analog of venetoclax^8^ (Figure 9D).^109,110^ The characteristics of these interactions indicate that they can be inhibited with nanomolar affinity by small molecules because the critical residues lie within a small radius of each other on one of the partner proteins, allowing their arrangement on a low molecular weight scaffold. For instance, the two chlorobenzene groups in nutlin-3 span 6 Å, and occupy the binding pockets of the key aromatic p53 residues tryptophan and leucine.^89^ Similarly, A-385358 targets the same key pockets on Bcl-2 as the helical BH3 domains.^111^

**Figure 9:**
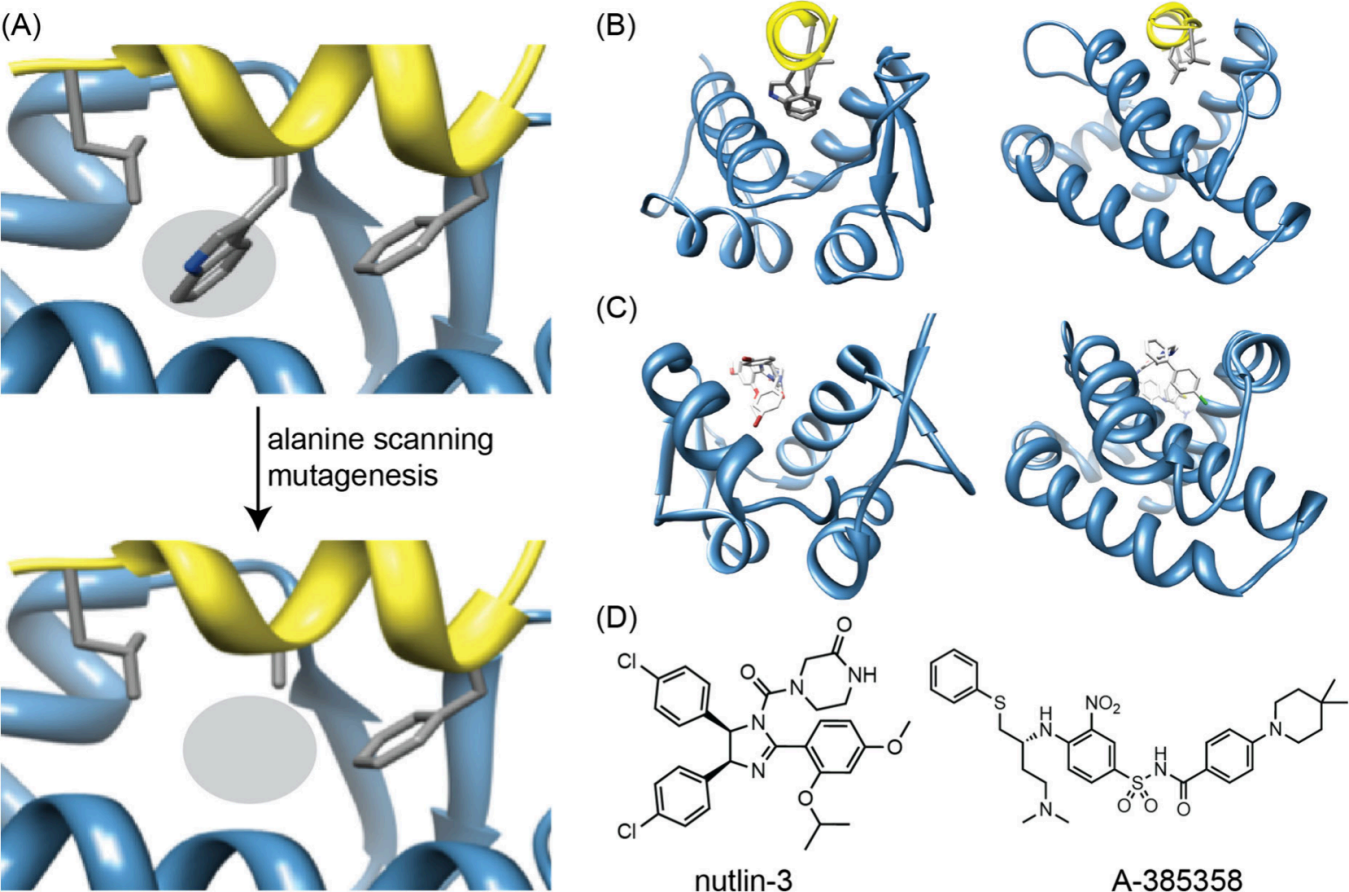
(A) Alanine scanning mutagenesis of interfacial residues reveals the importance of each residue to complex formation. The example depicts mutation of a key tryptophan from the p53 (yellow ribbon) activation helix in complex with MDM2 (blue). (B, left) The p53/MDM2 interaction (PDB: 1YCR). A helix in the p53 activation domain resides in a deep hydrophobic groove. (B, right) The pro-apoptotic protein partner Bak bound to the antiapoptotic protein Bcl-xL (PDB: 1BXL). (C, left) Nutlin-3 binds to HDM2 in the same hydrophobic groove occupied by the p53 helix (PDB: 1RV1). (C, right) ABT-785358 targets Bcl-xL at the site of its pro-apoptotic binding partners (PDB: 2O22). (D) The chemical structures of nutlin-3 and A-385358.

The presence of hydrophobic cavities on MDM2 and Bcl2 evokes protein receptors that can accommodate small molecules. Using these two examples of successfully inhibited protein–protein interactions as a guide, we surveyed the Protein Data Bank (PDB)^112^ to identify protein–protein interactions as likely targets for small molecule inhibitors with the conjecture that interfaces that match the description of p53/MDM2 or Bcl2/BH3. Based on these criteria we identified PPIs that may be inhibited by small molecule peptidomimetics but found that most protein surfaces, as expected, do not fall in the MDM2 or Bcl-2 category and feature extended contact interfaces. As shown in Figure 2C, the Ras/Sos interface is an example of such an extended interface even though the discovery of a cryptic pocket near an electrophile allowed discovery of covalent small molecules.

In the following sections, we describe computational tools that allow assessment of protein interfaces beyond computational alanine scanning. We then describe rational design approaches to mimic protein secondary and tertiary structures to develop leads.

### 2.2 Computational Methodologies to Analyze Protein Interfaces and Design Inhibitors

*In silico* structure-based ligand design has become a powerful tool in investigations of protein function and drug discovery. A continuously expanding number of protein monomer and complex structures isolated through dedicated efforts have greatly aided in PPI inhibitor development.^113–115^ Newly characterized structures are deposited in and made available in the PDB. Structural information on these PPIs can then be analyzed with a growing number of computational approaches to guide inhibitor design.

#### 2.2.1 Computational Alanine Scanning to Identify the Contribution of Native Residues to Complex Formation

Computational alanine scanning is a useful tool for identifying key interacting residues in each PPI. Given structural information on a PPI, a typical computational alanine scanning strategy entails individually mutating each residue on a protein to alanine (Figure 9A). Each PPI structure has an associated binding energy (Δ*G*) that can be calculated using various atomistic parameters.^116–118^ The resulting change in binding energy (ΔΔ*G*) between the native protein and the computationally generated mutant each with the native partner can be evaluated. Key interacting residues such as those previously described in the p53/MDM2 interaction (F19, W23, and L26) mutated to alanine will result in a higher energy complex corresponding to a positive change in binding energy.^119^ A change in binding energy ΔΔ*G* of 1 kcal/mol has been characterized as a “hot spot”.^67,68,120–123^ Inversely, a negative change or no change in binding energy upon mutation of the given residue to alanine is suggestive of a weak interaction; although, a weak contact can be an important contributor to specificity.

A detailed description of binding energy formula and involved parameters for evaluating PPIs is has been well described.^116,117^ Several programs (Rosetta, BUDE, and SSIPe) have been developed to perform computational alanine scanning mutagenesis allowing this technique to be readily available.^124,125^ For a detailed description and comparison of different alanine scanning mutagenesis methods, we guide the readers to this review.^126^ Computational alanine scanning mutagenesis has enabled widescale analyses of PPI structures. For example, our group surveyed all high-resolution protein complexes in the PDB utilizing computational alanine scanning to help identify targetable PPIs with secondary and tertiary structure motifs involved in binding.^102,127,128^

#### 2.2.2 Identification of Cryptic and Underutilized Pockets on Protein Surfaces

Complementarity of protein side chains drives molecular recognition. Alanine scanning mutagenesis provides a method to identify the key binding residues. Emerging *in silico* approaches are also exploring a complementary question: which native interfacial residues can be further optimized to make increased contacts to the target protein? This question is pertinent because nature has not designed all protein–protein interactions to have the strongest possible affinity, thus not all native residues make the best possible contacts. Computational approaches that systematically reveal underutilized contact surfaces provide a powerful tool for rational design (Figure 10).^129–131^ Both natural and non-natural residues have been used to aid in the design of potent inhibitors to optimize native hydrophobic and electrostatic contacts with the protein surface.^132^ The inherent structural plasticity of protein–protein interactions provides a major challenge for structure-based efforts that often utilize static models for inhibitor design.

**Figure 10:**
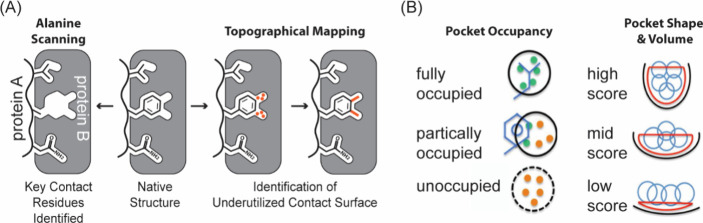
Computational analysis of PPIs. (A) Starting from the native structure, alanine scanning mutagenesis (left) can be performed on protein A to quantify how much each contact residue contributes to the overall binding of the complex. The example shows a phenylalanine residue mutated to alanine to analyze the contribution of Phe to binding. While topographical mapping of the protein B surface (right) reveals underutilized contact surface area, the native residue may not be optimal and a nonnatural amino acid may provide added contacts. (B) Surface mapping allows judicious exploration of nonnatural residues by revealing occupancy of cryptic pockets by the native residues and providing a description of the pocket shape and volume.

Recent efforts with molecular dynamic simulations suggest the exciting possibility of revealing cryptic surface pockets that may be suitable for modulation by allosteric ligands.^133–136^ As such, methods for the detection of cryptic pockets and cavities on protein surfaces are desired. However, identification of druggable protein binding sites is nontrivial. Computational tools such as CAST, FTMap, Q-SiteFinder, AlphaSpace, Fpocket, and PocketPicker have been developed to aid in protein binding pocket identification.^137–145^ Pocket detection and volume calculation is useful in topographically mapping the surface of a pocket aiding in rational design of PPI inhibitors. Designing ligands to fill these high-scoring pockets is predicted to lead more to efficacious compounds.^146^ Non-natural amino acids can be employed to increase the filling of any underutilized pockets.^146^

In addition to pocket identification, computational evaluation and ranking of pockets on a target protein is of interest. Protein complexes often contain a multitude of detected pockets or cavities and ranking these pockets can guide inhibitor design. Pocket ranking involves scoring functions enabling comparison of different pockets to each other. Higher ranked pockets are expected to contribute more to binding if occupied by ligands. These functions are trained on a variety of pocket descriptors such as total volume, concavity, and physicochemical properties.^147^ Elaboration of scoring functions is outside the scope of this work; however, more detailed descriptions of pocket evaluation parameters have been extensively reviewed.^145,147^ Several large databases cataloguing protein pockets found in PDB structures are also available.^145,148^

#### 2.2.3 Protein Docking and Virtual Screening

Mapping of a PPI surface is a powerful strategy for understanding protein binding. Bound protein structures often differ from their unbound counterparts, prompting the use of protein docking to predict complex formation based on the unbound conformations. As our understanding of protein–protein interactions has deepened, computational methods for modeling these interactions have become increasingly sophisticated. Most algorithms are still fundamentally grounded in steric and physicochemical complementarity. The standard protocol typically involves three major steps: (1) a global search of the interaction space using simplified protein representations; (2) refinement to higher-resolution models and localized sampling; and (3) evaluation of the resulting candidate complexes.^149^

Docking is a powerful tool for fundamental studies of protein interactions and provides a structural model for inhibitor design. Computational docking algorithms place the designed macromolecule or fragment into a binding pocket of the target protein and evaluate the relative binding affinities between individual compounds or fragments and the target protein. Ligands can be further optimized from generated poses in complex with target protein in an iterative process. Other computational ligand design tools can be applied such as alanine scanning or pocket identification to inform potential modifications. The redesigned ligand can then be docked and evaluated. Several protein docking programs have been developed such as Glide, Gold, Surflex-DOCK, RosettaDock, and MDock.^150,151,149,152–154^

Virtual screening analyzes an extensive data sets of compounds and predicts a handful of compounds that should be tested.^155^ Advancements in technology have increased computational efficiency, reducing computational cost and time of docking experiments. This has enabled docking of entire ligand databases in virtual ligand screening approaches. Over 1 billion druglike ligands can be screened against a protein structure for potential hits. Docking of these compounds against the protein of interest can identify hits among the virtual library as well as a computational model that can be used to optimize the initial compounds.^156^ To demonstrate the effectiveness and feasibility of such a vast chemical space, an ultralarge virtual library of more than 1 billion compounds was used to identify a lead inhibitor with nanomolar affinity to target Kelch-like ECH-associated protein 1 (KEAP1), shown in Figure 11. The inhibitor, iKeap1, disrupts the interaction between KEAP1 and the transcription factor nuclear factor erythroid-derived 2-related factor 2 (NRF2) and modulates cellular stress response (Figure 11C).^157^ iKeap1 showed structural similarity to a previously identified and structurally characterized NRF2 inhibitor, compound C16 IC_50_ 2.7 μM) isolated from an experimental screen (Figure 11D).^157,158^ The overlap of chemical structure in validated hits identified in different screening efforts demonstrates the effectiveness of virtual ligand screening.

#### 2.2.4 Application of Machine Learning to Computational Design of Inhibitors

Computational tools utilizing PPI structural information can aid in inhibitor design but are limited in scope. Many protein complexes are large and dynamic with transient conformations that may be challenging to isolate and characterize. Computational PPI structure prediction strategies have been developed to guide inhibitor design without experimental structural information. Computational structure prediction of protein monomers and complexes are utilized to generate a model for inhibitor development. These approaches mainly fall into 4 categories: 1) sequence-based templating; 2) structure-based templating and homology modeling; 3) sequence-based templating; 4) machine learning (ML); and deep learning (DL) models.

The current excitement in machine learning on protein structure prediction and design was captured by the 2024 Nobel Prize in Chemistry. Incorporation of neural network architectures trained on evolutionary, physical and geometric constraints of protein monomer and complex structures to protein structure prediction approaches led to the development of AlphaFold and RoseTTaFold.^159,160^ Expanding on the impressive abilities of AlphaFold and AlphaFold2, AlphaFold3 can predict protein complex structures. Generation of accurate computational models of both protein monomers and complexes aids in the development of PPI inhibitors. They can be utilized in molecular replacement and provide a basis for ligand optimization that can be applied to proteins and protein complexes without prior structural information.

**Figure 11:**
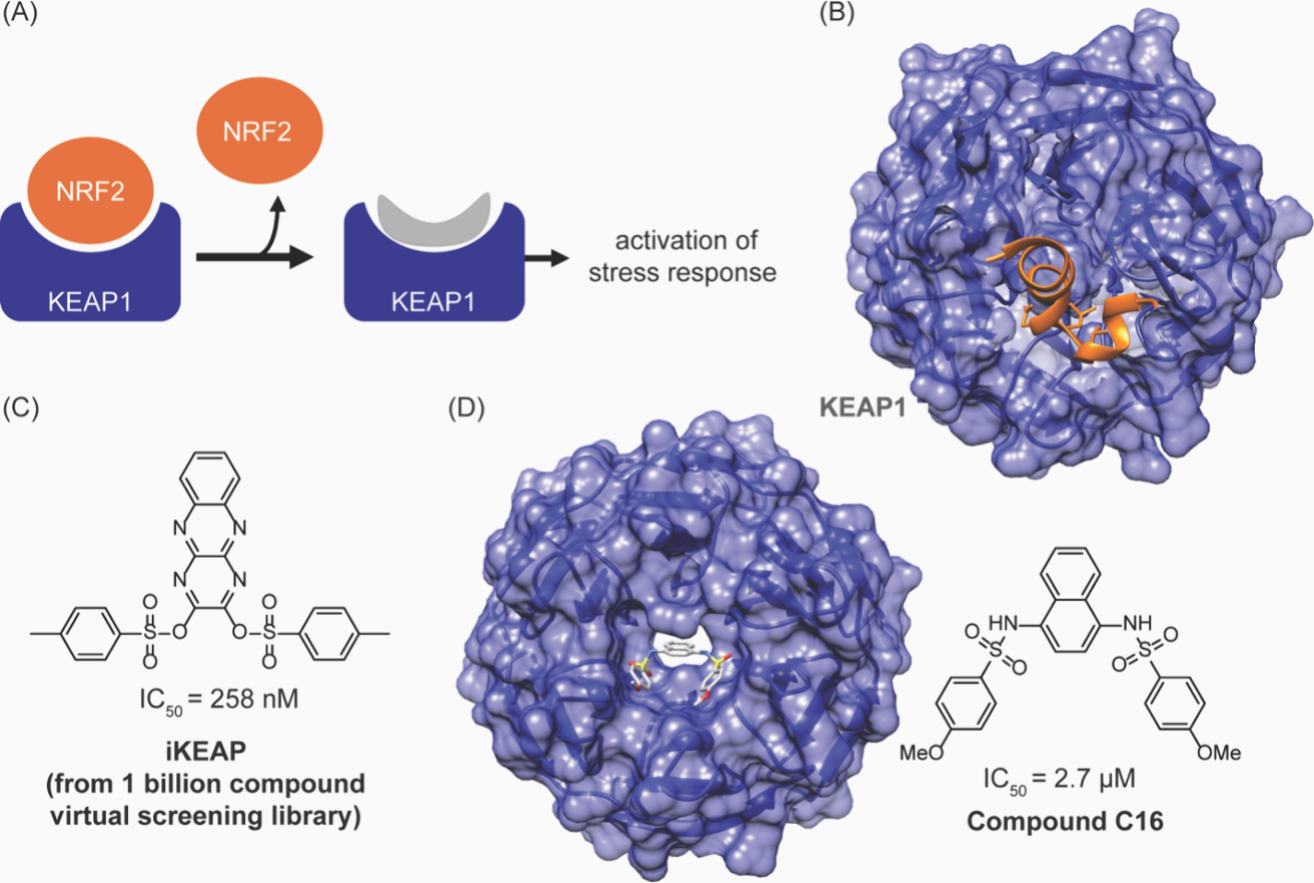
(A) The inhibition of NRF2/KEAP1 interaction is implicated in stress response. (B) Complex of NRF2 and KEAP1 (PDB: 3WN7). (C) A virtual screening workflow that analyzed over a billion compound library identified iKEAP as a lead inhibitor. (D) The structure of the lead compound is similar to a previously identified, and structurally characterized, compound C16, which was identified from experimental screen.

AlphaFold3 introduces the “Pairformer” architecture, a key innovation inspired by transformer models. This architecture processes pairs of amino acids across interacting proteins, capturing the intricate relationships that govern complex formation. By employing a diffusion-based approach, the model iteratively refines predictions to achieve high-accuracy 3D structures of protein complexes.^161^ Simultaneously, this computational approach and advancement pave the way for designing artificial ligands or protein models that can naturally form complexes by leveraging the predicted structural insights and binding interfaces. Baker et al. have developed *de novo* design protocols for the development of macrocyclic peptides and miniproteins ligands.^162–164^ Utilizing these approaches, protein inhibitors of interleukin-6 receptor (IL-6R), IL-6 coreceptor GP130, and interleukin-1 receptor 1 (IL-1R1) subunits with binding affinities in the picomolar to low-nanomolar range were developed (Figure 12). The *de novo* designed miniprotein antagonists prevent binding of key pro-inflammatory cytokines IL-6 and IL-1, which are involved in cytokine release syndrome (CRS). For each of the three interactions a similar design strategy was utilized, which involved analysis of the native interaction for key hydrophobic side chain hot spots. A rigid, virtual miniprotein scaffold library was then docked to the selection of key hydrophobic residues and further refined. Validation of the antagonist design was performed, showing natural interleukin cytokines can be mimicked by smaller and more stable scaffolds.^164^

**Figure 12:**
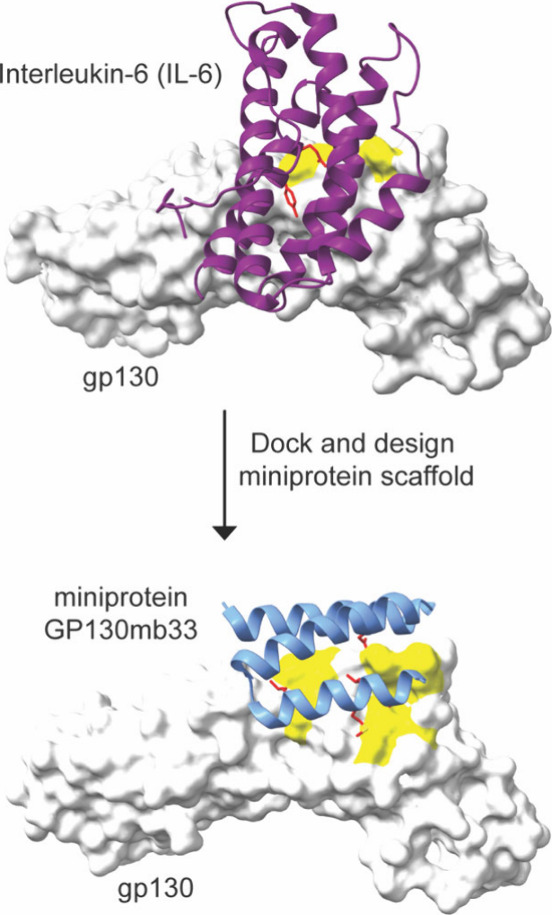
Design of a miniprotein that mimics natural IL6 cytokine. Analysis of gp130 (gray)/IL-6 (purple) reveals two hot spots (shaded in yellow on the gp130 surface). The crystal structure of GP130mb33 in complex with GP130 reveals that the designed miniprotein can engage an extended surface.

*In silico* structure-based ligand design has become a powerful approach for investigating protein function and drug discovery. With the continued growth of experimentally determined protein complex structures, advancements in computing technology, and increased applications of ML and DL methods structure-based ligand design will become increasingly attractive.^165^

## 3 Synthetic Mimics of Protein Secondary and Tertiary Structures as Modulators of PPIs

Natural products are not available as templates to develop PPI inhibitors but nature does offer a template—protein secondary and tertiary structures often serve as the binding epitopes on protein surfaces. Protein mimics can be designed as competitive inhibitors of protein complex formation by capturing a cluster of hot spot residues on a folded domain, as depicted in Figure 13. The secondary and tertiary structures may be envisioned as scaffolds that can be adorned with different side chain groups to engage different protein surfaces. The individual secondary and tertiary structures array residues differently and present unique binding epitopes as depicted in Figure 13B. Conformationally defined peptides have been deployed to mimic and inhibit a range of target relevant protein interactions. Table 1 lists successful examples of protein–protein interactions that have been modulated by peptide engineering strategies. Below we highlight general approaches to develop α-helix, β-sheet, and macrocycle peptide scaffolds.^166^ However, because peptides are inherently vulnerable in biological systems and minimal sequences often fail to adopt well-defined conformations, various scaffolding strategies have been developed to improve peptide stability and enhance their structural rigidity. Classification systems for secondary and tertiary structure mimetics based on the extent of chemical modification have been proposed.^167,168^ An excellent review of designer peptidomimetics and synthetic biologics to target biological complexes was recently published by Moellering and colleagues.^169^

**Figure 13:**
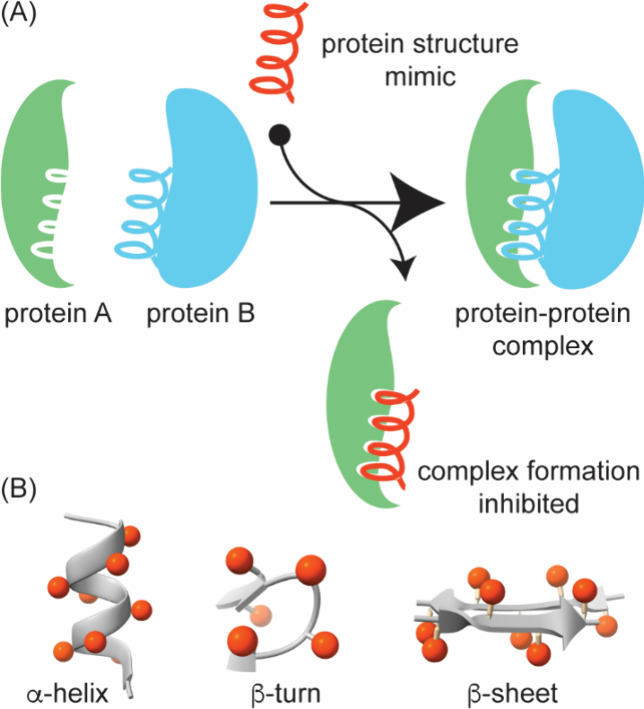
(A) Secondary structures often serve as binding epitopes to mediate PPIs, mimicry of these binding domain by peptido- and proteomimetics offers a rational approach to inhibitor discovery. (B) Secondary structures are scaffolds that display binding residues in different configuration. The array of residues in an α-helix, turn, and β-sheet are shown.

**Table 1:**
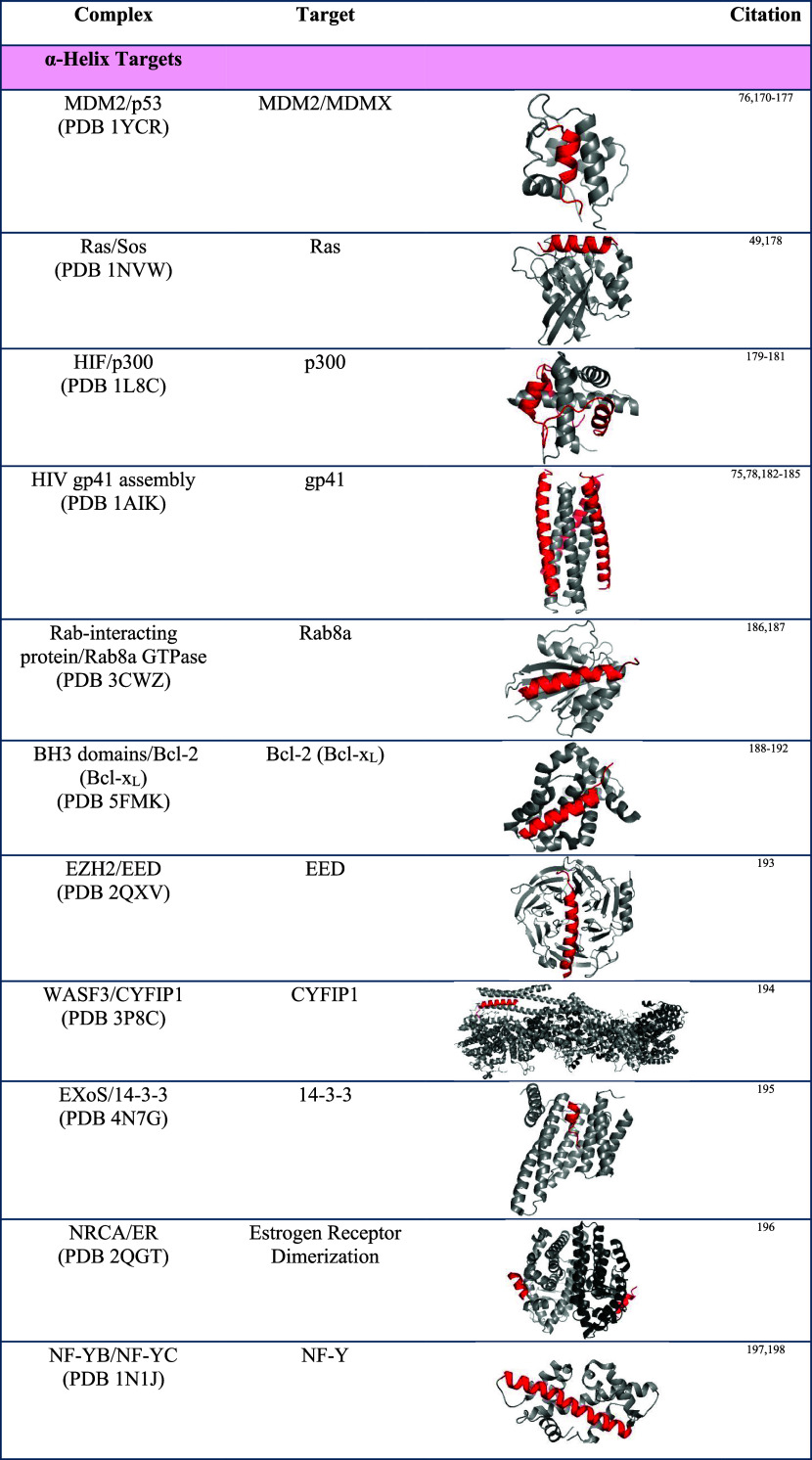
Examples of Protein–Protein Interactions That Have Been Targeted by Secondary and Tertiary Structure Mimics^a^ ^170,170,171,172,173,174,175,176,177,178,179,180,181,182,183,184,185,186,187,188,189,190,191,192,193,194,195,196,197,198,199,200,201,202,203,204,205,206,207,208,211^

a This Table was modified from ref 166 with permission from Wiley.

### 3.1 α-Helix Mimicry

The α-helix is composed of 3.6 residues per turn, resulting in a network of hydrogen bonds between every C=O at the *i* ^th^ position and NH of the corresponding *i*+4^th^ residues. This repeating main chain hydrogen bonding pattern results in the display of side chain functionality on three different “faces” of the α-helix, such as the *i, i*+4, *i*+7, and *i*+11 side chains project from one face. The α-helix is the most prevalent secondary structure and features prominently in molecular recognition of biomolecules. Analysis of helix mediated interactions has revealed that 60% of helical interfaces feature hot spot residues on one face of the helix, one-third feature helices with hot spots on two faces, and roughly 10% require all three faces for interaction with their target protein (Figure 14).^98^

**Figure 14:**
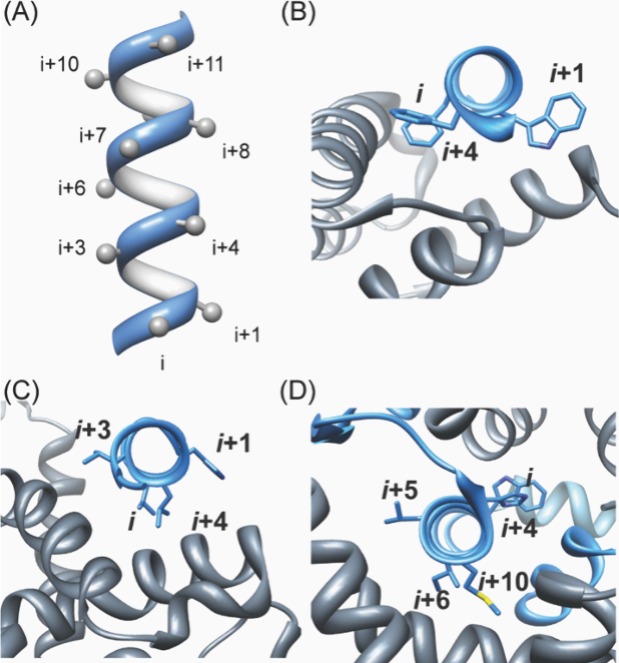
Energetic contributions of residues on different faces of interfacial helices. (A) Positioning of side chain residues on a canonical α-helix, (B–D) examples of protein complexes with hot spot residues on one face, two faces, and three faces (PDB codes: 1XL3, 1XIU, and 1OR7).

Systematic investigation of protein–protein interactions in the Protein Data Bank indicates that the typical length of an interacting α-helix is 8–12 residues, or two to three helical turns.^98,100^ This observation suggests that isolated 8–12 residue peptides, spanning the 900–1400 molecular weight range, that reproduce the native sequence should recapitulate the interaction affinity and specificity observed in the context of the full-length protein. However, short peptides rarely fold into a defined helical secondary structure because the entropic cost of nucleating a turn of the α-helix is not compensated by the free energy gained from main chain hydrogen bonding and various side chain interactions until the peptide length reaches 15–20 residues, depending on the sequence.^213^

The overall helix mimicry approaches can be divided into three general categories: helix stabilization, helical foldamers, and helical surface mimetics.^214^ Helix stabilizing methods based on side chain cross-links and hydrogen-bond surrogates preorganize amino acid residues and initiate helix formation; mini-proteins that display helical domains would also be part of this category. Figure 15 illustrates the different approaches that have been adopted either to stabilize or mimic an α-helix, with the overall aim of developing oligomers with conformational rigidity, proteolytic stability, and the desired array of protein-like functionality.^166^

**Figure 15:**
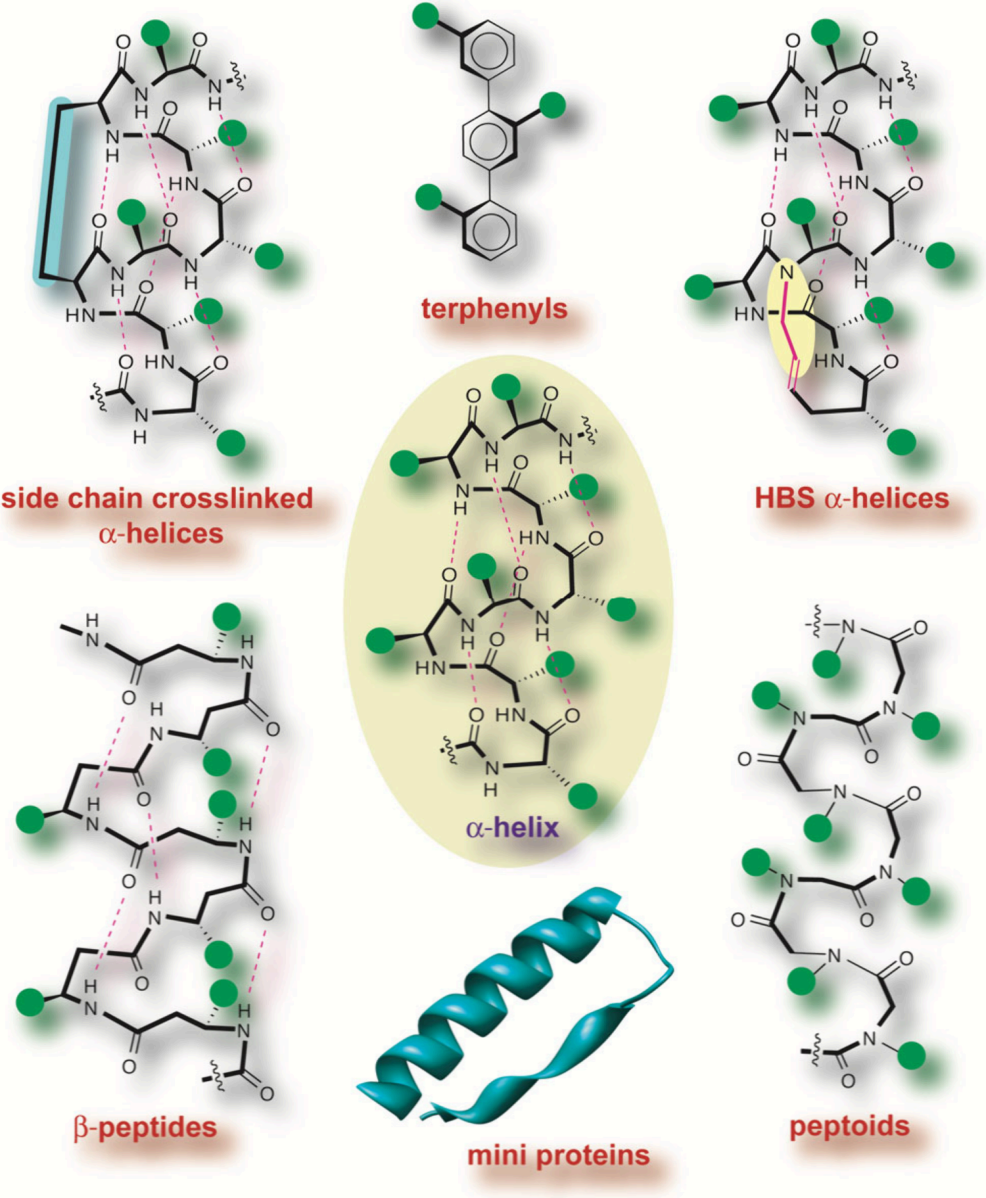
Stabilized helices and non-natural helix mimetics: Several strategies that stabilize the α-helical conformation in peptides or mimic this domain with non-natural scaffolds have been described. Examples include β-peptide helices, terphenyl helix-mimetics, mini-proteins, peptoid helices, side chain cross-linked α-helices, and the hydrogen bond surrogate (HBS) α-helices. Green circles represent amino acid side chain functionality. Adapted from ref 214 with permission from Elsevier.

#### 3.1.1 Helix Stabilization

Experimental studies on protein folding suggest that secondary structures fold rapidly and provide organizational units for tertiary structure formation.^215^ Theoretical models of helix folding have been critical for understanding protein and polymer folding. The helix–coil transition models envision two steps, termed *nucleation* and *propagation*, for α-helix formation, and provide a biophysical underpinning for cooperative folding.^213,216^ These models suggest that three residues—six single rotatable bonds—need to adopt appropriate ϕ/ψ dihedral angles for a peptide to fold into an α-helical conformation. The organization of these three residues results in an α-turn and leads to the formation of a 13-membered hydrogen bond between the *i* and *i*+4 residues (Figure 16). The conformational requirements placed on six single bonds is equivalent to ∼5 kcal/mol entropic penalty but once the α-turn is formed, it preorganizes three carbonyl groups for hydrogen bonding interactions with residues in the next turn. The propagation step in helix formation is enthalpically favored, but >10 intrachain hydrogen bonds are required to pay back the entropic penalty for nucleation. Consistent with these theoretical estimates, experimental studies have shown that peptides shorter than 15 residues do not readily adopt helical conformations.

**Figure 16:**
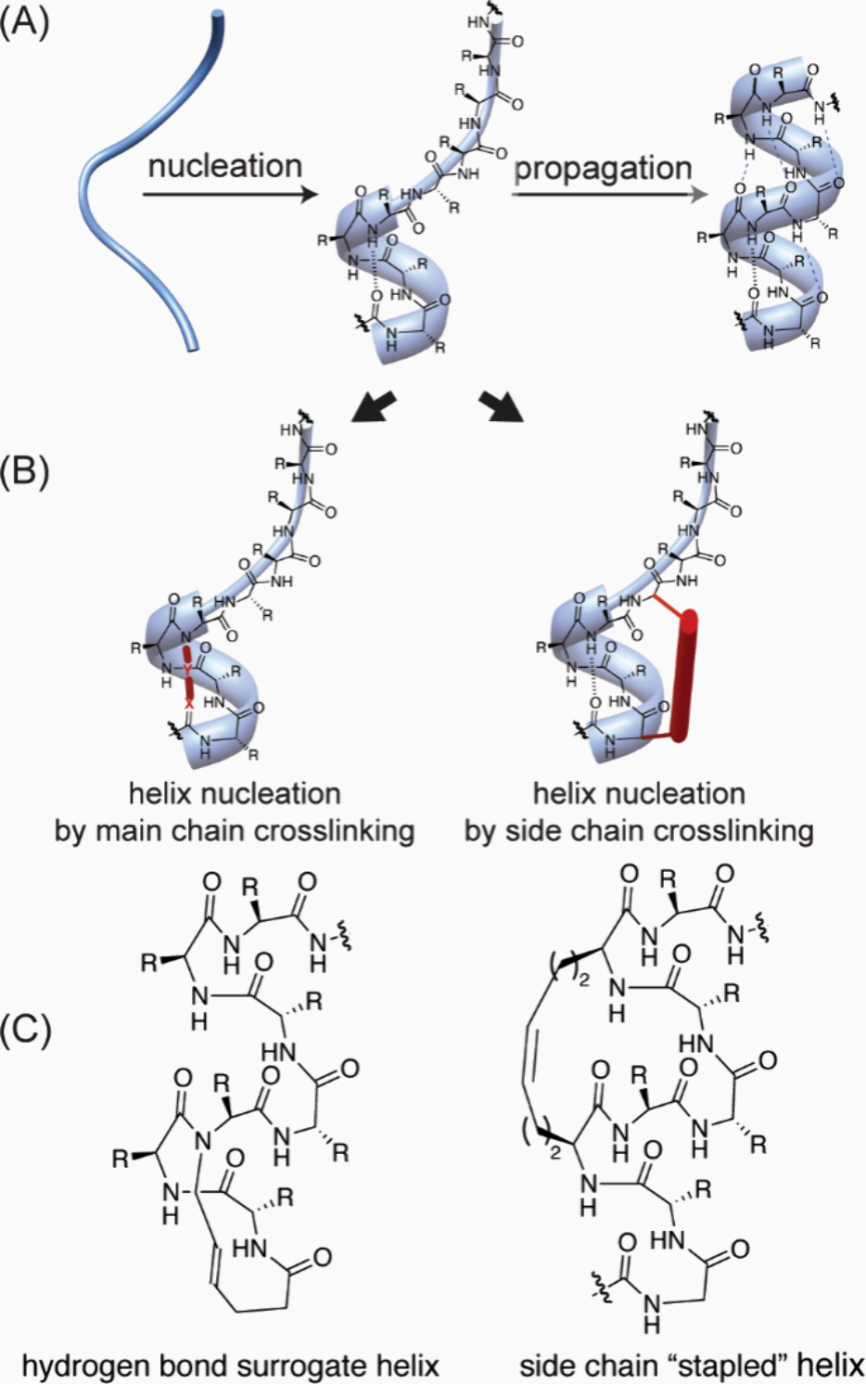
Preorganization of three residues into an α-turn conformation is the energy-demanding step in helix formation. (A) Models of the helix–coil transition consider helix formation to proceed in two steps consisting of nucleation and propagation steps. (B) The helix nucleus can be organized by replacement of a main chain *i* to *i*+4 hydrogen bond with a covalent bond or by cross-linking side chains on one face of the helix. (C) The hydrogen bond surrogate (HBS) and stapled peptides represent two examples of stabilized α-helices.

The helix–coil transition models immediately suggest that covalent constraints may be designed to overcome the entropic barrier and favor helix formation. Two synthetic strategies for stabilizing α-helices are depicted in Figure 16B: (A) replacement of the hydrogen bond formed in the initial α-turn with a covalent bond or (B) cross-linking side chains on the same face of the α-helix. The two helix stabilization strategies suggest the use of covalent bonds to substitute weak hydrogen-bonding or ionic interactions and enforce a folded conformation and both of these macrocyclization strategies have been shown to yield conformationally defined helices.^214^ Specific examples of a hydrogen bond surrogate^217^ (HBS) and side-chain cross-linked helices^218,219^ are shown in Figure 16C. In both examples, the hydrocarbon bridge was obtained by ring-closing metathesis reactions.^220^ Side-chain cross-linked helices generated in this manner are commonly referred to as “stapled peptides.” ^218,221,222^

The two helix stabilization strategies discussed above use covalent bonds in place of weak hydrogen-bonds or ionic interactions and enforce a folded conformation. The side chain stapled helices were first designed in 1988 to substitute a potential ionic contact between lysine and aspartic acid on the surface of a peptide hormone.^223^ Since this early manifestation, several synthetic approaches to cross-link side chain groups, including with biorthogonal functionality, have been described.^81,224,225^ Given their synthetic accessibility, side-chain cross-linked helices have emerged as a dominant strategy in the development of α-helical peptidomimetics, and various reviews have discussed the strategies to design helical peptidomimetics by utilizing the side-chain cross-linking strategies.^224,226^

#### 3.1.2 Helix Foldamers

Two approaches can be envisioned to nucleate a helical geometry in oligomers: (i) the use of constraints to stabilize the peptide conformation or (ii) use of nonnatural residues with a higher propensity to adopt a defined conformation than natural residues. Foldamers are synthetic oligomers that have a high propensity to adopt a folded configuration.^227,228^ Helix foldamers,^229^ such as β-peptides,^230–232^ peptoids,^233^ and AA-peptides,^234^ are composed of amino acid analogs that are capable of adopting conformations similar to those found in natural protein α-helices (Figure 17). Beyond their role in promoting folded structures, foldamers with non-natural backbones offer an additional advantage—their remarkable resistance to proteolytic degradation. Exemplary efforts have led to cyclic and acyclic β-amino acid residues that endow oligomers with conformational and proteolytic stability.^231^ All of these strategies have led to robust mimics of protein α-helices and their applications in targeting PPIs have been extensively reviewed (Table 1).^168,169,235,236^

**Figure 17:**
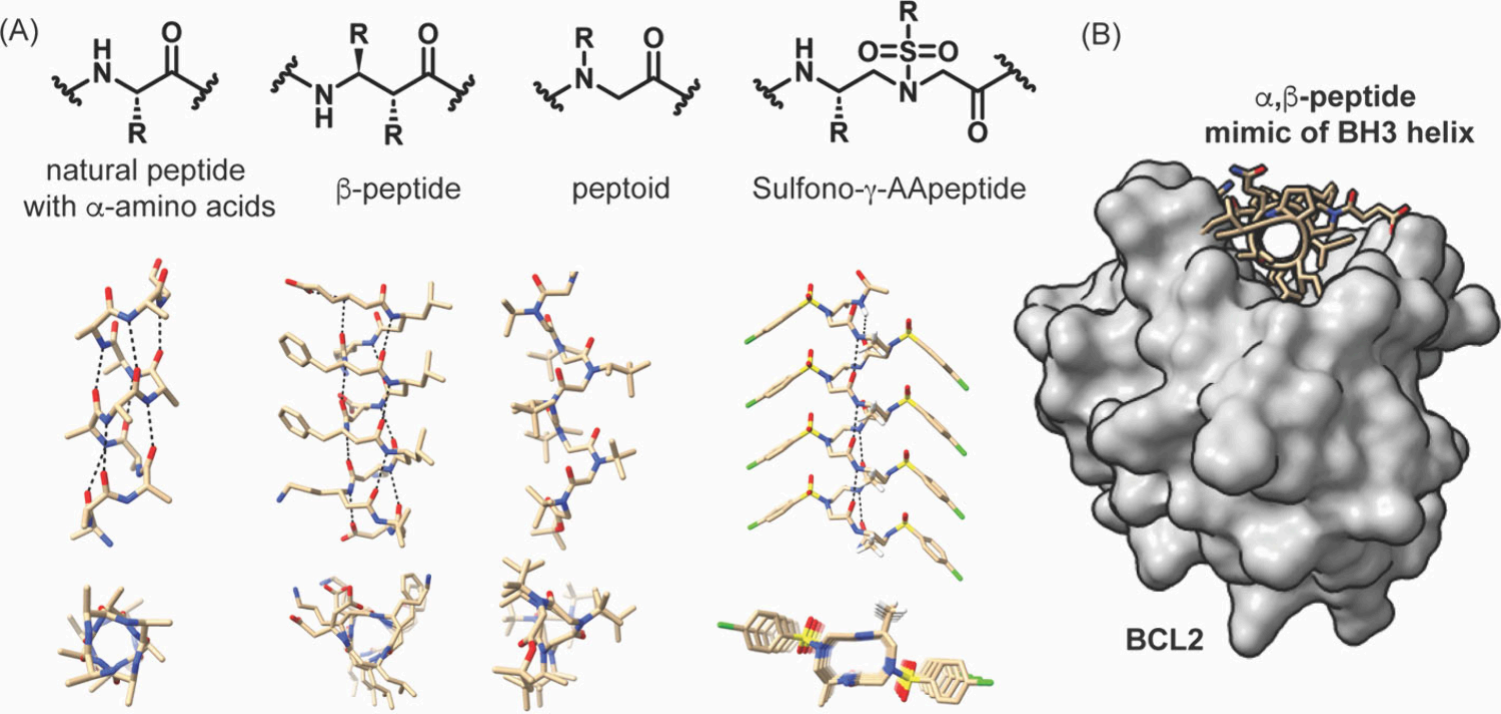
α-Helical conformation can be mimicked by a range of nonnatural oligomers. Some oligomers show a high propensity to fold and are termed *foldamers*. (A) Three classes of foldamers—β-peptides, N-alkyl glycine oligomers or peptoids, and sulfono-γ-AA peptides—are shown. Cambridge structural database accession codes: CCDC 633286, 1561295, and 1841091. (B) Several classes of foldamers have been used as PPI modulators; example shows an α,β-chimeric peptide mimic of BH3 helix bound to BCL2 (PDB: 5AGW).^237^

#### 3.1.3 Helix Surface Mimics

Many proteins (Figure 14) utilize only one face of the α-helix to engage a binding partner, allowing design of minimal helix mimics as inhibitors.^236,238,239^ These minimal mimics, termed helix surface mimics, take capture the functionality of the primary face, the *i*, *i*+4, and *i*+7 residues, of the helix on a nonpeptidic scaffold. Hamilton and co-workers pioneered the development of helical surface mimics with terphenyl and related scaffolds (Figure 18).^240,241^ Molecular modeling and crystal structures suggest that these scaffolds project protein-like functionality in a manner reminiscent of the *i*, *i*+4 (or *i*+3), and *i*+7 positions of a canonical α-helix. Terphenyl derivatives displaying key p53 binding residues were able to selectively inhibit p53/HDM2 interaction *in vitro* with high affinity.^242^ The same group also demonstrated that pyridylpyridone derivatives can effectively mimic the conserved nuclear receptor box motif, LXXLL, and target the interaction of estrogen receptor and its coactivator responsible for the expression of estrogen-activated genes.^243,244^

**Figure 18:**
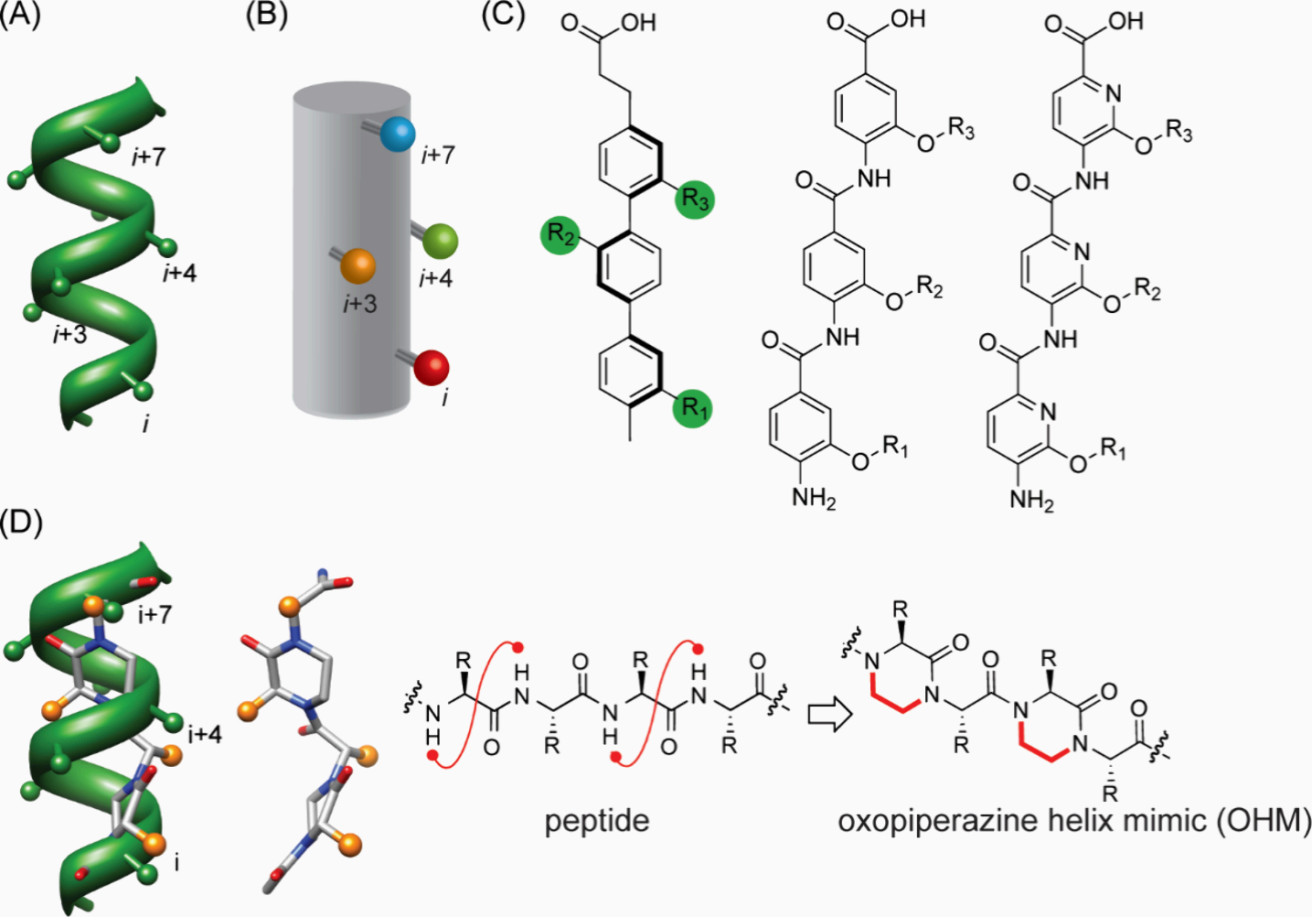
*i*, *i*+4, and *i*+7 residues reside on the same face an α-helix (A). Several classes of small molecule oligomers that mimic the relative placement of these residues (B) are known, including (C) aromatic and (D) nonaromatic scaffolds.

During the past decade, several groups have described helix mimetics that build and improve on the earlier designs with regards to solubility, synthesis, and protein targeting potential (Figure 18C).^236,245–256^ Some of these derivatives have also shown desired activities in cell culture and animal models.^257–261^ Our group sought to develop topographical helix mimics that could be assembled from amino acids to facilitate incorporation of natural and non-natural side chain functionality. Molecular modeling studies suggested that oxopiperazine rings linked by α-amino acids would reproduce the array of side chain residues on one face of a canonical α-helix (Figure 18D).^251^ Figure 19 illustrates two biological applications of these scaffolds. Kumar and co-workers prepared and screened a library of oligopyridylamides as inhibitors of α-Synuclein aggregation. An oligopyridylamide analog was shown to rescue α-Synuclein aggregation in dopaminergic neurons in *C. elegans* models.^262^ Helix surface mimics, as a class, have shown success in advanced biological assays. Figure 19B shows an oxopiperazine helix mimic that reproduces an α-helical domain from the hypoxia inducible factor 1α (HIF-1α). This derivative was shown to inhibit HIF-1α mediated transcription and demonstrated efficacy *in vivo* tumor models.^132^

**Figure 19:**
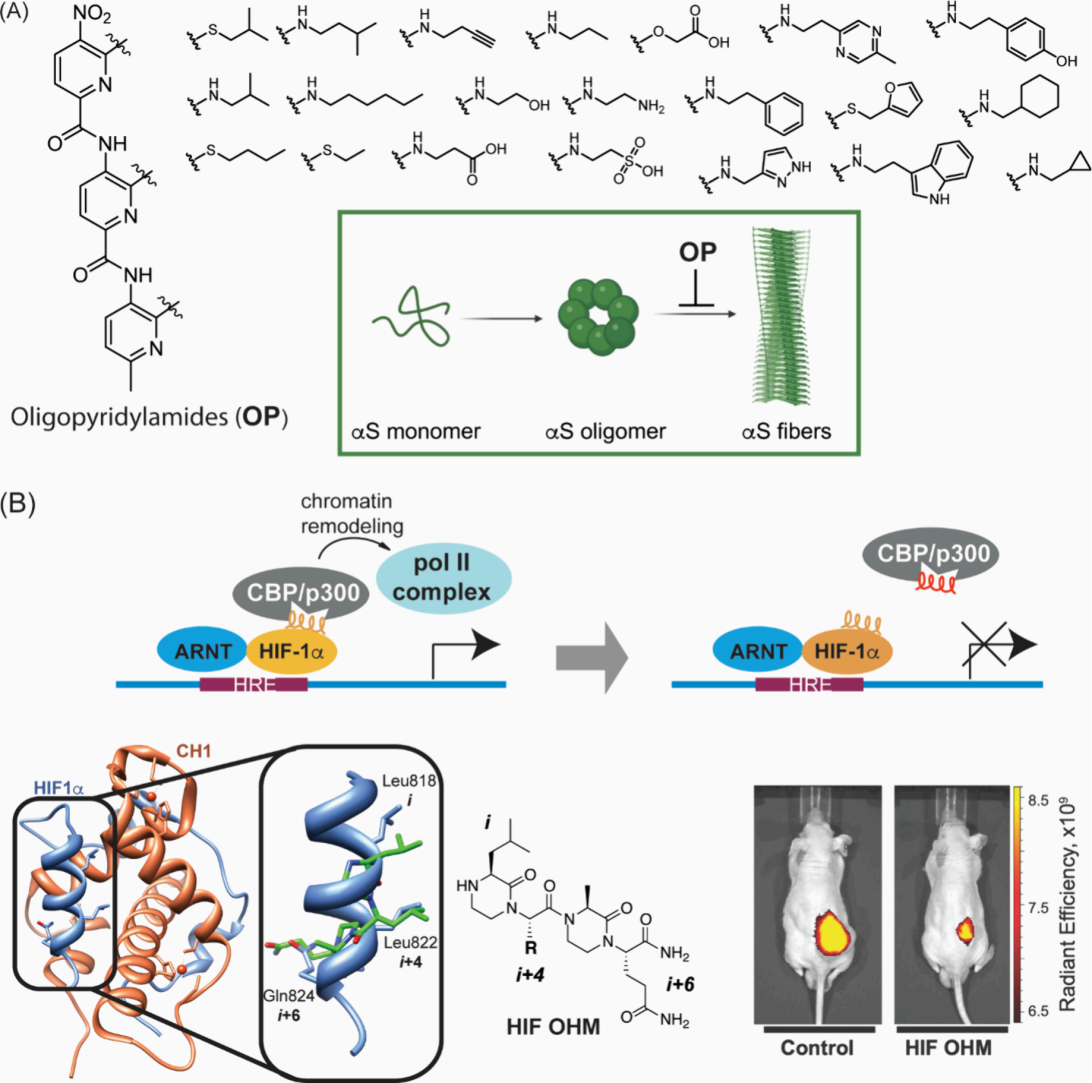
(A) Synthetic libraries of helix mimics can be prepared and screened. Kumar et al. have demonstrated the potential of oligopyridylamides to inhibit α-Synuclein (αS) aggregation.^262^ (B) An OHM mimic of HIF-1α was shown to inhibit hypoxia inducible signaling by disrupting HIF-1α/p300­(CBP) PPI and reduce tumor burden in mouse models.

### 3.2 β-Strand, β-Hairpin, and β-Sheet Mimicry

A β-strand adopts a nearly extended conformation with preferred φ and ψ backbone dihedral angles of −135° and 135°, respectively. This combination of backbone dihedral angles and the uniform L-chirality of amino acids in proteins leads to an overall pleated geometry, positioning every other amino acid on the same side of the β-strand and forming two faces for molecular recognition.^263^ Because the β-strand lacks local hydrogen-bonding interactions that stabilize protein helices, a single β-strand is rarely observed in isolation but are found as components of β-sheets in which β-strands engage in main-chain hydrogen bonding interactions. The β-sheet is a common regular tertiary structure in proteins composed of two or more β-strands.^264^ For any pair of β-strands in a β-sheet, the relative orientation of the peptide termini can be either the same (parallel β-sheet) or opposite (antiparallel β-sheet).^102^ Strands are also stabilized by intermolecular interactions and are observed at protein–protein interfaces offering a rationale for the design of β-strand mimics (Figure 20).

**Figure 20:**
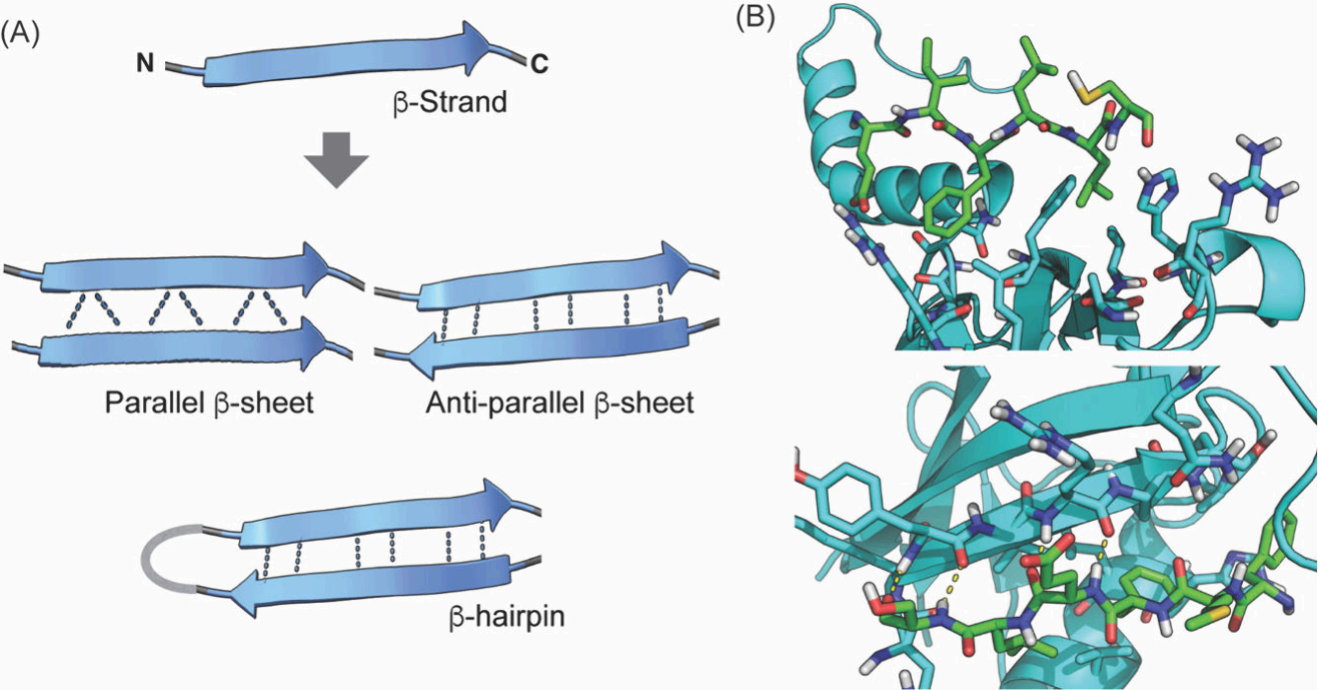
(A) The extended β-strand may be stabilized as part of parallel or antiparallel β-sheets, or β-hairpins. (B) β-Strands can also be stabilized by interactions with proteins by side chain interactions (top, PDB code: 1OY3) or hydrogen-bonding interactions (bottom, PDB code: 4HPM).

#### 3.2.1 β-Strand Mimicry

Innovative strategies to stabilize the β-strand conformation in short peptides have been described.^265^ The general idea is exemplified in Figure 21 and involves constraining individual amino acids, or replacing them with rigid rings, to limit rotations about the ϕ, ψ, and ω dihedral angles. In @-Tide^266,267^ and Hao,^268,269^ an amino acid residue is replaced with a ring with the aim of preserving the extended conformation while aligning the neighboring carbonyl and NH functionalities. The pyrrolinones^270,271^ and triazolamers^272^ consist of heterocycles in place of secondary amide bonds to remove this proteolytically labile unit from strands. Computational and experimental studies suggest that a putative 5-membered ring hydrogen bond stabilizes the β-strand conformation.^273^ This “C-5” hydrogen bond is depicted in Figure 21C. The tetrahydropyridazinedione (tpd) strand mimics attempt to reinforce this intraresidue hydrogen bond while constraining the ϕ and ψ dihedral angles.^274^

**Figure 21:**
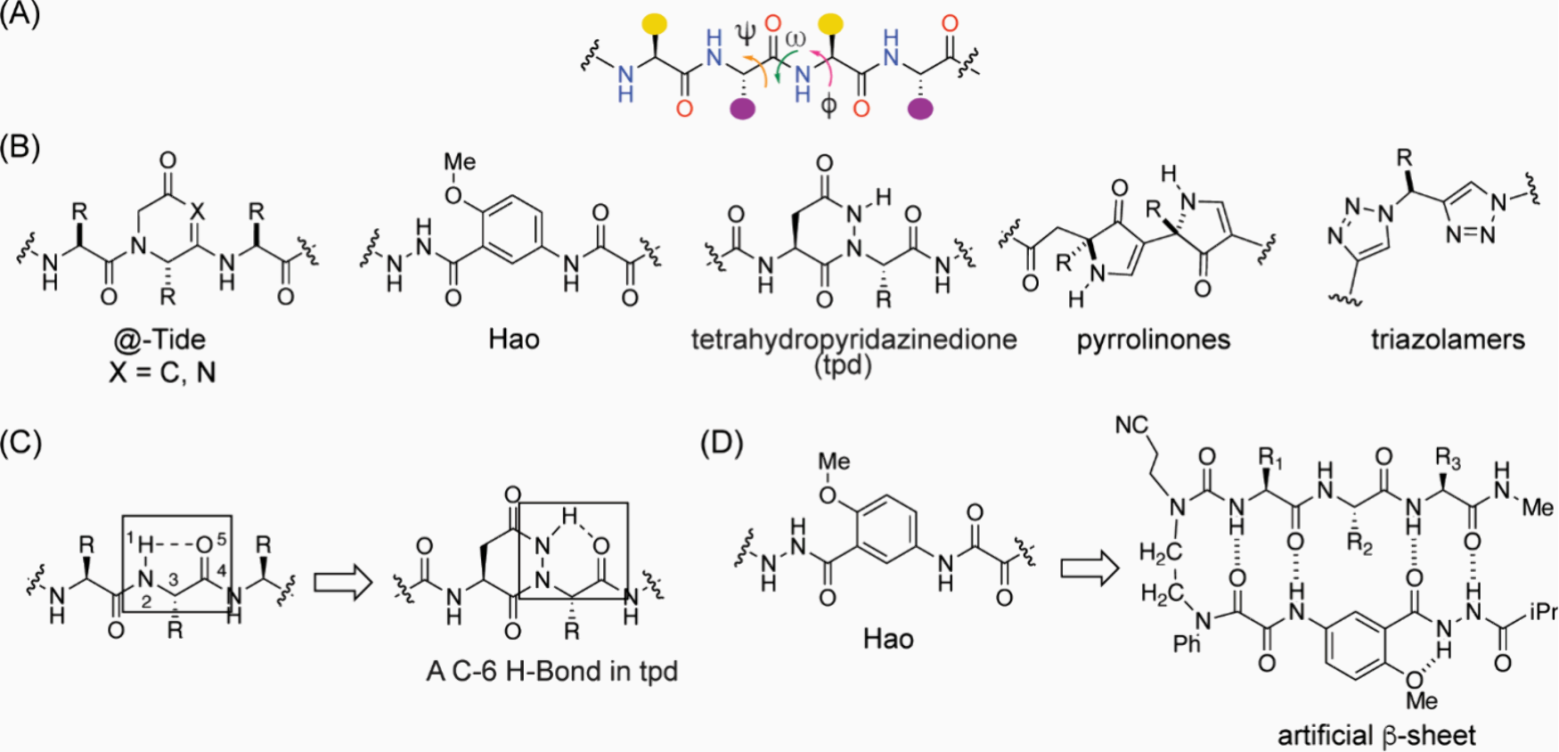
(A) β-Strand stabilization requires restriction of the ϕ and ψ torsion angles. (B) Several cyclic dipeptide mimics have been developed to stabilize the strand conformation in peptides or reproduce this conformation in peptide mimics. (C) A cyclic five-membered hydrogen bond has been postulated to stabilize the β-strand conformations. The tpd unit mimics this hydrogen bond. (D) β-Strand mimics, such as the Hao unit, have also been used to stabilize the β-sheet conformation.

#### 3.2.2 β-Hairpin and β-Sheet Mimicry

The β-hairpin consists of two antiparallel strands joined by a short 2–4 amino acid residue loop region (Figure 20).^275,276^ The stability of this simple motif often depends on the turn residues, the propensity of the residues in the strand region to adopt extended ϕ and ψ dihedral angles, and the side chain interactions between the antiparallel strands. The β-hairpin has two faces: 1) the hydrogen bonding face occupied by side chains from all residues involved in cross-strand hydrogen bonding, and 2) the non-hydrogen bonding face occupied by side chains from all other residues. Early studies on β-hairpins focused on the assessment of folding stability in model sequences.^277–280^ More recent research has sought sequence-independent hairpin stabilization strategies that produce the desired fold while maximizing the number of amino acids available for high-affinity, specific molecular recognition of various targets, including proteins and nucleic acids.^281–283^ In particular, these studies revealed that dipeptide residues ^D^Pro-Gly and ^D^Pro-^L^Pro are strong nucleators of β-turn and β-hairpin conformations.^278,284–287^ These studies inspired a series of synthetic templates that can nucleate β-hairpins (Figure 22).^288^

**Figure 22:**
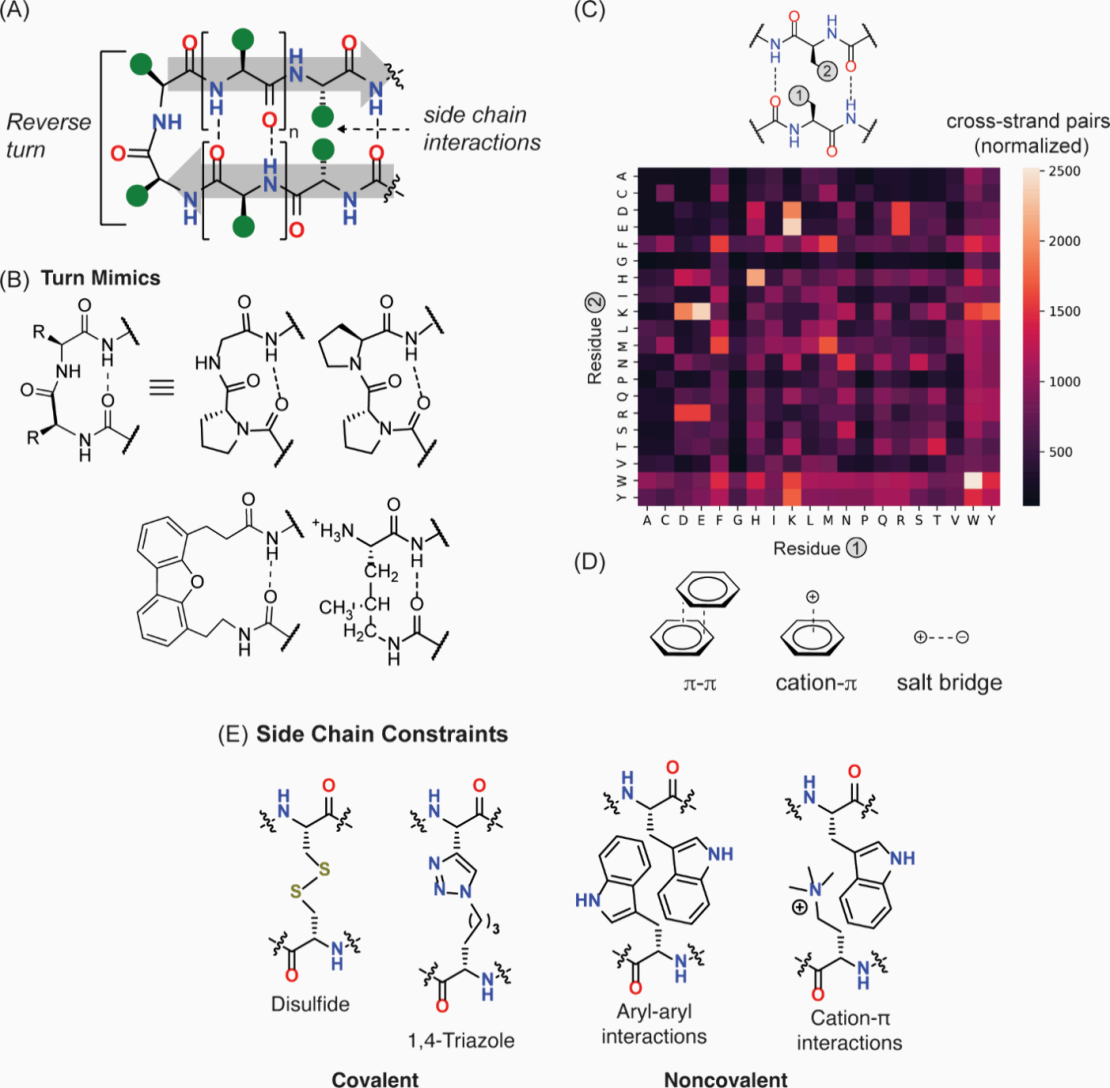
β-Hairpin as a minimal antiparallel β-sheet motif. (A) A β-hairpin features a turn segment that reverses the direction of the strands. (B and E) Extensive studies have provided turn mimics and side chain constraints to stabilize β-hairpin conformation in short peptides. (C) Identification of cross-strand pairs in non-hydrogen-bonded sites within antiparallel β-sheets. Illustration of side-chain interaction between a residue pair at non-hydrogen bonded sites. The plot shows a heatmap indicating prevalence of each pair (normalized for natural occurrence of each amino acid residue). (D) Aromatic interactions, particularly Trp/Trp, cation−π, and ionic interactions are overrepresented in antiparallel β-sheets.

Aromatic residues, especially tryptophan, feature significantly in β-strand-stabilizing noncovalent interactions. In early analyses of amino acid bias in protein secondary structure, it was determined that Phe, Tyr, and Trp are over-represented in β-sheets.^289^ This analysis of cross-strand side chain interactions in β-sheets has been previously described; however, these analyses were performed on an older versions of the Protein Data Bank with limited entries.^290–293^ We interrogated the current PDB to understand the prevalence of natural amino acid pairs at non-hydrogen bonded sites.^292,294^ Our bioinformatic analysis of antiparallel β-sheets^295^ in the PDB shows that cross-strand aromatic, salt-bridge and cation−π interactions are prevalent, in keeping with the earlier studies on β-sheets and proteins overall (Figure 22).^296^ Extraction of cross-strand interacting pairs at non-hydrogen bonded sites and normalization for natural occurrence of each residue is plotted as a heat map in Figure 22C.^294,297^ We found that tryptophan pairs are over-represented as much as ionic interactions on a normalized basis in keeping with tryptophan pairing’s rich history in β-hairpin/β-sheet design.^279^ Aromatic cross-strand interactions have been extensively studied in β-sheets and β-hairpins.^298–300^ Researchers at Genentech designed the “tryptophan zipper,” or trpzip, that showed remarkably high β-sheet conformational stability.^279^ In these and subsequent studies,^301^ it was shown that aromatic residues at non-hydrogen bonding positions prefer to stack in a stabilizing edge-to-face geometry, giving rise to unique spectroscopic signatures by NMR and circular dichroism. Andersen et al. later developed a shorter stable β-hairpin called HP7 using this approach.^302^ In a similar vein and taking inspiration from cation−π interactions in natural PPIs such as bromodomains, the Waters group has shown that interactions between alkylamine-bearing amino acids and Trp stabilize β-hairpins when they are placed at opposite positions in a model β-hairpin.^303^ Amine methylation and side chain length both strongly impact the stabilizing cation−π effect on conformational stability.

The recognition that side chain interactions are critical for β-hairpin stability, paved the way for the insertion of covalent cross-links into these constructs. Common covalent cross-links include cystine disulfides from cysteine residues^304^ and 1,4-triazole linkages formed between azide- and alkyne-bearing side chains using copper­(I)-catalyzed azide–alkyne cycloaddition (CuAAC).^305,306^ Both the cystine disulfides and triazole cross-links stabilize β-hairpins when the residues are across from each other (Figure 22E).

Combinations of these strategies have recently been employed to generate stable β-sheets that do not require a reverse turn to connect the β-strands. Andersen et al. demonstrated that peptides containing a central cysteine disulfide and terminal cation−π capping interactions fold into highly stable β-sheets.^307^ Our group also showed that reverse turns can be swapped with hydrogen bond surrogates that lead to conformationally defined HBS β-sheet scaffolds.^294^

#### 3.2.3 Application of β-Hairpin Mimics as PPI Inhibitors

Fundamental studies on β-hairpins have revealed methods for the stabilization of these motifs, which constitute the smallest β-sheet designs. The application of β-hairpins and β-sheet mimics as PPI inhibitors has been hampered by due to the intrinsic properties of these peptides to self-assemble into aggregates.^308^ Indeed, β-hairpins provide excellent components to rationally design hydrogels.^309^ β-Sheets also self-assemble into amyloids that are observed in a range of neurodegenerative diseases. β-Hairpins would form ideal ligands to block aggregation of β-sheet assemblies through intermolecular hydrogen bonding and side chain interactions but they can also nucleate aggregation if they engage in interactions on both strands. To address this conundrum, Nowick and co-workers designed a macrocyclic β-hairpin analog that can only participate in intermolecular contacts on one strand by blocking the second strand with a non-natural component (“Hao”) that lacks hydrogen bonding potential on one face (Figure 23). These hairpin blockers have shown success in antagonizing amyloid aggregation.^310^ The group of del Valle and co-workers used a similar concept in blocking Tau aggregation; this group incorporated an amino group^311^ in place of the amide hydrogen to remove a hydrogen bond donor on one face of the hairpin (Figure 23B).^312^

**Figure 23:**
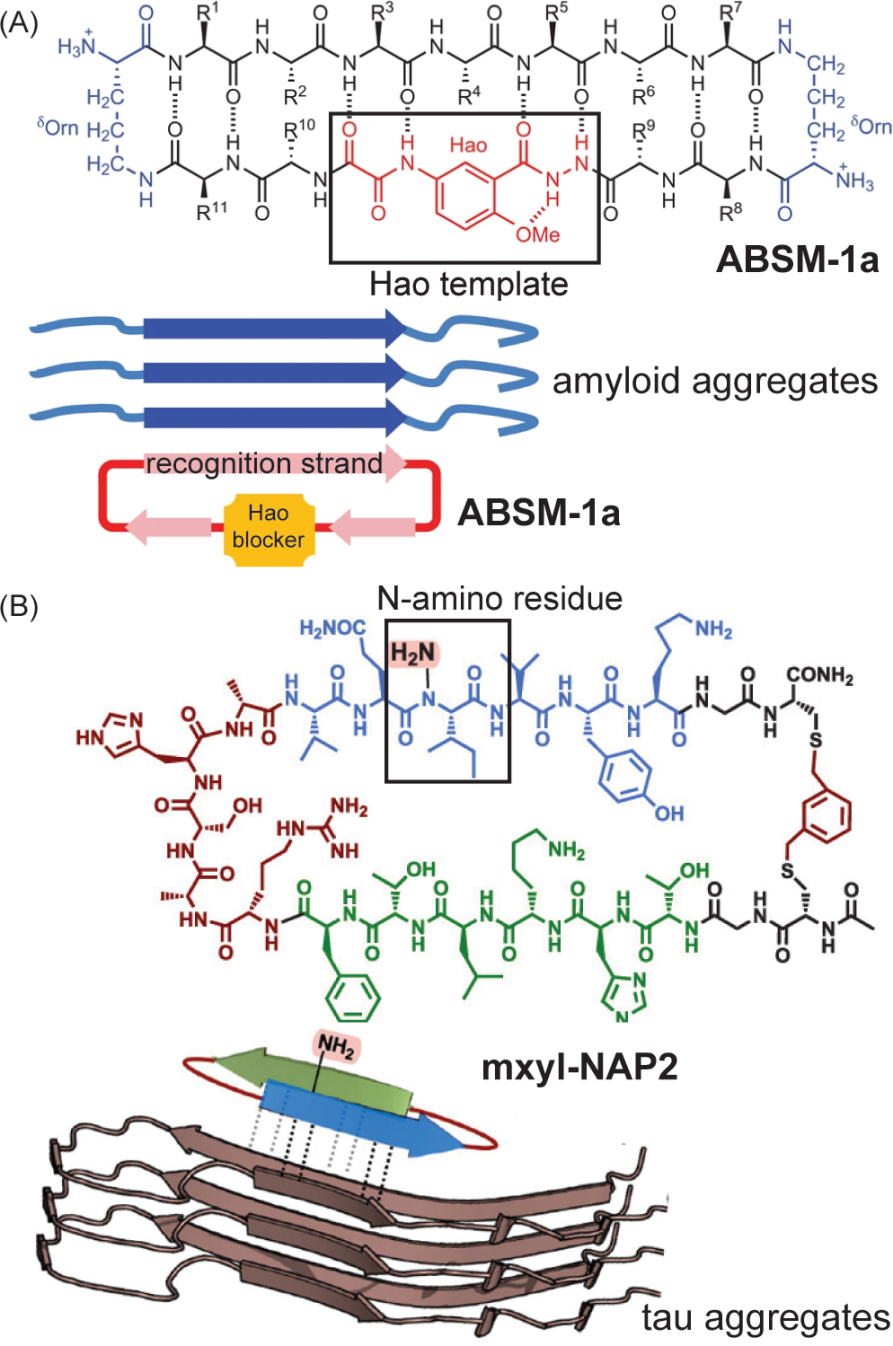
Strategies to develop macrocyclic hairpin analogs that block protein aggregation. Both strategies incorporate nonnatural groups (ABSM-1a (A) and N-amino acid residue (B) to block hydrogen bonding on one face of the hairpin. Figure adapted from refs 310 and 312.

There has been a growing interest and success in targeting intracellular PPIs with synthetic β-hairpins. In two recent examples, macrocyclic ligands for protein β-catenin have been described (Figure 24). β-Catenin is a transcriptional coactivator that acts as a hub for PPIs within the Wnt signaling pathway.^313^ Hyperactivation of this pathway leads to abnormal cell growth and cancers^314^ through β-catenin’s engagement of the T-cell factor (Tcf) family of proteins.^315^ Thus, far, small molecule efforts have not translated to potent inhibitors of this interaction providing a rationale for peptidomimetic inhibitors.^316,317^

**Figure 24:**
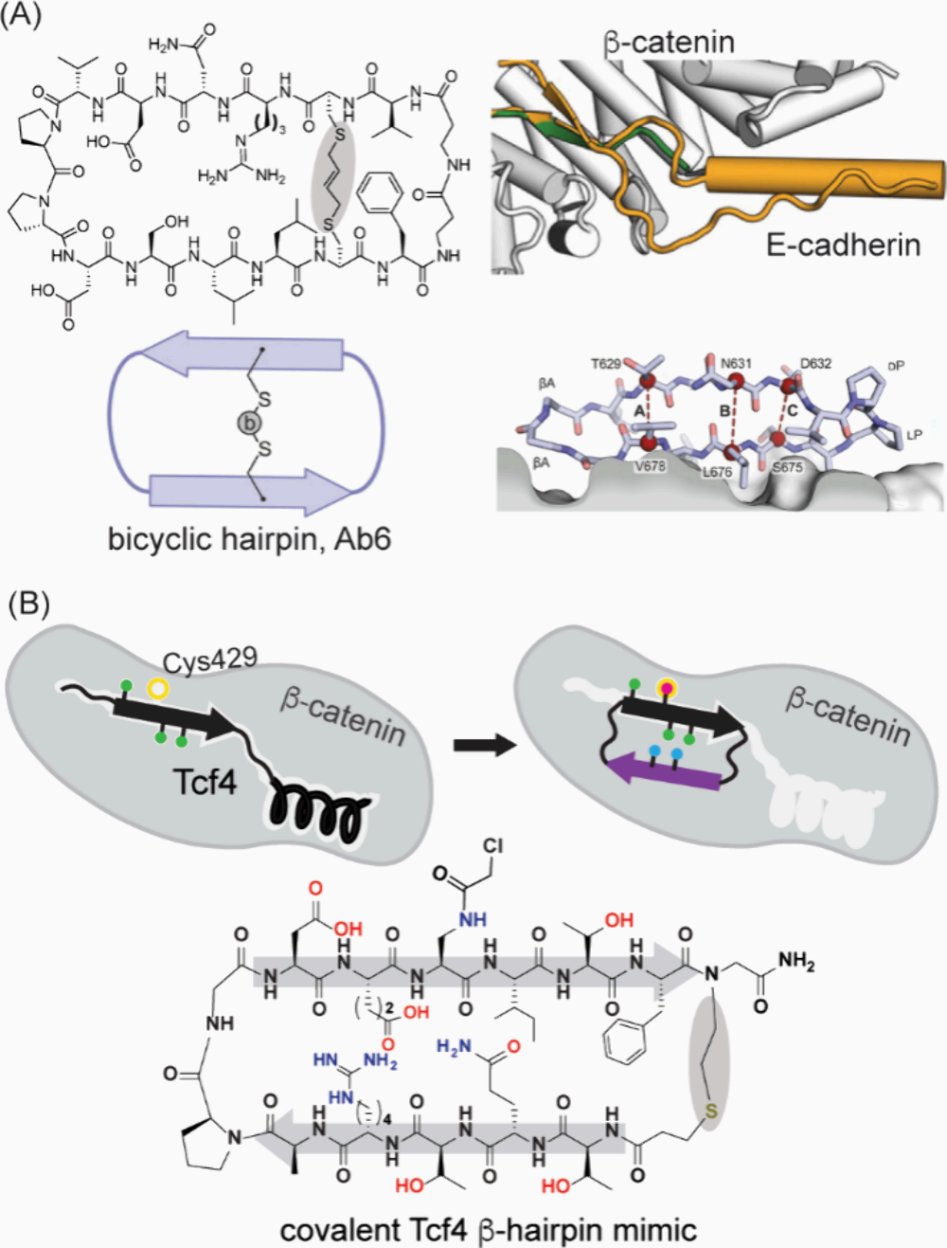
(A) A bicyclic hairpin peptide that mimics E-cadherin β-sheet. (right) Crystal structure of E-cadherin (PDB: 1JDH) and bicyclic macrocycle bound to β-catenin. (B) Design of a covalent β-hairpin based on Tcf4 sequence mode; the extended segment of Tcf4 was stabilized as part of a hairpin. Figures adapted from refs 210 and 209.

The Grossmann group designed β-catenin ligands based on an E-cadherin antiparallel β-sheet segment that interacts with β-catenin at the same site as Tcf4.^210^ Macrocycles stabilized through a rigid (^D^Pro-^L^Pro) and a flexible (dibeta alanine) turns yielded an E-cadherin antiparallel β-sheet inhibitor (Figure 24A). Crystal structure of the macrocycle bound to β-catenin shows site-specific engagement of the macrocycle on β-catenin. In addition to macrocyclization and the rigid β-turn, the E-Cadherin-derived macrocycle required side chain cross-linking for optimal target engagement. Our group recently reported covalent macrocyclic β-hairpin inhibitors for the same target.^209^ We designed the β-hairpin to mimic one strand of Tcf4 bound to β-catenin (Figure 24B); the Tcf4 strand was stabilized by a second designed strand as part of a β-sheet conformation. We chose Tcf4 residues that lie in proximity to two cysteine groups on β-catenin providing an opportunity to develop a β-hairpin that engages the target through covalent capture. The binding site for the designed hairpin was identified by mass spectrometry analysis.

### 3.3 Loop Mimicry

Loops are nonregular protein structures that are commonly observed at protein interfaces.^318^ The term “Non-regular structure” encompasses a diversity of conformations that lack local repetition of backbone dihedral angles as observed in α-helices and β-strands/sheets. A survey of the Protein Data Bank by Kritzer et al. identified loops that make important energetic contacts in mediating protein–protein interactions involved in a range of functions.^319^ Loops also play critical molecular recognition roles in antigen–antibody and other protein complexes.^320^ Loops have been classified in different categories based on backbone ϕ/ψ and χ angles and hydrogen bonding between side chains and the main chain (Figure 25).^319,321^

**Figure 25:**
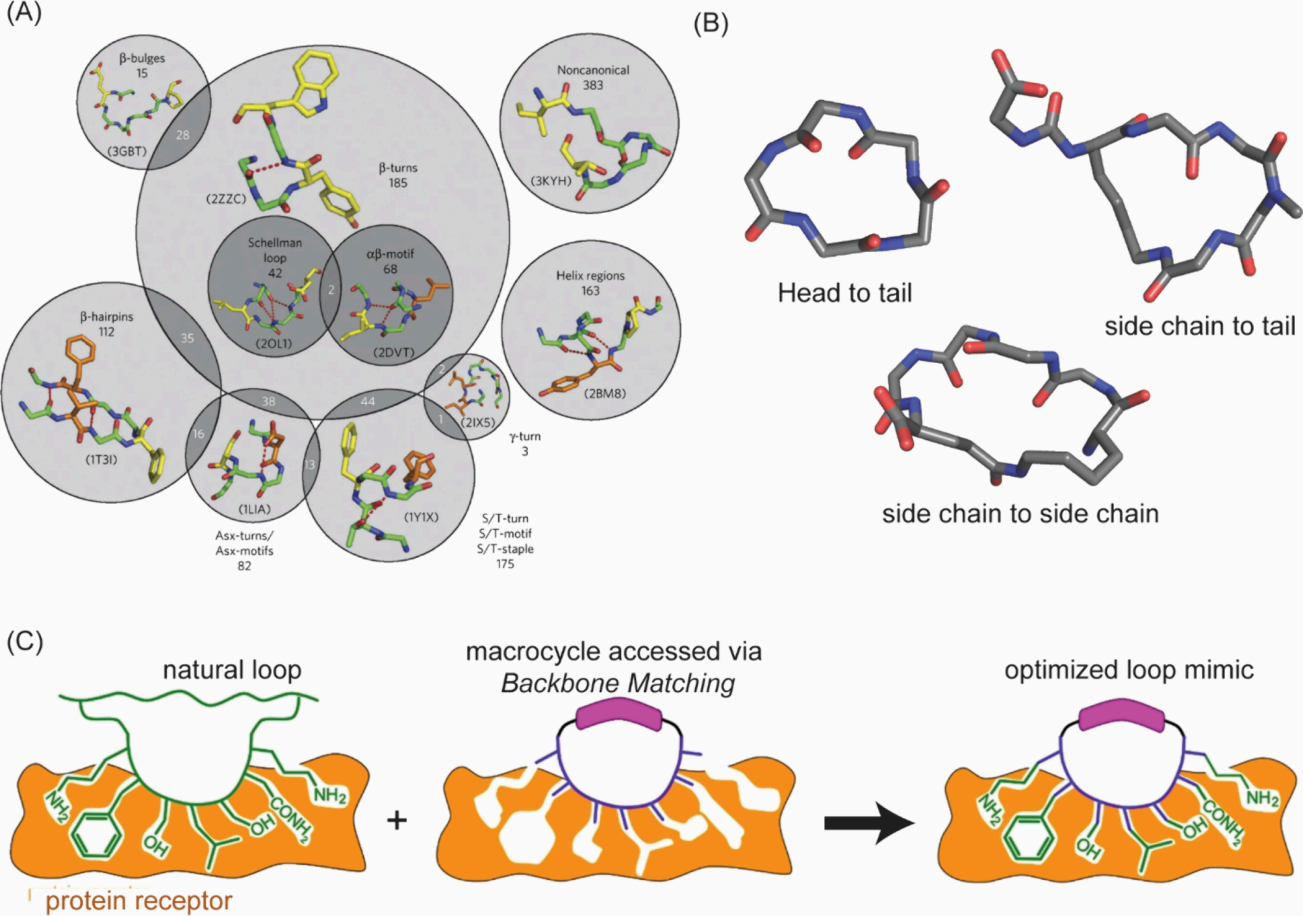
(A) Examples of loops found at protein interfaces, as analyzed by Kritzer et al., where the loop residues make important contributions to protein complex formation. This figure is adapted from ref 319. (B) Peptide macrocycles that mimic loops can be accessed via range of chemistries. (C) Burgess et al. have introduced a virtual tool (“Backbone Matching”) to identify macrocycle scaffolds that match loop geometries in bound receptors.

Loops naturally lend themselves to mimicry as peptide macrocycles. One can envision head-to-tail, side chain-to-tail, or side chain-to-side chain macrocycles (Figure 25B) or a via range of other chemistries.^166^ Natural products, including vancomycin, microcystin and cyclosporin, have continued to inspire for discovery of new macrocycles.^322^ Peptide macrocycles constitute a powerful class of potential therapeutics because they resist proteolytic degradation compared to their linear peptide counterparts. Macrocycles are often also more passively cell permeable than linear peptides.^323–325^ The recent demonstration by scientists at Merck that large peptide macrocycles can be designed to be orally bioavailable has further raised excitement in the field.^326,327^

Macrocyclization is the most common method for locking peptide conformations and several examples of natural and synthetic peptides in complex with proteins are known.^328^ Fairlie et al. analyzed crystal structures for 211 peptide macrocycles in complex with 65 different proteins to decipher binding modes for backbone conformation, and backbone and side chain contacts with protein targets. These authors find that unlike small molecules, cyclic peptide binding with targets is not driven primarily by hydrophobic contacts and that polar and hydrogen bonding interactions are critical components of macrocycle binding.^328^

Burgess and co-workers have focused on systematic design of loop mimicking peptide macrocycles (Figure 25C).^329^ This group recently described their approach to predict cyclo-organopeptides through a combination virtual screening and MD simulations with the goal of reducing a loop conformation in a bound structure to a synthetic macrocycle. In a complementary effort, the Roche group utilizes structures of hypervariable loops, which are critical antibody elements for antigens recognition, to generate hairpins.^330^ The rational design of synthetic loops is complemented by high-throughput screens, which we will discuss in the next section.

### 3.4 Proteomimetics and Miniproteins

The above discussion highlights the role of protein secondary structure mimics as attractive starting points for inhibition of challenging protein–protein interactions. Individual secondary structures are critical elements of protein interfaces; however, many protein–protein interfaces feature more complex modes of binding, and single secondary structures often do not offer sufficient binding epitopes for specific recognition. In this section, we discuss the role of tertiary structure mimetics^331,332^ or miniproteins^333,334^ as attractive candidates for the design of complex epitopes.

Proteomimetics are synthetic scaffolds that seek to mimic the complex topology of proteins beyond secondary structure mimics.^335^ Three examples that illustrate the goal of proteomimetics as a design principle are illustrated in Figure 26. The NF-κB essential modulator (NEMO or IKKγ) serves as a key fulcrum in the NF-κB pathway by relaying upstream signals to the IKK complex catalytic subunits through its elongated coiled coil motif.^336^ NEMO is hijacked by various external factors, including viral oncoproteins^337–339^ to initiate aberrant signaling. The example in Figure 26A illustrates the complex between vFLIP, an oncoprotein from Kaposi’s sarcoma herpesvirus (KSHV). Hotspot residues that mediate the interaction between vFLIP and NEMO are distributed over two helices. Screening with small molecule libraries and α-helical secondary structure mimics of NEMO thus failed to inhibit NEMO–vFLIP complex formation. We have shown that a proteomimetic that captures the two helical domains was required to inhibit the interaction.^212^

**Figure 26:**
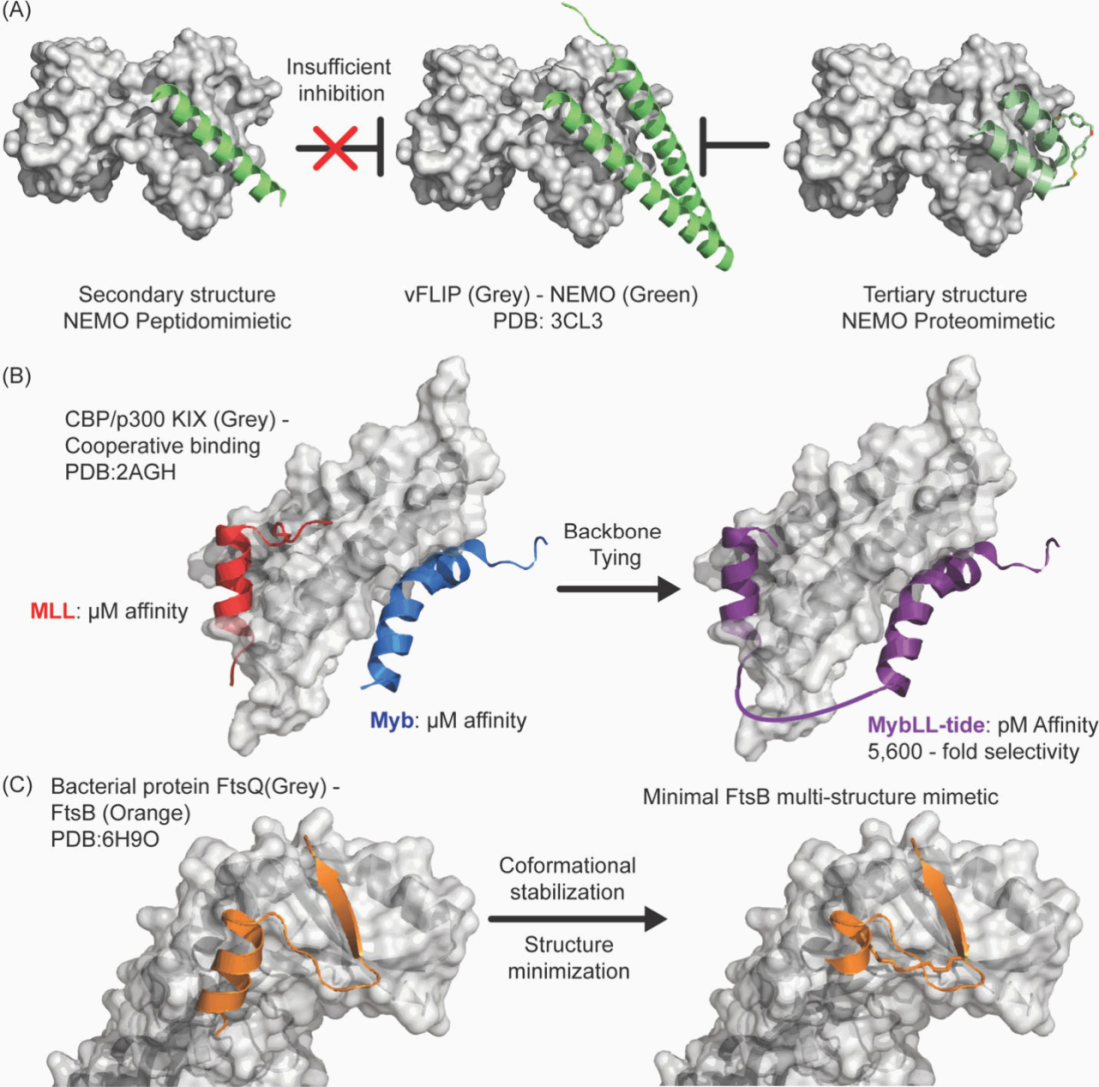
Three examples of proteomimetics, which encompass multiple secondary structures. (A) The interaction of NEMO and vFLIP is implicated in Kaposi’s sarcoma. NEMO utilizes a coiled coil domain to engage vFLIP. Single helix mimics and small molecule libraries failed to inhibit this PPI but a helix dimer mimic showed potent inhibition of the complex *in vivo*. (B) Transcription factors MLL and Myb bind different surfaces of coactivator KIX with micromolar affinity. In MybLL-tide, the two helical domains are conjugated to access a high affinity proteomimetic. (C) The interaction of FtsB and FtsQ represents an antibacterial target. FtsB uses contact residues from an extended region to contact the partner. Minimization and cyclization of the proteomimetic, that consists of a helix and strand spanned by a disordered region, leads to a potent lead.

Another key advantage of a proteomimetic is that it can make more contacts with the target than a peptidomimetic. MybLL-tide nicely illustrates this critical benefit of a proteomimetic (Figure 26B). MybLL-tide encompasses two helical binding partners of the KIX coactivator, MLL and Myb. Transcription factors Myb and MLL bind KIX weakly and on different faces of the coactivator. The ternary complex between these transcription factors and the coactivator serves as a model for exploring mechanisms of allostery and disorder in weak protein–protein interactions.^340^ Mapp and co-workers showed that by virtue of its bivalent nature, MybLL-tide binds KIX with exquisite affinity and specificity, resulting in the most potent synthetic reported ligand for this challenging PPI target.^181^ Proteomimetics that encompass secondary structures beyond helical domains have also been described. Grossman and co-workers have described a covalent proteomimetic that encompasses α-helical and β-strand regions.^341^ These co-workers minimized and macrocyclized a region of FtsB to develop inhibitors of FTsB complex formation with FtsQ. The association of FTsB and FtsQ is implicated in Gram-negative bacterial cell division. In this work, the authors captured the complex epitope of FTsB in a synthetic ligand and showed that compound can serve as a model for a new class of antibiotics.

Proteomimetics are synthetic tertiary structure mimics that aim to build on the success of miniproteins and antibodies as complex binding epitopes. Antibodies have proven to be a successful class of therapeutics, with over 100 derivatives now in the clinic.^342^ However, antibodies suffer from poor tissue penetration, high production cost and are largely ineffective against intracellular targets. Engineered antibody fragments and small proteins present an attractive alternative to antibodies.^24^ Miniproteins are defined as folded protein scaffolds below 10 kDa size. Several classes of miniproteins that can engage their biomolecular targets with high affinity and specificity have been described.^343–345^ Roughly 20 engineered proteins have been reported to date^343–345^ and many of these scaffolds can be screened using phage display. Figure 27 captures the diversity of epitopes displayed by miniproteins and synthetic proteomimetics.

**Figure 27:**
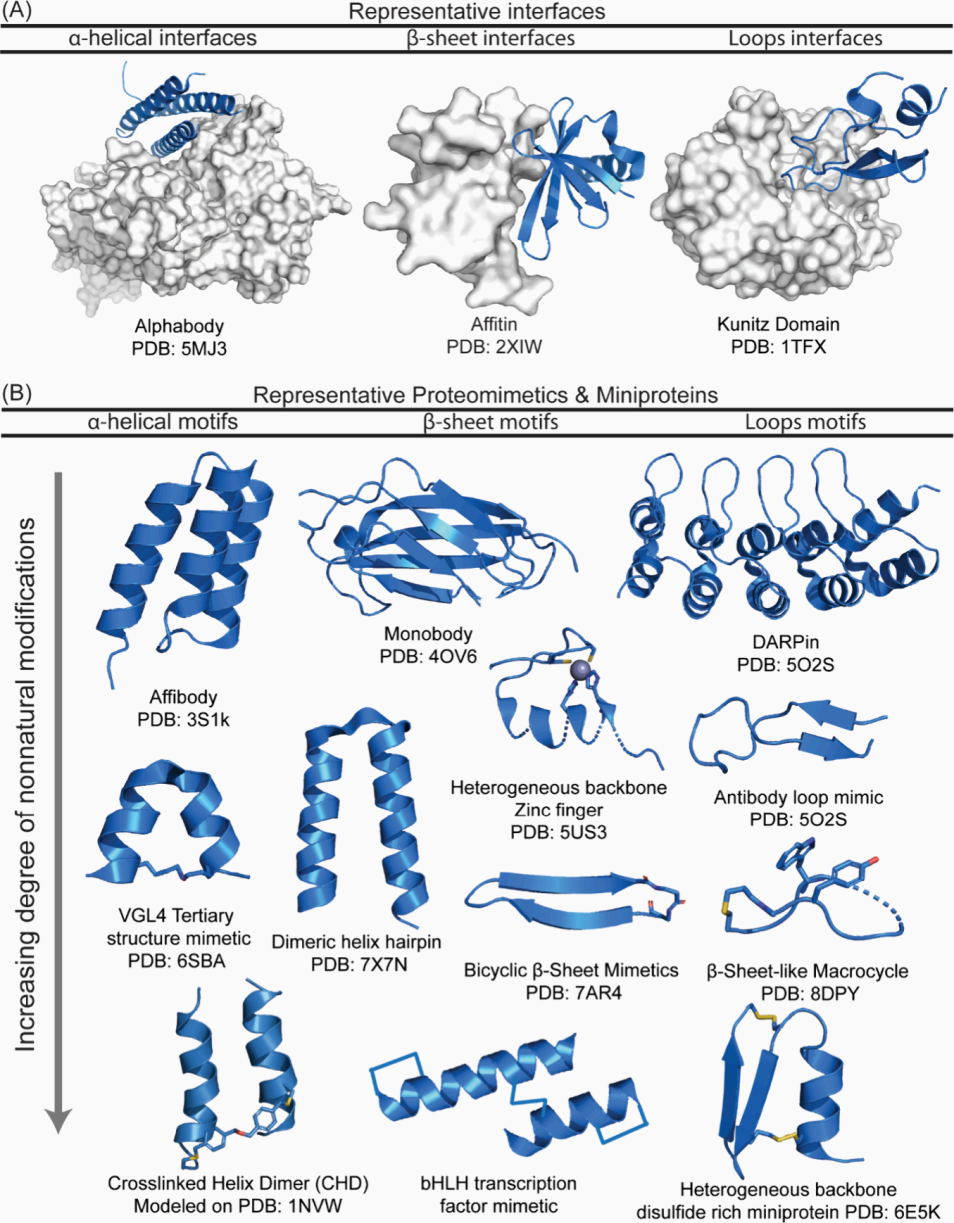
Diversity in miniproteins and synthetic proteomimetics.

Proteomimetics and miniproteins are typically highly specific reagents because they can utilize a large set of contacts to engage the target; however, this large size often results in compounds that exhibit poor cellular uptake. A range of strategies for enhancing cellular uptake of these large molecules are being explored and are discussed in Section 6.2 .^346–349^ Understanding sequence-based protein folding has provided a strong foundation for the design of proteomimetics and miniproteins. However, the structural diversity of such platforms has predominantly been biased toward helical conformations. Consequently, efforts to design higher-order protein domain mimics with more complex geometries are increasingly appreciated and pursued.

## 4 Screening Strategies for PPI Inhibitor Discovery

An early goal in the field of chemical biology focused on *chemical genetics* and the establishment of a systematic approach to explore biology with small molecules.^350,351^ This lofty goal of identifying a small molecule ligand for any protein inspired efforts to create large libraries of compounds and screen these libraries for hits.^352^ Screening efforts could be categorized as (A) phenotypic screening or (B) target-based screening (Figure 28).^353,354^ In phenotypic screens, also referred to as *“forward chemical genetics”*, the goal is to find a hit from a collection of compounds that leads to a desired and specific biological result such as inhibition of mitosis, modulation of transcription of a particular gene, or inhibition of specific kinase signaling.^355^ Phenotypic screens are often performed with libraries of drug-like molecules, and compounds that emerge from these screens become attractive leads for drug discovery.^354^ A key benefit of phenotypic screens is that it provides impetus for finding new targets that drive the desired biological activity.^355^ Several compounds that gave the field of chemical genetics its initial appeal have been discovered through phenotypic screens. Monastrol, an inhibitor of mitotic spindle formation, was found in a small molecule library during a search for compounds that induced changes in spindle formation without perturbing tubulin polymerization.^356,357^ Discovery of monastrol also led to the discovery of its target, motor protein Eg5, establishing the elegance and potential of forward chemical genetics.^357^ Similarly, the anticancer drug lenalidomide, which has been approved by the USA FDA, was discovered from a phenotypic screen, but the elucidation of its target, an E3 ligase protein cereblon, did not occur until years after its approval in 2012.^354,358^ Not surprisingly, target identification and determination of mechanism of action of advanced lead compounds remain significant bottlenecks - but are being aided by the revolution in chemical proteomics.^353,359–361^

**Figure 28:**
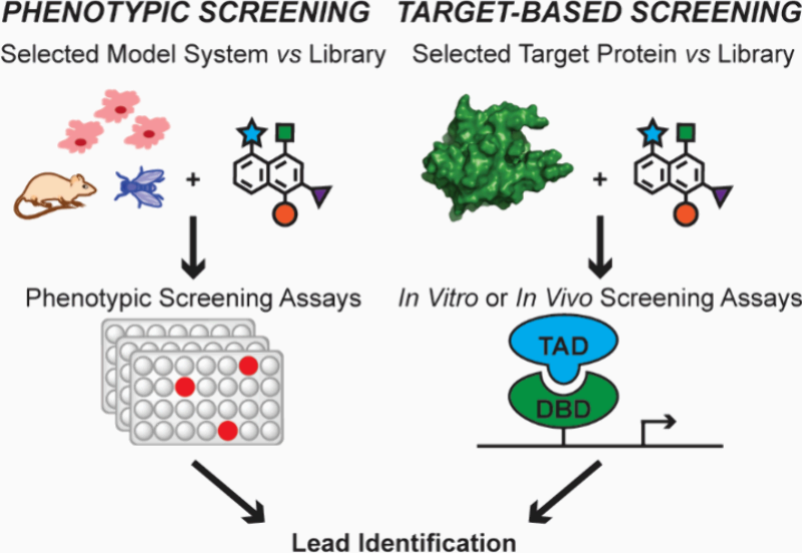
Leads have been isolated from compound libraries using phenotypic and target-based screens. In phenotypic screening, a compound library is screened in a model system (i.e., cells, mice, flies) and analyzed for a specific phenotype. Target-based screens utilize a particular protein target of interest in cell free or cell culture assays.

In target-based drug screening, also referred to as *“reverse chemical genetics,”* specific compounds are screened to modulate a particular target or protein of interest.^355^ This approach requires a biologically validated target or pathway; however, a high-resolution structure of the target is not needed. Target-based drug discovery has gained prominence with growing understanding of cellular networks and molecular targets from genome sequencing.^354,362^ Several methods, including ELISA-based screens, split luciferase, and yeast two-hybrid assays, are widely used to screen compounds against a desired protein of interest both *in vitro* and *in vivo*.^363^ These approaches do not require an intimate knowledge of the molecular details of targeted protein interfaces. Nutlins, which are small molecule ligands of MDM2 and potent inhibitors of the p53/MDM2 interaction (Figure 9D), were discovered from a target-based high-throughput screen.^89,364^ While high-throughput screening has become relatively low cost and efficient, replication of the protein–protein interaction within the assay often remains problematic. For example, only part of the protein target may be able to be expressed and amenable to an assay format, or multiprotein complexes and other cofactors play a more substantial role *in vivo* as compared to what is replicated in assays.^365^ Another general challenge of PPI targeting screening approaches is that often the compound libraries are not structurally diverse enough to target large and diffuse interfaces.^366^ To address this challenge, several groups are developing strategies for the synthesis of complex natural product-like libraries.^367–369^

Below we focus on two types of high throughput screening strategies that have been critical for the classical and continuing advances for PPI inhibitor discovery: (i) fragment-based libraries and (ii) genetically encoded libraries.

### 4.1 Fragment-Based Screening

Successful PPI modulators are generally larger than traditional drugs, typically double or triple the molecular weight range preferred for enzyme inhibitors.^370^ Drug-like libraries, developed for traditional drug targets, often lack the characteristics needed to engage a protein’s surface.^370,371^ Thus, screening of drug-like compound libraries against PPI surfaces often leads to nonspecific and low affinity hits. To address these limitations, fragment-based screening techniques have been developed.^371,372^ These techniques envision that assemblies of multiple drug-like molecules or fragments stitched together could offer high affinity ligands for flat protein surfaces (Figure 29).

**Figure 29:**
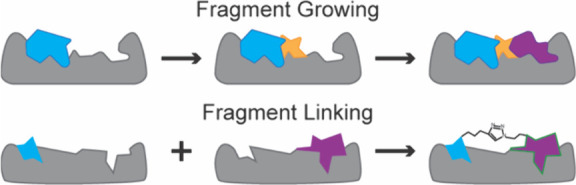
Fragment-based design. (Top) Fragment growing. A single fragment is progressively grown to optimize contacts with the target protein. (Bottom) Fragment linking. Multiple fragments that bind in nearby sites are individually optimized and subsequently linked together.

Pioneering work by researchers at Abbvie, utilized fragment screening as a primary method for the discovery of ligands that bind BCL2 - an effort that eventually led to venetoclax.^8^ This fragment-based drug discovery efforts utilized 2D NMR as tool to identify binding sites. In this method, 2D NMR identifies fragments that produce chemical shifts that perturb the protein structure, hence the name “SAR by NMR.” As with other fragment-based methods, low affinity (μM-mM) fragments, when linked together, produce high affinity compounds (nM).^92,373,374^

#### 4.1.1 Protein Tethering

In protein tethering, or site-directed ligand discovery, an engineered or native cysteine residue is employed to form covalent linkage with fragments from a library and guide individual fragments into a neighboring protein pocket (Figure 30).^15,375^ The standard fragment screening methods require high fragment concentrations (1 mM) because the binding affinity of any fragment for the target is weak. The use of high fragment concentrations that can lead to false positives due to aggregation.^376,377^ Tethering increases the local concentration of the fragment, allowing for screening of fragments at lower concentrations than if they were not tethered. Site-directed fragment placement is another critical advantage of protein tethering over nontethered fragment screening—the potential binding of a fragment can be tested at a defined site.

**Figure 30:**
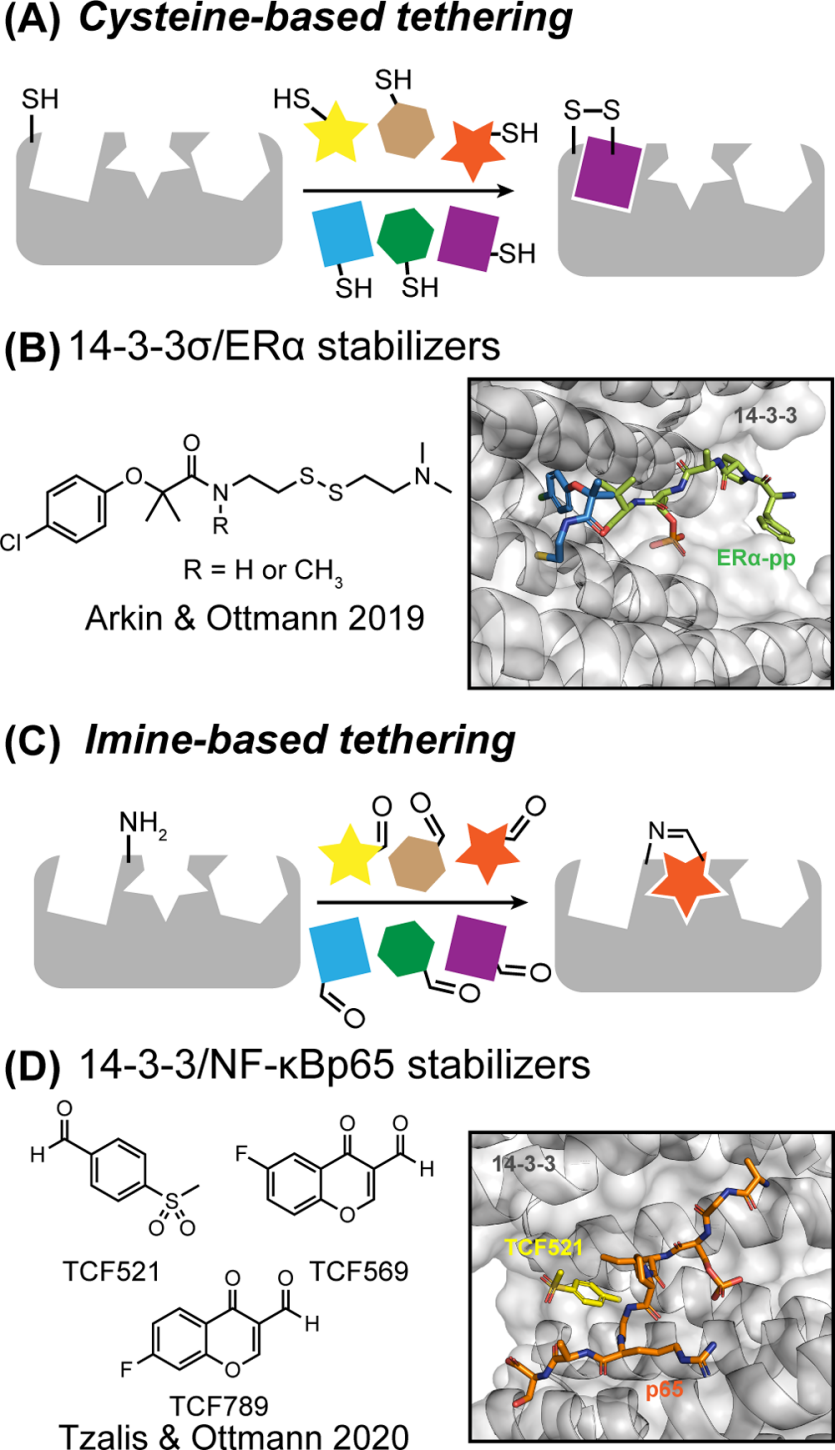
Protein tethering utilizes reversible covalent bonds between the fragment library and the cysteine or lysine-modified protein (A and C) to obtain disulfide or imine linkage. Fragments that modify the protein are detected by mass spectrometry. Protein tethering has been applied to discover stabilizers of 14-3-3 protein–protein interactions (B and D).

In tethering, disulfide-containing screen fragments undergo thiol–disulfide exchange forming reversible covalent bonds with native or engineered cysteines in the target protein (Figure 30A). Traditional tethering approaches are subsequently identified using mass spectrometry (MS-based screen) to detect disulfide bond formation. In a recent effort, this approach was utilized to screen for stabilizers of the 14-3-3σ/ERα PPI at the fusicoccin A binding site. 14-3-3σ is a member of the 14-3-3 protein family that plays a crucial role in many biological processes and pathologies. Stabilizing 14-3-3σ/ERα PPI was proposed to be a valid alternative strategy for interfering with ERα-positive breast cancer. In a study by Sijbesma et al., a site-directed screening of 1,600 disulfide fragments was performed against three 14-3-3σ constructs (Cys38, Cys42, and Cys45), leading to the identification of orthosteric stabilizers that enhanced 14-3-3σ/ERα affinity by up to 40-fold.^378^ Notably, X-ray crystal structures of selected fragments bound to 14-3-3σ­(C42)/ERα revealed that the tethered fragment acts as a molecular glue, bridging the two partner proteins (Figure 30B).

Imine-based tethering have also been successfully applied to identifying fragment-based stabilizers of PPIs (Figure 30C). By forming reversible imine bonds with lysine residues to form aldimines, this approach allows for the discovery of novel stabilizers that can enhance or disrupt specific PPIs, expanding the toolkit for drug discovery and enabling the targeting of previously undruggable proteins. Wolter et al. were the first to developed an imine-based tethering via aldehyde fragment screening for the modulation of the 14-3-3/NF-κβ PPI (Figure 30D).^379^ Screening efforts with aldehyde libraries identified aryl aldehydes capable of forming aldimine with Lys122 at the PPI interface of the p65-subunit-derived peptide of NF-κB with 14-3-3 protein. Structural data from cocrystallization of the lead compound, TCF521, with 14-3-3/p65 complex revealed that the sulfonamide group of TCF521 plays a key role in activating the aldehyde moiety, while the benzyl ring provides hydrophobic interactions with Ile46 of p65 (Figure 30D). For further insights into the applications of cysteine- or imine-based tethering in PPI targeting, Lucero et al. recently published a comprehensive review detailing the latest developments and applications in site-directed fragment tethering approaches.^380^

Protein tethering typically utilizes a mass spectrometry assay to determine fragments that covalently link the target protein (Figure 31A). Mapp et al. reported a fluorescence polarization-based method to expand the scope of protein tethering and discover high affinity ligands difficult targets (Figure 31B).^381,382^ This method offers a significant advantage over traditional liquid chromatography–mass spectrometry (LC-MS), as it provides a more rapid and accessible way to monitor binding events. Applying this FP-based tethering screen, the researchers successfully identified inhibitors of the KIX CBP/MLL interaction, replicating the same set of ligands previously discovered through the MS-based screen. Tethering fragments via disulfide bonds can increase their binding affinity by 10 to 100-fold into ranges readily detectable by FP making it an effective tool for high-throughput screening of PPI modulators. Similarly, they applied FP-based tethering screens to identify ligands that disrupt the more challenging surface within KIX CBP, the interaction with pKID. This screen identified 63 unique fragments, 9 of which were confirmed to displace pKID from the KIX domain, with results validated through mass spectrometry. Overall, FP tethering offers a powerful, complementary strategy for detecting and confirming potent PPI modulators in high-throughput screening settings.

**Figure 31:**
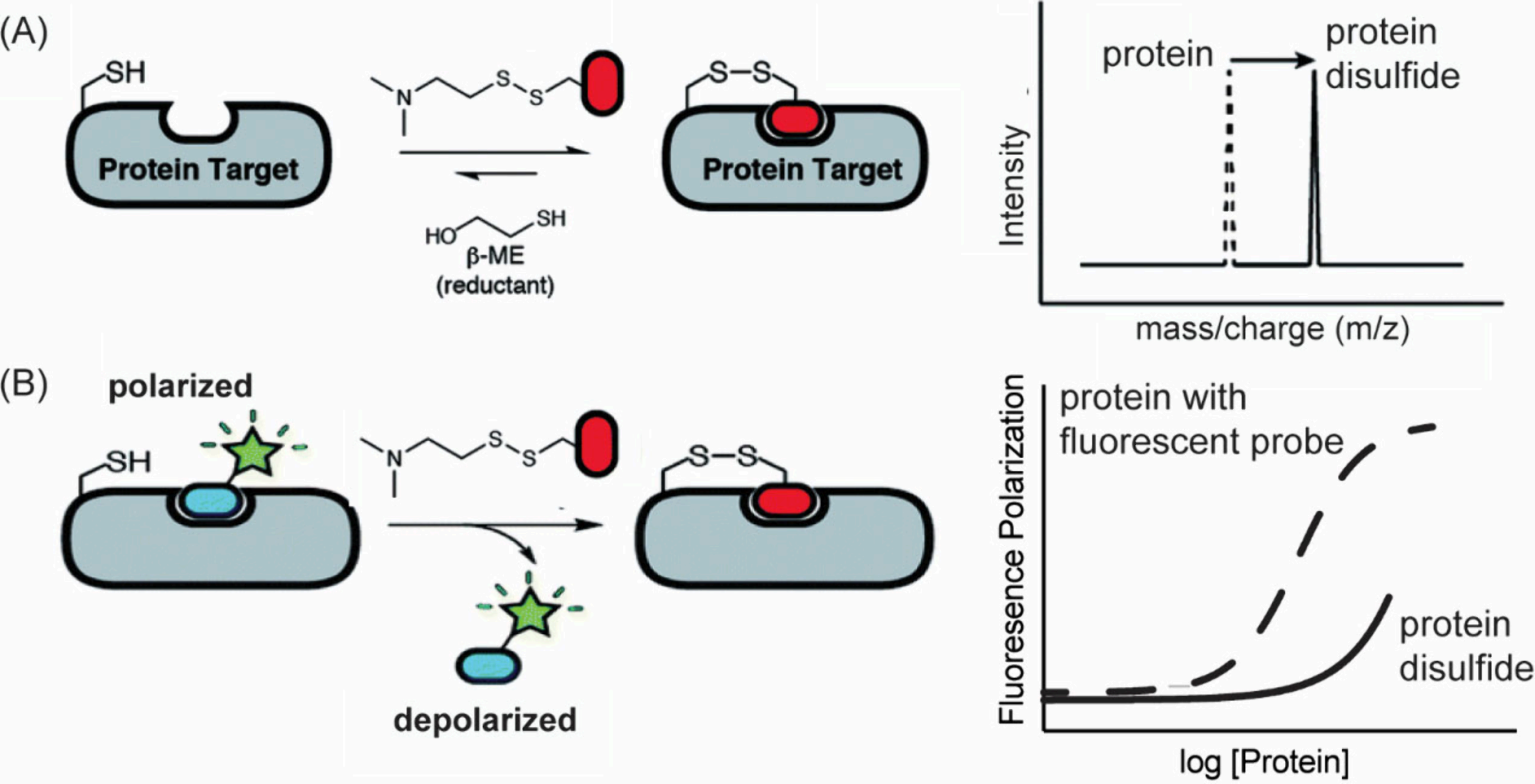
(A) Protein tethering typically utilizes a liquid chromatography-mass spectrometry (LC-MS) assay. (B) The scope of tethering has been expanded to employ fluorescence polarization (FP) assay for direct detection of protein modification by a fragment.

#### 4.1.2 Peptide Tethering

Protein tethering is built on the idea that proximity-induced fragment screen can identify fragments for cryptic pockets. Building on this idea, our group recently introduced a fragment design approach applied to a conformationally defined peptide scaffold. The method, termed peptide tethering,^383^ envisions side chains on a peptide to be fragments that can be experimentally screened. Peptide tethering builds on a combination of the protein-tethering^15,16^ and fragment linking approaches,^384^ and involves judicious placement of a reactive group on a stabilized designed peptide- or proteomimetic as opposed to the protein itself (Figure 32). The fragments are covalently linked to a peptide containing a fluorophore and the relative impact of fragments on binding can be quantified using a fluorescence polarization assay. For this strategy to succeed, residues to be screened must be directed into nearby pockets, i.e. the peptide must retain several native critical binding residues and native orientation to direct new fragments into the desired sites. For example, in Figure 32B,C, the green hexagon would be expected to anchor the peptide into the correct position on the receptor and allow side chain fragment screening at the chosen site.^383^ This approach identified a peptidomimetic that binds the KIX CBP with >2000-fold improved affinity as compared to the wild-type sequence, resulting in HBS-II as a submicromolar inhibitor of KIX/MLL complex formation (Figure 32D). In our previous studies, we used the AlphaSpace computational software to topographically map protein surfaces and design natural and nonnatural side chains on peptide mimics.^385^ This study highlights the effectiveness of an integrated computational–experimental approach as a versatile framework for optimizing peptidomimetics to inhibit protein–protein interactions.

**Figure 32:**
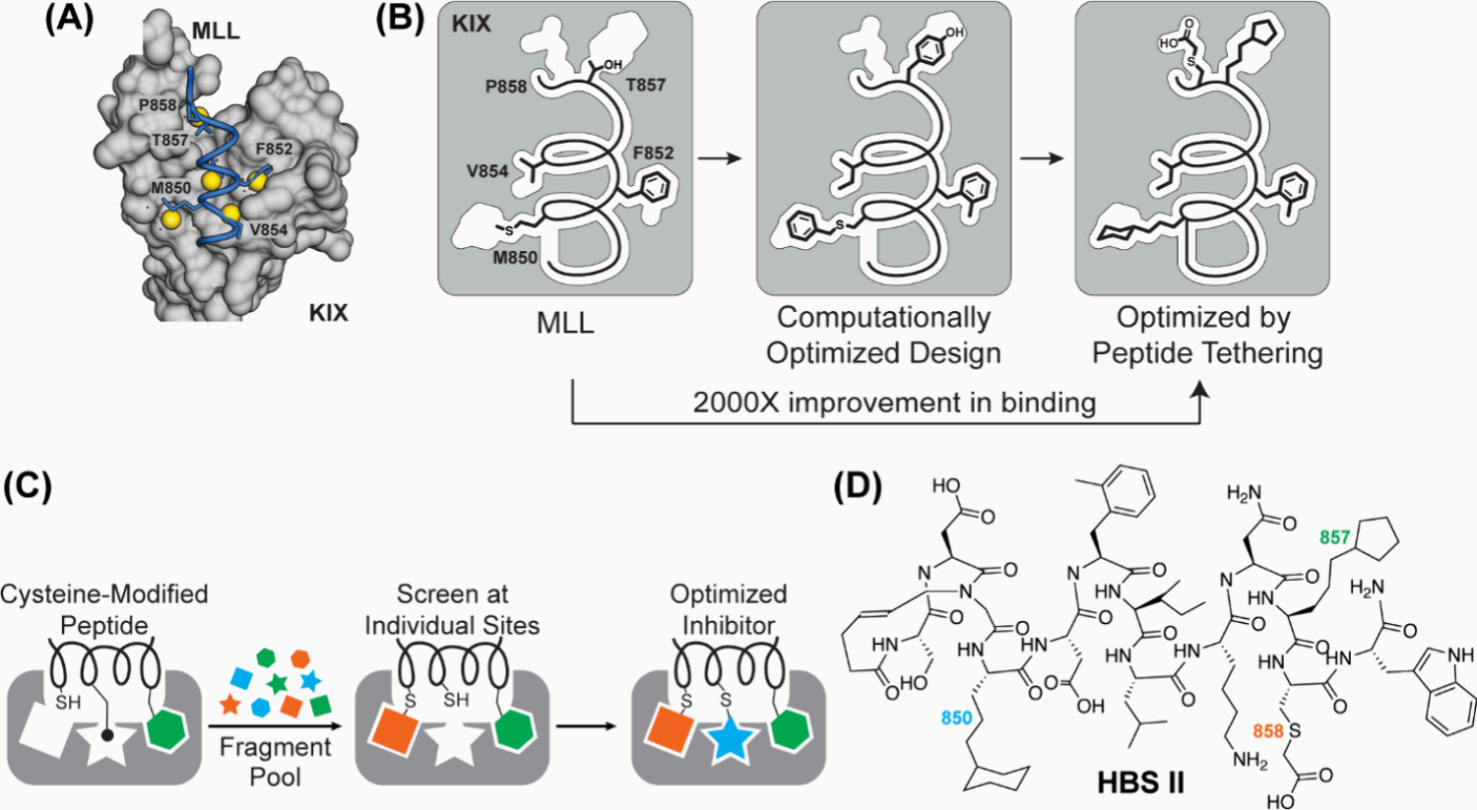
KIX domain of coactivators p300/CBP interacts with a multitude of transcription factors. (A) An NMR-derived model of KIX in complex with MLL and cMyb is shown (PDB: 2AGH). The helical domain of MLL provides a template for the development of synthetic ligands for KIX. The yellow spheres in (A) depict the centroid of potential pockets near the MLL helix. (B) Workflow described in this study: The topographical map of KIX suggests that several cryptic pockets on its surface are not optimally occupied by native MLL residues, and nonnatural residues may be designed to provide enhanced affinity. The computationally revealed cryptic pockets were accessed via a peptide-tethering enabled fragment screen resulted in HBSII that binds KIX with a 2000X improvement over the native MLL. (C) Peptide tethering is performed following computational optimization allowing fragments to be individually screened at the pocket level experimentally. (D) Fragment screen yielded optimized peptide HBS II. HBS II binds KIX with submicromolar affinity.

### 4.2 Genetically Encoded Libraries

Synthetic compound libraries have the advantage of directly selecting for drug like small molecules or macrocycles; however, the cost associated with the synthesis of the libraries and deconvolution remain a bottleneck. A key advantage of biological libraries is that the libraries can be evolved using biosynthetic machinery and hits can then be deconvoluted using DNA sequencing because each member of the library is encoded. Recent efforts have allowed merging of the respective advantages of the synthetic and biological libraries such that synthetic compounds can be linked to the genotype and hits readily identified by next-generation DNA sequencing. Below we discuss phage display, mRNA display, DNA-encoded libraries, and split-intein circular ligation of peptides and proteins (SICLOPPS) that have become powerful tools for the display of synthetic ligands, with a particular focus on macrocycles.^386^

#### 4.2.1 Phage Display

Phage display is one of the oldest and most robust *in vitro* selection techniques to generate peptide- and protein-based ligands for a given target with desired properties including enhanced affinity, specificity and stability.^387,388^ Filamentous bacteriophages, hereafter “phages,” have become attractive biological model systems for building combinatorial libraries. The developments and applications of several phage display systems, such as T7, T4, and λ, are well-documented and extensively summarized in several reviews.^389–392^ In phage, the displayed elements (peptides and proteins) are expressed together with coat proteins and displayed on the outside of phage (Figure 33). The encoding genetic material of each display element is encapsulated in single-stranded DNA (ssDNA) within the phage particle, allowing for identification through sequencing of the phage DNA. This genotype-phenotype linkage enables libraries of billion peptide or protein variants to be screened against a target in a matter of days.

**Figure 33:**
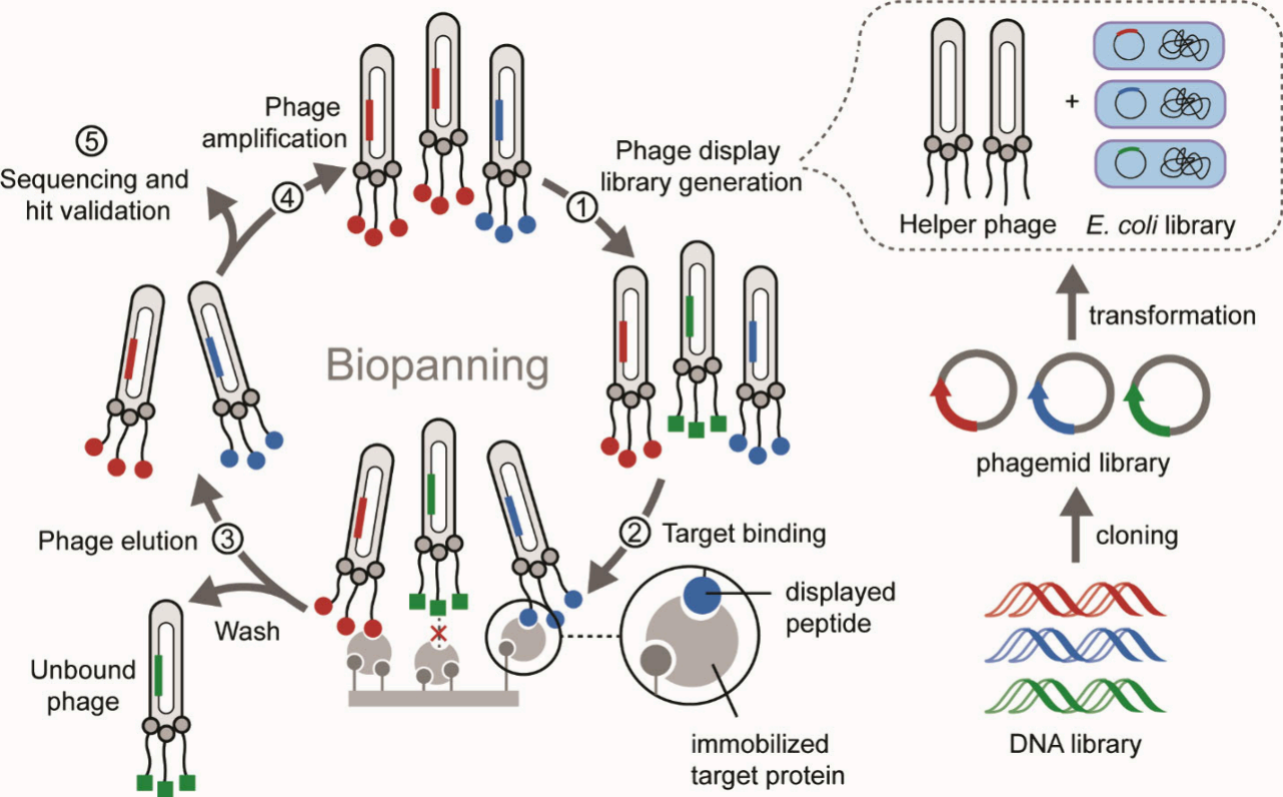
A general workflow for phage display selections. A DNA library encoding the peptide of interest is coexpressed with the phage coat protein. The encoded phage library is subjected to affinity selection, a process called biopanning, to enrich peptide ligands. Typically multiple rounds of biopanning are required to enrich the top hits. The enriched phage DNA is sequenced to identify the peptide.

In phage display, libraries are screened *in vitro* using an affinity-based selection process known as biopanning. The affinity selection process has four main steps: 1) immobilization of the protein target on a solid support, 2) coincubation of phage population with the protein target, 3) stringent washes to remove nonspecific or weak binding phages, and 4) bound phages are subsequently eluted and amplified in an *E. coli* host (Figure 33).^393,394^ The entire biopanning process can be repeated 3–5 times to progressively enrich for phages displaying high-affinity binders and selection stringency can be incremented in each round with additional washes or reducing immobilized protein concentration.

Peptides with specific binding affinities predominate the phage population after several rounds of selection cycles. To identify these positive clones, DNA sequencing is employed to determine the selection stage and to observe consensus profiles of phage-selected peptides. Traditional DNA sequencing method like Sanger sequencing requires 20 or more unique clones to create a high-resolution profile, often involves random selection of individual phage clones. However, over the past two decades, more powerful sequencing techniques like next-generation sequencing (NGS) has revolutionized this process at a relatively low cost in a short time frame.^395–397^ Analyses of selected phage population from NGS typically include the number of unique sequence counts, a good indicator for library size, and the frequency distribution of individual sequences. By integrating NGS with phage display, researchers have obtained a comprehensive assessment of library diversity, ensuring that even the most subtle variations are well-represented and analyzed.

Early phage display methods focused on enrichment of high-affinity proteins or linear peptide binders. Recent advances have introduced chemical modifications on to the phage displayed ligands to generate libraries of macrocyclic peptides or conformationally defined epitopes. In the following section, we will highlight key advancements in post-translational chemical modifications on phage-displayed peptides to generate ligands for protein surfaces.

Efficient methodologies now exist to increase conformational rigidity of phage-displayed peptides including synthesis of multicyclic peptides exhibiting enhanced conformational and proteolytic stabilities and binding affinities. In most cases, strategies for phage-displayed peptide modifications rely on natural side chain cross-linking and N-terminal amine chemistry. Cysteines are among the most common reactive handles for attaching small organic chemical reagents to displayed peptides. They have been widely employed in generating multicyclic and constrained peptides in various applications. A classical study by Heinis et al. demonstrated cysteine alkylation using 1,3,5-tris­(bromomethyl)­benzene (TBMB) to modify a phage-displayed linear peptide with three cysteines (CX_*n*_CX_*n*_C, where X is randomized and *n* = 3–6 residues) (Figure 34).^398^ This trivalent thiol-reactive compound constrains peptides into bicyclic structures via thioether linkage generating peptides with exceptional proteolytic resistance and high binding affinity and specificity. Cysteine-mediated chemical modifications have since been extensively expanded with alternative cross-linkers in both phage and mRNA display libraries, for more information on biocompatible peptide macrocyclization, we guide our readers to some recent excellent reviews.^399,400^ Figure 34 provides an overview of the key chemical reactions performed on phage-displayed peptides using natural amino acids as reactive handles.

**Figure 34:**
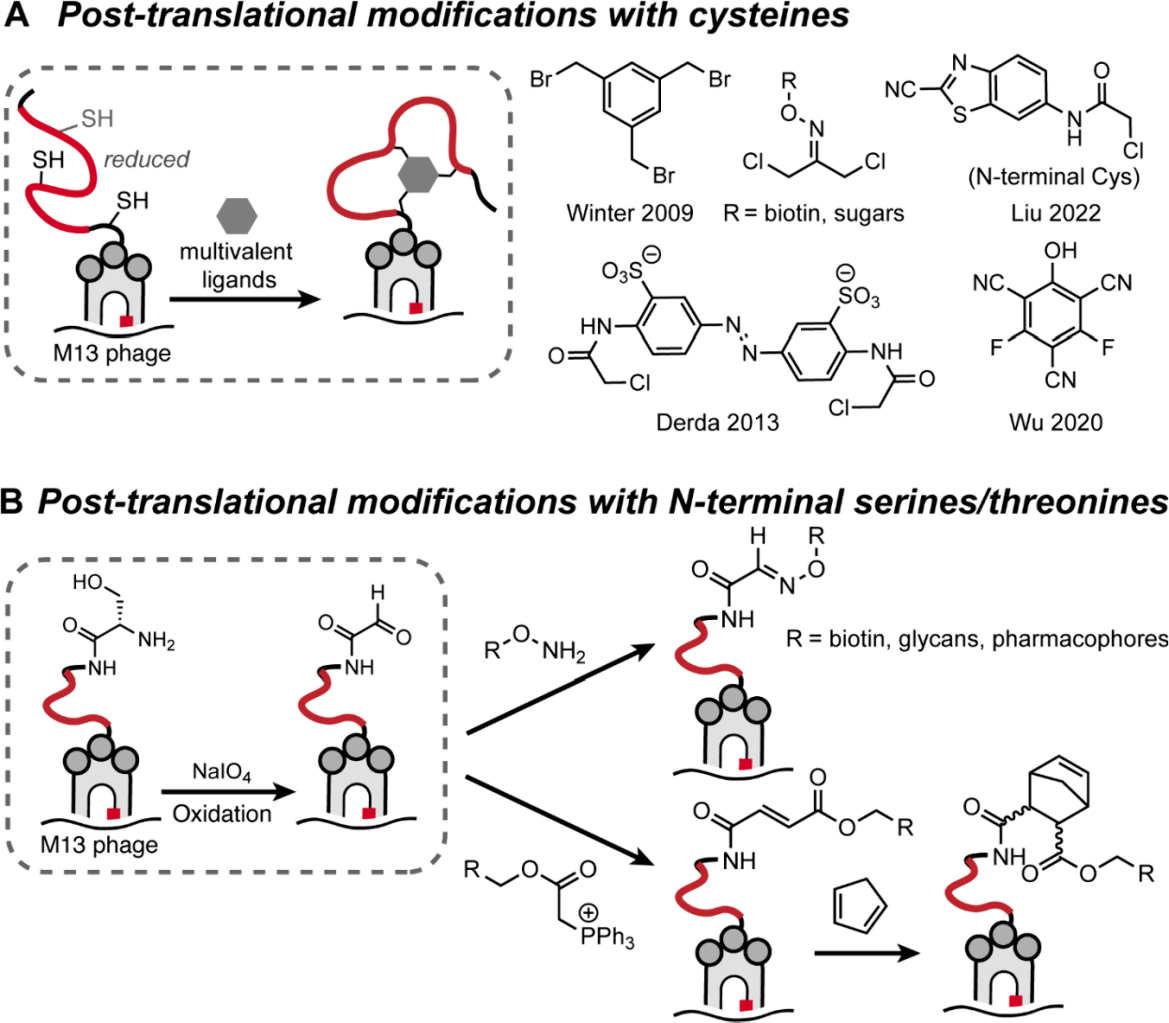
Post-translational modification phage displayed peptides by exogenous reagents. (A) Phage peptides containing cysteine residues can be modified with a range of electrophiles. (B) Oxidation of N-terminal serine leads to an aldehyde group that can also reacted with oximes as bioconjugation handles or with Wittig reagents to yield a dienophile for subsequent Diels–Alder reactions.

Cysteine-based cyclization on phages can pose two major challenges: the presence of native cysteines within the phage and the introduction of cysteines at randomized positions. To address these concerns, Schmid and colleagues developed a cysteine-free phage variant through directed evolution of the coat protein. This approach effectively eliminated native cysteines while maintaining proper coat protein folding.^401^ Additionally, to enhance the specificity and efficiency of thiol-reactive cyclization, it is essential to avoid arranging cysteines at randomized positions created by degenerate codons in the library design. Overall, cysteine residues have been a workhorse for modifying phage-displayed peptides. Beyond alkylation, other powerful chemistries such as Michael addition to maleimides,^402^ oxidative elimination to dehydroalanine,^403^ and native chemical ligation with N-terminal Cys^404^ have also been reported for use in phage display applications.

Another popular strategy for diversifying post-translationally modified peptides on a phage is enabled by N-terminal serines and threonines. These residues can be readily oxidized to highly reactive aldehydes with sodium periodate (NaIO_4_) and undergo various chemical reactions (Figure 34B). The Derda group pioneered this chemistry to install a different number of handles, including glycan moiety, biotin and sulfonamides via oxime ligation onto phage display.^405,406^ Kitov et al. expanded the scope of bio-orthogonal aldehyde chemistry on the M13 phage-display library by introducing aniline-containing nucleophilic substituents, 2-amino benzamidoxime derivatives (ABAO), as nucleophilic catalysts to increase reaction rates (*k* = 40 M^–1^ s^–1^) and long-term stability over classical oxime/hydrazone bonds.^407^ The ABAO framework described proceeds through a modified Pictet–Spengler-like reaction yielding a hydrolytically stable intramolecular ring formation, allowing reaction between the biotin-ABAO derivative and phage containing N-terminal glyoxal group to reach completion in 1 h under mildly acidic pH. In a separate study, the Derda group also demonstrated that aldehydes generated from NaIO_4_ oxidation can undergo Wittig reaction with phosphonium ylides to form internal olefins on phage libraries (Figure 34B), which can subsequently act as dienophiles in Diels–Alder reaction with cyclopentadiene, further expanding the range of bio-orthogonal transformations accessible for phage display systems.^408^

##### 4.2.1.1 Applications of Phage-Derived Peptides in Targeting PPIs

Chemical modification of phage peptide libraries allows direct screening of constrained peptides. A recent study by Li et al. in developing a rapid screening method with phage display identified several α-helical constrained peptides, termed Helicons, that exhibit inhibitory activities against protein–protein interactions, enzymatic activities, conformational rearrangements and protein dimerization. The screening utilized a naïve 14-mer α-helical peptide library, which incorporated two cysteine residues at the *i*, *i*+7 positions for stapling with N,N′-(1,4-phenylene)­bis­(2-bromoacetamide), a phage-compatible cross-linker that locks peptides into a stable α-helical conformation (Figure 35). Upon panning phage-displayed Helicon libraries, they identified both natural and unknown α-helix binding sites on β-catenin, notably β-catenin/TCF4 and β-catenin/Axin PPIs, proving a promising platform for interrogating undruggable target proteins.

**Figure 35:**
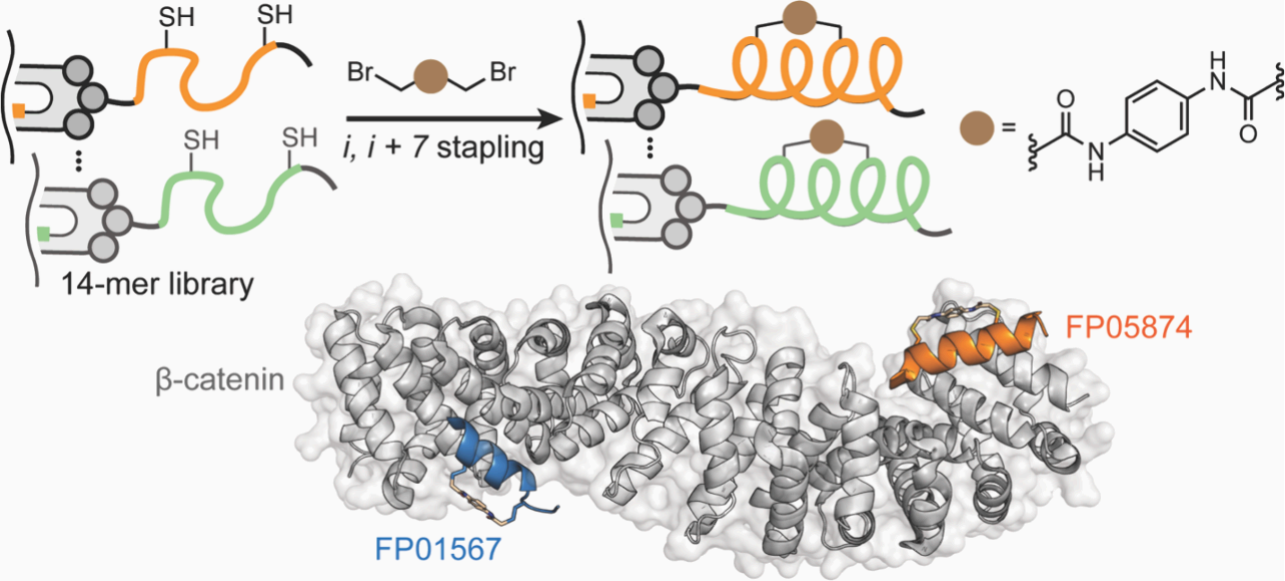
Cross-linking of a 14-mer peptide library with two cysteine residues at the *i*, *i*+7 positions with N,N′-(1,4-phenylene)­bis­(2-bromoacetamide leads to a library of phage displayed stabilized helices. Selection of this library against β-catenin affords two potent helical ligands, FP05874 and FP01567.

In contrast to chemically modify phage-displayed peptides, backbone stapling can also be performed through spontaneous cyclization with genetically encoded cysteine-reactive unnatural amino acids. In 2020, the Fasan group developed a platform termed macrocyclic organo-peptide hybrid phage-display system, or MOrPH-PHD, taking advantage of *O*-(2-bromoethyl)-l-tyrosine incorporated at UAG codons to form thioether bridged macrocycles with internal cysteines at the *i*, *i*+7 positions.^409^ Subsequent affinity selection against Keap1 and Sonic Hedgehog proteins identified binders with low micromolar to nanomolar affinities.

#### 4.2.2 mRNA Display

mRNA display is a powerful technology that enables the screening of trillions upon trillions of peptides against a protein of interest bound to a solid support. In this methodology, originally devised by Roberts and Szostak,^410^ a mRNA library is *in vitro* translated into the peptide. The critical aspect of the method is that each mRNA is covalently linked to its cognate peptide product through puromycin (Figure 36). The RNA-linked peptide library is screened against the target, the target-bound sequences are amplified by PCR and subjected to round 2 of the selection. After 5–8 rounds, the enriched peptides are identified by DNA sequencing. The *in vitro* nature of this display platform enables ease of incorporation of unnatural amino acids (UAAs), with high sequence diversity on the order of 10^13^ members.^411^ Macrocyclization of peptides, through the implementation of an electrophile as the UAA, has been a highly successful undertaking in mRNA display screens.

**Figure 36:**
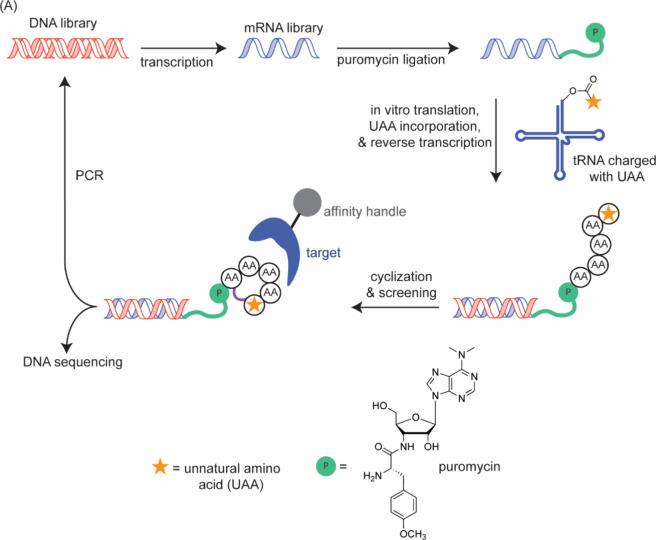
A general workflow for mRNA display selections. Each peptide in mRNA display library is covalently linked to its corresponding mRNA via a puromycin linker. The process involves *in vitro* transcription to generate the mRNA library, followed by translation to produce peptides, which are then subjected to affinity selection against a target of interest. After selection, the peptides that bind the target are identified by DNA sequencing of the corresponding mRNA sequences.

The inclusion of nonnatural residues in mRNA display can be achieved through flexizyme-mediated aminoacylations of tRNAs or synthesis of tRNAs charged with modified amino acids. Flexizymes^412^ are catalytic RNAs that were selected using SELEX for their ability to catalyze acyl-transfer reactions onto tRNAs.^413–415^ Charged tRNAs with UAAs allow insertion of a range of nonnatural residues into the peptide chain. Flexizyme UAAs that incorporate N-methyl,^416,417^ D-,^418^ β-,^419^ and γ-amino acids^420^ have been demonstrated (Figure 37A). mRNA display screens with thioether cyclization, involving an N-terminal α-halogenated amino acid that spontaneously cyclizes with a downstream cysteine, are commonly utilized (Figure 37B).

**Figure 37:**
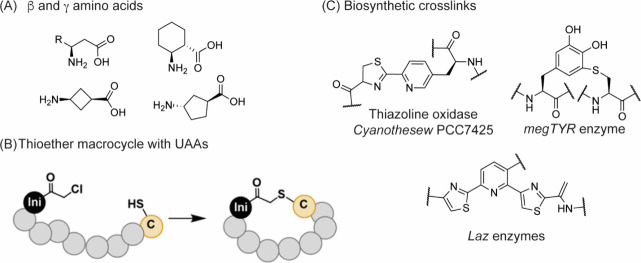
A critical advantages of mRNA display is that Flexizymes enable incorporation of a wide range of unnatural amino acid residues, including β- and γ-amino acids (A). (B) Macrocyclic peptides are easily accessed by thioether bridges (via reaction of cysteine thiols and acetylhalides). (C) Novel cross-links that result from application of enzymes have also been implemented in mRNA display.

mRNA display libraries can also be directly modified through the introduction of exogenous reagents or biosynthetic enzymes after translation. Covalent mRNA display peptide libraries have been successfully generated either by removing protecting group side chains *in situ* ^421^ or through dicysteine alkylations prior to incubation with the desired protein of interest.^422^ The Bowers group has introduced pyridine-thiazoline,^423^ tyrosyl,^424^ and lactam^425^ cyclization by employing promiscuous thiazoline oxidase, megTYR, and mTG enzymes, respectively (Figure 37C). Suga et al. developed lactazole thiopeptide-mimicking RiPPs that can bind to Traf2-NCK-interacting (TNIK) Kinase with high affinity.^426^ The crystal structure reveal that a macrocyclic hit adopts an antiparallel β-sheet conformation, demonstrating that incorporation of an unnatural backbone can orient hydrogen bond donor and acceptor pairs to form distinct secondary structures. An excellent review of applications on mRNA display for undruggable targets was recently published.^427^

#### 4.2.3 DNA-Encoded Libraries

Phage and mRNA display require ribosomal synthesis and, therefore, are limited to peptide and protein libraries. DNA-encoded libraries (DELs) leverage a powerful integration of purely chemical combinatorial chemistry and next-generation DNA sequencing and informatics to significantly expand the chemical space available for drug discovery. First discussed by Brenner and Lerner in 1992, DELs have gathered significant interest in the past decade.^428,429^ DELs are typically synthesized through split-and-pool method or DNA-templated synthesis with theoretical diversity reaching up to 10^10^ members.^430–432^ Each DNA molecule in the library serves the purpose of an identifier, a bar code, for the attached synthetic small molecule or peptide. After screening, the binders are identified through next-generation DNA sequencing (Figure 38A). In keeping with the theme of this section, we will focus on the use of DELs to develop macrocyclic peptide ligands for protein surfaces. Several approaches to cyclize peptides on DNA have been pursued, including copper-mediated azide–alkyne cycloaddition (click), the Wittig reaction and cysteine alkylation to obtain thioethers (Figure 38B).

One of the first DEL-enabled small molecule clinical candidates was developed from split-and-pool synthesis for RIPK1, a receptor interacting protein 1 kinase that links to inflammatory diseases.^433^ In split-and-pool approach, libraries are built through a series of chemical transformations, each chemical building block is encoded by the addition of a unique DNA fragment. DNA ligation is required after each chemical synthesis and the resulting mixtures are then pooled and split for another round of chemical transformation (Figure 39A). As an example of this powerful platform, the initial hit compound GSK481 was identified by GlaxoSmithKline (GSK) through a DEL screening campaign involving 7 billion compounds, which were screened against immobilized RIPK1.^433^ Subsequent structural optimization led to the discovery of GSK2982772, a compound exhibiting remarkable kinase specificity and an improved pharmacokinetic profile. In a separate study, Silvestri et al. applied the split-and-pool method in their DEL screening to discover UNP-6457, a neutral nonapeptide from a library of 4.5 billion compounds, which demonstrated inhibitory activity toward the MDM2–p53 interaction.^434^ Macrocyclization of UNP-6457 was achieved via copper-catalyzed azide–alkyne cycloaddition between the N-terminal azidoacetamide and C-terminal propargyl glycine. Hit compound exhibited an IC_50_ of 8.9 nM and X-ray structure analysis of MDM2–UNP-6457 interaction revealed deep binding pocket of 3-trifluoromethyl phenylalanine of UNP-6457 interacting with Phe-Trp-Leu motif of MDM2 (Figure 39B and Figure 9A). These studies exemplify the power of DEL technology and lay the groundwork for its future applications in drug discovery.

In addition to split-and-pool platform, DNA-templated synthesis (DTS) (Figure 40A), first reported by the Liu lab in 2001, offers an alternative method for constructing DNA-encoded libraries (DELs).^431,435^ This approach leverages pools of pre-encoded DNA templates to guide the recruitment of chemical building blocks to a reaction site via hybridization, enhancing the effective molarity of substrates linked to complementary oligonucleotides and facilitating chemical transformations in a single solution. In a proof-of-concept study, Gartner et al. applied DTS to create a 65-membered macrocyclic fumaramide library, which was subjected to an *in vitro* selection for binding carbonic anhydrase.^435^ The process involved three consecutive DNA-templated reactions, each encoded by a distinct 12-base DNA region, followed by an efficient aqueous Wittig macrocyclization. This strategy successfully generated macrocyclic fumaramides conjugated to their encoding DNA templates. After two rounds of selection, a single library member with a phenyl sulfonamide group, known to confer carbonic anhydrase activity, was enriched. In another application of DTS in DEL screening, Seigal et al. identified BIR2 and BIR3 domain inhibitors of X-Chromosome-linked inhibitor of apoptosis protein (XIAP) from a focused library of 160,000 compounds.^436^ XIAP, a member of the inhibitor of apoptosis protein family, regulates cell death by inhibiting caspases and other pro-apoptotic signals. Overexpression of XIAP is often linked to tumor progression and poor cancer prognosis. In this study, macrocyclic pentapeptides were synthesized using DTS, cyclized via Click chemistry, and screened against immobilized XIAP. Optimization of an initial hit led to the development of a potent dimeric inhibitor that blocks the BIR2/3-SMAC interaction with an IC50 of 3 nM, demonstrating significant functional activity in a caspase-3 rescue assay (Figure 40B).

DELs have increasingly been used not only for discovering new drug candidates but also for uncovering novel chemical reactions. By systematically screening these libraries, DELs enable the identification of novel chemical transformations, new reaction pathways, and the optimization of catalytic conditions. Recent advancements in DNA-compatible reactions—ranging from photocatalytic coupling reactions to C–C bond formation and macrocyclization—have significantly enriched the DEL reaction toolkit. For an in-depth review of the latest innovations applicable to DELs, we guide the readers to several excellent reviews.^437,438^

#### 4.2.4 Split-Intein Circular Ligation of Peptides and Proteins (SICLOPPS) Libraries

Several powerful platforms have been developed for the generation and screening of cyclic peptide libraries, each offering distinct advantages. While mRNA and DNA display provide the potential to produce macrocyclic peptide libraries with non-natural residues *in vitro*, split-intein circular ligation of peptides and proteins (SICLOPPS) allows genetic encoding of cyclic peptide libraries in cells.^439^ The ability to generate and screen large libraries in live cells sets SICLOPPS apart from the affinity-based *in vitro* screening methods discussed above.

In SICLOPPS, a library of target peptides is flanked by the C- and N-terminal segments of a split intein (I_C_ and I_N_, respectively). The expressed fusion protein folds to form an active intein resulting in an N-to-S acyl shift at the I_N_ junction, generating a thioester intermediate. This intermediate then undergoes transesterification with a nucleophilic side chain—typically a cysteine or serine located at the I_C_ junction, forming a lariat intermediate. Further rearrangement of the lariat intermediate generates the thermodynamically favored lactam product (Figure 41A).

SICLOPPS libraries have been used in combination with a hybrid system for the identification of PPI inhibitors. This approach enables the selection of peptides that disrupt specific PPIs by linking inhibition to a measurable phenotype (Figure 41B). This method has allowed identification of mammalian protein–protein interactions from SICLOPPS libraries.^440–442^ Of note, Tavassoli and co-workers utilized a live–dead screen to select inhibitors of hypoxia inducible factor 1α and 1β (HIF-1α and HIF-1β). The two-hybrid system has demonstrated functionality not only in *E. coli*, but also in yeast and human cells, highlighting its versatility across different organisms.^443^

Recent work by Ball et al. showcased the power of the SICLOPPS high-throughput screening platform to identify a dual inhibitor of HIF1/HIF2 transcription factors by inhibiting the interaction of both HIF-1α and HIF-2α with HIF-1β.^444^ From a genetically encoded library of 3.2 × 10^6^ *cyclo*-CXXXXX cyclic peptides (where X = any amino acid), the 3 lead molecules identified in this screen all contained the same tripeptide pharmacophore, IFC motif, that was shown to bind the HIF-1α and HIF-2α proteins (Figure 41C). Further structure–function relationship analysis led to identification of c*yclo*-CRLII­(4-iodo)­F, a cell-permeable potent cyclic inhibitor of HIF that disrupts hypoxia-response signaling in several cancer cell lines. Together, this study highlights the potential of the SICLOPPS platform for discovering potent inhibitors of protein–protein interactions, underscoring its value in targeting challenging intracellular pathways such as HIF.

One critical advantage of SICLOPPS strategy is that the macrocycles can be screened against a target in the context of the cellular complexity, this means that the libraries can be screened for function and not just binding. This cell-based screening approach allows for the identification of compounds with intracellular efficacy, which is particularly valuable for targeting protein–protein interactions. A notable limitation of the SICLOPPS platform is its current restriction to the 20 canonical amino acids, with only a few studies demonstrating the incorporation of non-natural residues via orthogonal aminoacyl-tRNA synthetase/tRNA pairs.^443^

**Figure 38:**
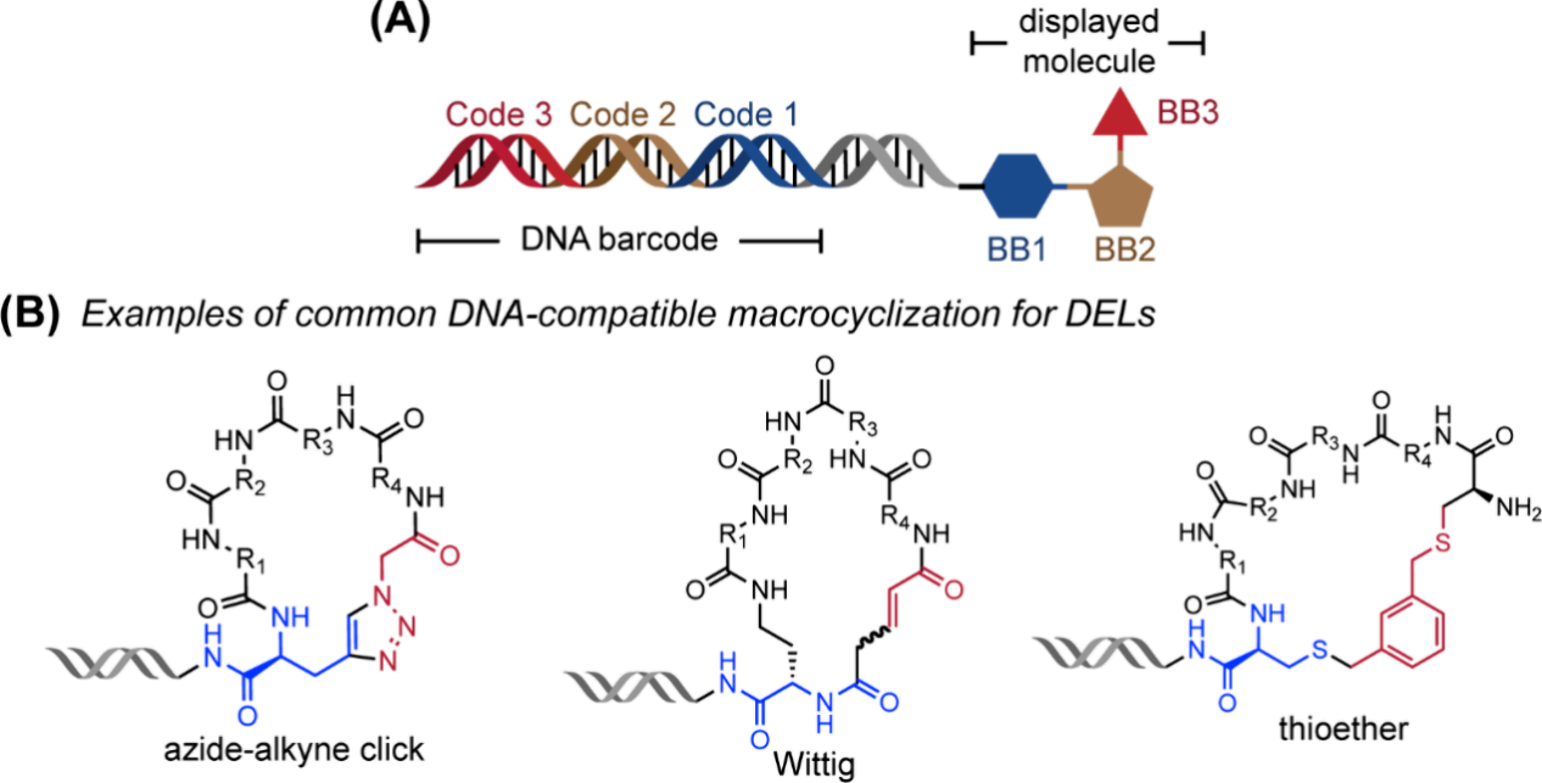
(A) Schematic representation of a DNA-encoded chemical library. DNA fragments encode the building blocks 1–3 (BB 1–3), which are incorporated in the final compound. (B) Common DNA-compatible macrocyclization chemistries used in DELs.

**Figure 39:**
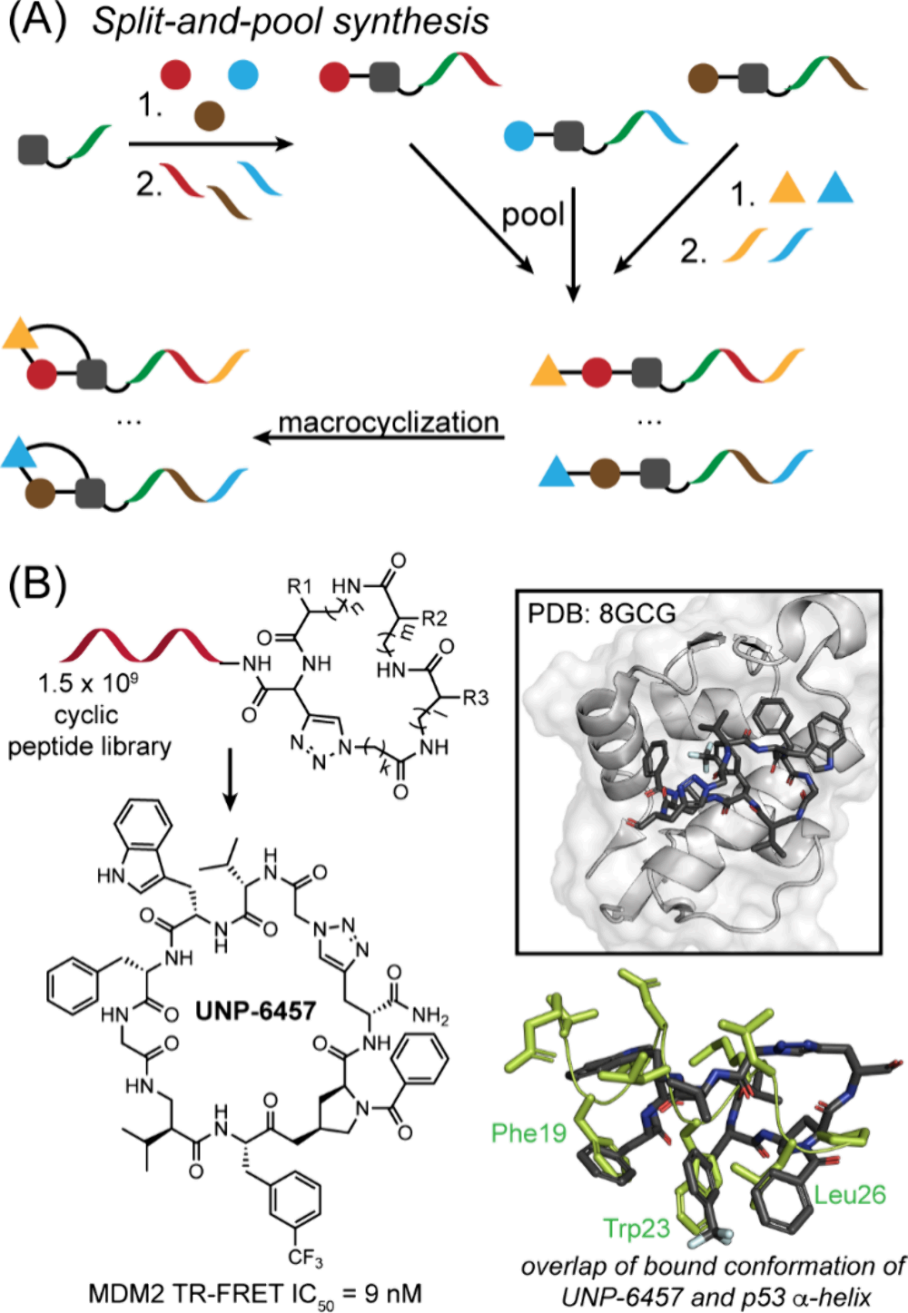
(A) Macrocyclic library prepared by split-and-pool method where each chemical building block “1” is encoded by the addition of a unique DNA fragment “2”. (B) DNA-encoded macrocyclic peptide libraries enable the discovery of a macrocyclic peptide, UNP-6457, that inhibits MDM2–p53 interaction.

**Figure 40:**
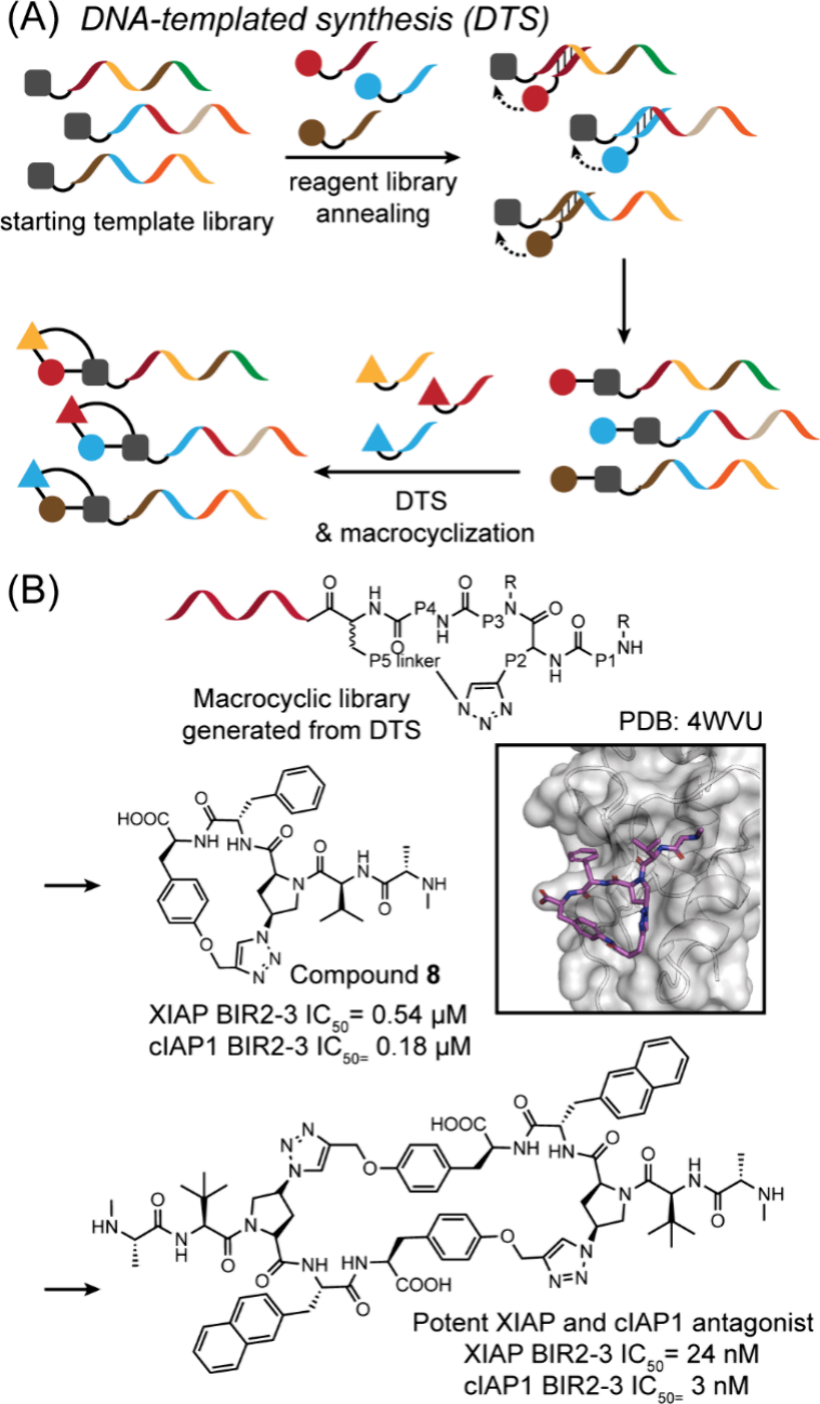
(A) Macrocyclic library prepared by DNA-templated synthesis (DTS) where pre-encoded DNA templates guide the recruitment of chemical building blocks to a reaction site via hybridization. (B) Macrocyclic inhibitors that displace bound pro-apoptotic caspases. Optimization of hit compounds led to a dimeric macrocycle that inhibits BIR2/3-SMAC interaction.

## 5 Beyond Inhibition of Protein–Protein Interactions

In the preceding sections, we discussed various therapeutic modalities designed to engage intracellular proteins. Here, we highlight a pivotal aspect of PPI modulators, their potential to evolve into proximity-inducing bifunctional modalities capable of probing and reprogramming cellular processes.

### 5.1 Repurposing Known PPI Inhibitors for Induced Proximity-Based Degradation

A known PPI modulator can be repurposed into a heterobifunctional molecule to induce proximity between two target protein. The emerging field of targeted protein degradation (TPD) has rapidly gained momentum as a powerful therapeutic strategy in drug discovery, attracting considerable attention in both pharmaceutical industry and academic laboratories for its potential to address previously intractable targets.^445^

TPD harnesses the cell’s natural ubiquitin-proteasome system (UPS) to eliminate disease-causing proteins by recruiting them to E3 ubiquitin ligases, leading to their ubiquitination and subsequent degradation by the proteasome. Such degraders are often further categorized into bifunctional proteolysis-targeting chimeras (PROTACs) or molecular glue degraders (Figure 42A). Compared to classic small molecule inhibitors, these degraders offer several key advantages: (1) classical PPI inhibitors may bind to the orthosteric or allosteric sites to modulate protein activity but such considerations do not need to be taken into account if the protein is degraded; (2) unlike occupancy-driven inhibitors, PROTACs and molecular glue degraders act catalytically, enabling one degrader molecule to eliminate many copies of the target protein; (3) target degraders can engage and degrade proteins with resistance mutations and/or upregulation since degraders are not required to bind to a specific orthosteric or allosteric regulation site of the target. As our understanding of TPD deepens, the degrader toolbox continues to expand, offering accessibility to many undruggable proteins and paving the way for new therapeutic directions. Since the earliest PROTAC report,^445^ over 6,000 heterobifunctional molecules that engage over 100 E3 ligands and act on more than 400 proteins have been described.^446^ Among the greater than 600 E3 ligases in the human genome, cereblon (CRBN) and von Hippel-Lindau (VHL) are the best characterized and commonly engaged substrate receptors for TPD applications.

Macrocycles and protein domain mimics, as discussed in Section 3, are uniquely suited to modulate challenging proteins with large and flat surfaces, interactions often inaccessible to conventional small molecules. One compelling example is cyclosporin A, a macrocyclic natural product that was used to prevent transplant rejection since 1983. Its mechanism of action was not elucidated until a decade later when it was discovered to function as the a peptide-based molecular glue promoting ternary complex formation between calcineurin and cyclophilin.^447^ This discovery not only demonstrated the therapeutic potential of peptides but also laid the foundation for leveraging peptide-based scaffolds as protein–protein interaction stabilizers, enabling access to protein surfaces beyond the reach of small molecule degraders.

The discovery of cyclosporin A as a PPI stabilizers has inspired design of folded peptides as bispecific PROTACs that engage the target protein on one face of the folded peptide and an E3 ligase recognition sequence on the other, enabling selective and programmable degradation in one construct (Figure 42B).^449^ Numerous peptidic degron motifs, short peptide sequences that act as degradation signals, have been identified for over 20 E3 ligases, many of which remain untapped for PROTAC development.^451,452^ Coupled with advances in display technologies for rapid hit identification, these insights pave the way for a new generation of peptidic molecular glue degraders with enhanced potency and target specificity.

A recent study by FOG Pharmaceuticals (now, Parabilis Medicines) identified stapled peptides, termed Helicons, that function as degraders by promoting cooperative interactions between E3 ligase CHIP and TEAD4, as well as MDM2 and β-catenin.^450^ Through two successive rounds of phage display screening, the authors identified α-helical peptides with molecular glue-like properties. Notably, Helicon H330 was shown to facilitate β-catenin binding only in the presence of MDM2. Structural analyses revealed that H330 simultaneously occupied the p53-binding site on MDM2 and the ICAT-binding site on β-catenin (Figure 42B). Although target degradation was not demonstrated in cellular assays, this work highlights the potential of display technologies and macrocyclic peptide scaffolds in the design of peptide-based molecular glue degraders.

While peptide-based molecular glues rely on accessible surface contacts for ternary complex formation, macrocyclic peptides and peptidomimetics lacking such interfaces can also be converted into PROTACs by conjugating them with established E3 ligase ligands, such as those for CRBN, VHL, or other ligases described above. A recent study from Hiroaki Suga’s group demonstrated this approach by identifying a macrocyclic peptide closed via a thioether–bipyridyl unit and linked to a proteasome-targeting motif (RRRG), which resulted in modest yet detectable degradation of BRD4 (Figure 42B).^448^

#### 5.1.1 Controlling Protein Ubiquitination Levels with Deubiquitinases

Many diseases are linked to the destabilization and degradation of proteins. In nature, deubiquitinases (DUBs) play a key role in reversing this process by removing ubiquitin from tagged proteins, thereby rescuing them from proteasomal degradation. Inspired by this mechanism, Nomura and co-workers have developed bifunctional molecules called deubiquitinase-targeting chimeras (DUBTACs), which leverage the concept of induced proximity to recruit DUB into proximity with a specific protein. This targeted recruitment restores protein stability and prevents degradation (Figure 43A). The emerging field of targeted protein stabilization (TPS) builds on this strategy, offering a novel therapeutic approach to control protein ubiquitination levels. In a proof-of-concept study, Henning et al. reported a covalent DUBTAC, EN523, that is capable of targeting noncatalytic allosteric C23 in the K48-ubiquitin-specific deubiquitinase OTUB1 and recruiting OTUB1 to CFTR, a mutated and misfolded protein in cystic fibrosis.^453^ As a result, treatment of EN523 led to stabilized ΔF508-CFTR protein levels and improved chloride channel conductance in human cystic fibrosis bronchial epithelial cells. For a more comprehensive view on recent advances and therapeutic benefits of TPS, we direct our readers to an excellent review on DUBTACs.^454^

### 5.2 Proximity-Inducing Bifunctional Molecules beyond Ubiquitination

In addition to the targeted protein degraders discussed above, a growing number of heterobifunctional scaffolds have been developed to induce proximity between proteins for functions beyond degradation.^455^ While bifunctional molecules such as PROTACs and molecular glue degraders have achieved significant success and quickly established themselves as key therapeutic strategies for targeted protein degradation, other classes of targeting chimeras, such as LYTACs (lysosome-targeting chimeras),^456^ AUTACs (autophagy-targeting chimeras),^457^ and RIBOTACs (ribonuclease-targeting chimeras),^11^ have recently been developed to expand the degradable substrate landscape to include membrane-bound, extracellular proteins, and RNAs (Figure 43A).

**Figure 41:**
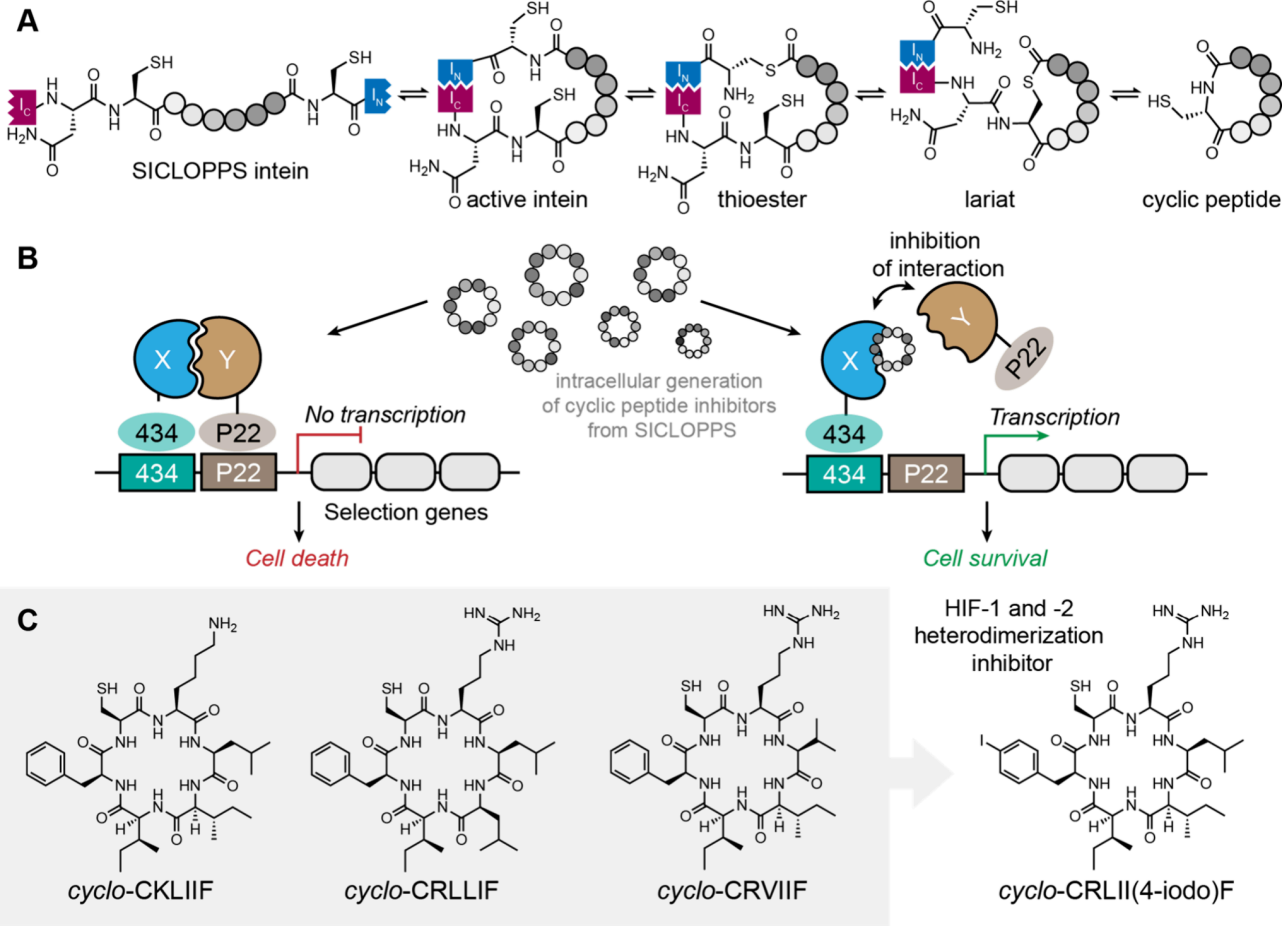
SICLOPPS selection of cyclic peptides. (A) Mechanism of cyclic peptides produced by SICLOPPS. Figure adapted from Sohrabi et al.^386^ (B) The bacterial reverse two-hybrid system is often used in conjunction with SICLOPPS *in cellulo* to select for cyclic peptide inhibitors of protein–protein interactions. (C) A recent report from Ball et al. identified a series of cyclic peptides from SICLOPPS (left) that inhibit the interactions of both HIF1α and HIF2α with HIF1β. These compounds are further optimized to yield *cyclo*-CRLII­(4-iodo)­F (right).

**Figure 42:**
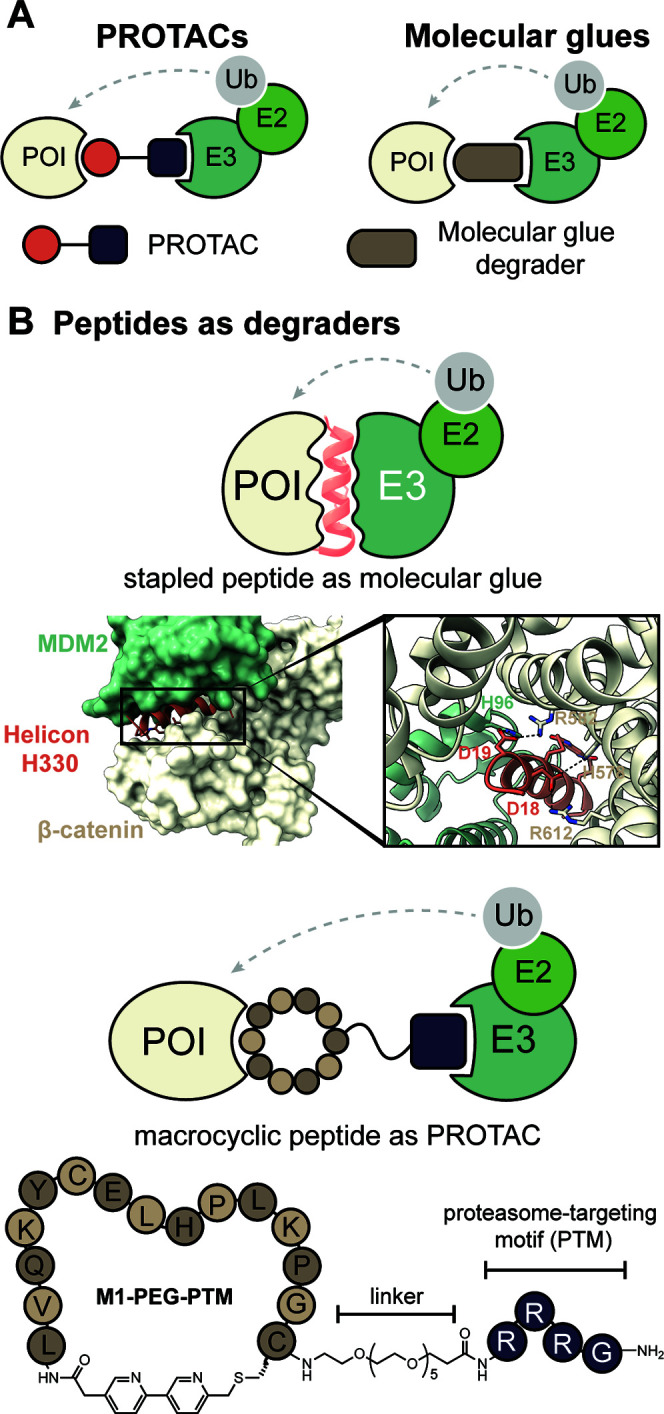
(A) PROTACs and molecular glues induce protein–protein interactions with an E3 ligase to induce ubiquitination and proteosomal degradation of a target protein. (B) Peptide-based degraders in targeted degradation landscape. (Top) Macrocyclic peptides and peptidomimetics lacking available contact surfaces can be transformed into PROTAC by linking to an established E3 ligase ligand. Macrocyclic peptide M1-PEG-PTM cyclized by a thioether–bipyridyl unit is tagged with a proteasome target peptide motif (RRRG), resulting in degradation of BRD4.^448^ Figure adapted from Jing et al.^449^ (Bottom) Helicon H330 identified from phage screening libraries promotes ternary complex formation between MDM2 and β-catenin.^450^

**Figure 43:**
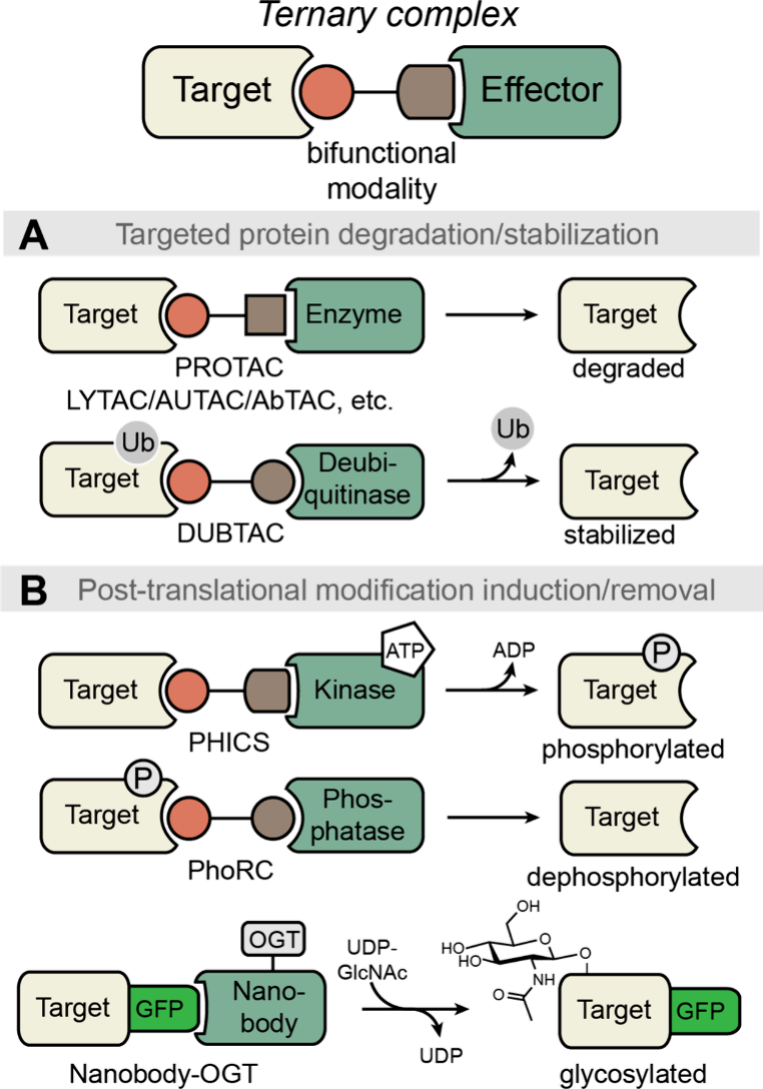
Schematic representation of heterobifunctional molecules.

#### 5.2.1 Bifunctional Modalities for Induction and Removal of Post-Translational Modifications (PTMs)

Besides degradation-inducing bifunctional molecules, nondegradation bifunctional modalities have been extensively explored to induce or remove PTMs such as phosphorylation, glycosylation, and acetylation on any protein of interest.^455^ Protein phosphorylation is the most prevalent and extensively studied PTM in cells, playing a central role in regulating signaling pathways. Artificially inducing phosphorylation on specific proteins can therefore modulate downstream signaling cascades. An early example from the Schepartz group demonstrated this concept by exploiting a bifunctional fusion protein that brought together two miniature proteins, YY2 and 3.3, each designed with selective binding affinities for their respective targets. This adaptor redirected the Src family kinase Hck to phosphorylate hDM2—a nonsubstrate protein to Hck, a process that Hobert et al. termed “templated catalysis.”^458^ The resulting ternary complex among hDM2, Hck, and the adaptor effectively rewired a cellular signaling event, leading to p53 reactivation and the upregulation of p53-dependent genes (Figure 43B).

In another approach introduced by Amit Choudhary’s research group in 2020 that bypasses the need for engineered fusion proteins, bifunctional compounds, termed phosphorylation-inducing small molecules (PHICS), have been shown to recruit kinases both AMP-activated protein kinase (AMPK) and protein kinase C (PKC) to a non-native substrate, enabling both native and neo-phosphorylation of BRD4.^459^ Similar to PROTACs, PHICS use a modular design, one end binds the kinase, and the other binds the substrate, allowing for precise control over phosphorylation events (Figure 43B). More recently, next-generation PHICS have been developed to recruit not only serine/threonine kinases but also tyrosine kinases.^460,461^ These phosphorylations triggered by PHICS have been shown to have inhibitory effects on the activity of Bruton’s tyrosine kinase (BTK) and BCR-ABL-dependent cancer cells. Overall, we envision that PHICS-mediated phosphorylation will increasingly be integrated into peptide-based therapeutic strategies in the near future.

In addition to phosphorylation, dephosphorylation modalities were also developed to remove unwanted PTM on a target protein of interest. In a proof-of-concept study by Yamazoe et al., heterobifunctional molecules, named phosphatase-recruiting chimeras (PhoRCs), were designed to provide proximity between POI and phosphatase, promoting POI dephosphorylation.^462^ In this study, synthesized PhoRCs recruited both native and fusion proteins using a target-binding ligand (AKT or EGFR inhibitor) and a HaloTag-reactive chloroalkyl or protein phosphatase 1 (PP1)-activating peptide (RVSF) as an effector-binding motif, induced the PP1-dependent dephosphorylation of AKT and EGFR.^462^

Bifunctional molecules, which include peptide macrocycles, designed to induce or remove PTMs such as glycosylation and acetylation have also been developed, with several recent reviews providing comprehensive overviews of these advances.^463–465^ Moving forward, we anticipate that the inherent versatility of peptide scaffolds will play a key role in broadening the scope of proximity-inducing modalities beyond traditional degrader platforms.^455^

## 6 Significant Challenges: Synthesis and Cellular Uptake of Peptidic Molecules

In this review, we highlighted the promise of large molecules and constrained peptides to modulate PPIs. However, these complex structures enhance their synthetic difficulty because precise control is required over sequence assembly and macrocyclization. Examination of recent clinical-stage macrocycles and engineered proteins reveals common bottlenecks that chemists have tackled to make these architectures accessible at preparative scale. Despite their potential, peptides, including macrocycles, often suffer from poor oral bioavailability and limited cell permeability. Ongoing efforts aim to overcome these challenges, and strategies for enhancing cellular uptake of such macromolecules are discussed below.

### 6.1 Synthetic Challenges in Development of Complex Peptides and Macrocycles PPI Inhibitors

Peptides spanning 50 to 100 residues exceed practical synthetic limits imposed by solid-phase peptide synthesis (SPPS). The stepwise linear synthesis of such lengthy peptides often suffers from poor coupling efficiencies, increased impurity accumulation, sequence-dependent aggregation, and incomplete resin cleavage, collectively diminishing yields and complicating purification. Consequently, purely linear SPPS becomes an inadequate approach for reliably accessing high-purity mini-protein sequences.^466^

To circumvent these limitations, modular ligation-based strategies, including the native chemical ligation (NCL),^467^ ketoacid–hydroxylamine ligation (KAHA),^468^ and others,^469,470^ have been developed for assembling larger peptide sequences. Enzyme-mediated ligations, such as sortase-catalyzed methods have provided regioselective and mild reaction conditions suitable for cyclizing and assembling sensitive mini-protein scaffolds, further expanding synthetic accessibility.^471^ Omomyc offers an exemplary case of a long peptide or miniprotein being developed as an inhibitor of the transcription factor MYC. Brown et al. leveraged the chemoselectivity and robustness of NCL to efficiently connect discrete peptide segments, thereby significantly improving synthetic yields and maintaining functional integrity.^472^

Complementing these ligation strategies, recent advancements in automated solution-phase flow synthesis provide innovative routes to overcome inherent SPPS limitations. Flow-based approaches offers enhanced control over coupling reactions, reduces aggregation through rapid and continuous reaction conditions, and achieves high yields even for long sequences typically challenging for conventional SPPS.^473^ By precisely controlling reaction parameters in a continuous-flow manner, this technology has demonstrated remarkable scalability and efficiency in assembling lengthy peptide and mini-protein sequences previously considered synthetically intractable by traditional resin-bound methods. However, the excessive usage of reagents required to for efficient couplings would need to be addressed for wide-scale adoption of this approach.

Macrocycles represent highly attractive scaffolds for protein–protein interaction (PPI) inhibition due to their unique ability to adopt rigidified conformations inaccessible to conventional small molecules, enabling high-affinity interactions with challenging biological targets.^322^ Despite their therapeutic promise, synthesizing macrocyclic peptides often presents considerable synthetic hurdles. These challenges include complexities arising during macrocyclization steps, such as decreased reaction efficiency with increased ring size and the potential formation of undesired oligomeric or polymeric side products.^474^ Furthermore, modifications like N-methylation, often crucial for enhancing cell permeability and bioavailability, can complicate the cleavage from solid-phase resin, necessitating careful optimization of conditions to avoid side reactions or cleavage inefficiencies.

Recent progress illustrates the field’s growing ability to surmount these synthetic obstacles. For instance, Chugai’s orally bioavailable KRAS inhibitor, LUNA18, leveraged rigorous backbone N-methylation to achieve membrane permeability, carefully tuning of resin cleavage conditions was needed to obtain high yields from solid phase synthesis (Figure 7). Chugai scientists encountered additional complexity, as extensive backbone modifications aimed at improving physicochemical properties (e.g., lipophilicity and permeability) significantly complicated synthetic steps, demanding meticulous condition optimization to balance bioactivity and conformational rigidity simultaneously.^53^

Similarly, access to Merck’s PCSK9 inhibitor (MK-0616, enlicitide decanoate)^326,327^ required multiple cyclizations; each cyclization reaction needs to be designed to overcome competitive oligomerization (Figure 44A). Through strategic route refinement, fragment-based assembly, and precise cyclization condition optimization, Merck developed a scalable and efficient synthetic route, successfully overcoming the macrocyclization bottlenecks. However, the synthesis required 43 overall steps highlighting the challenges in total synthesis of macrocyclic peptide therapeutics.^475^

**Figure 44:**
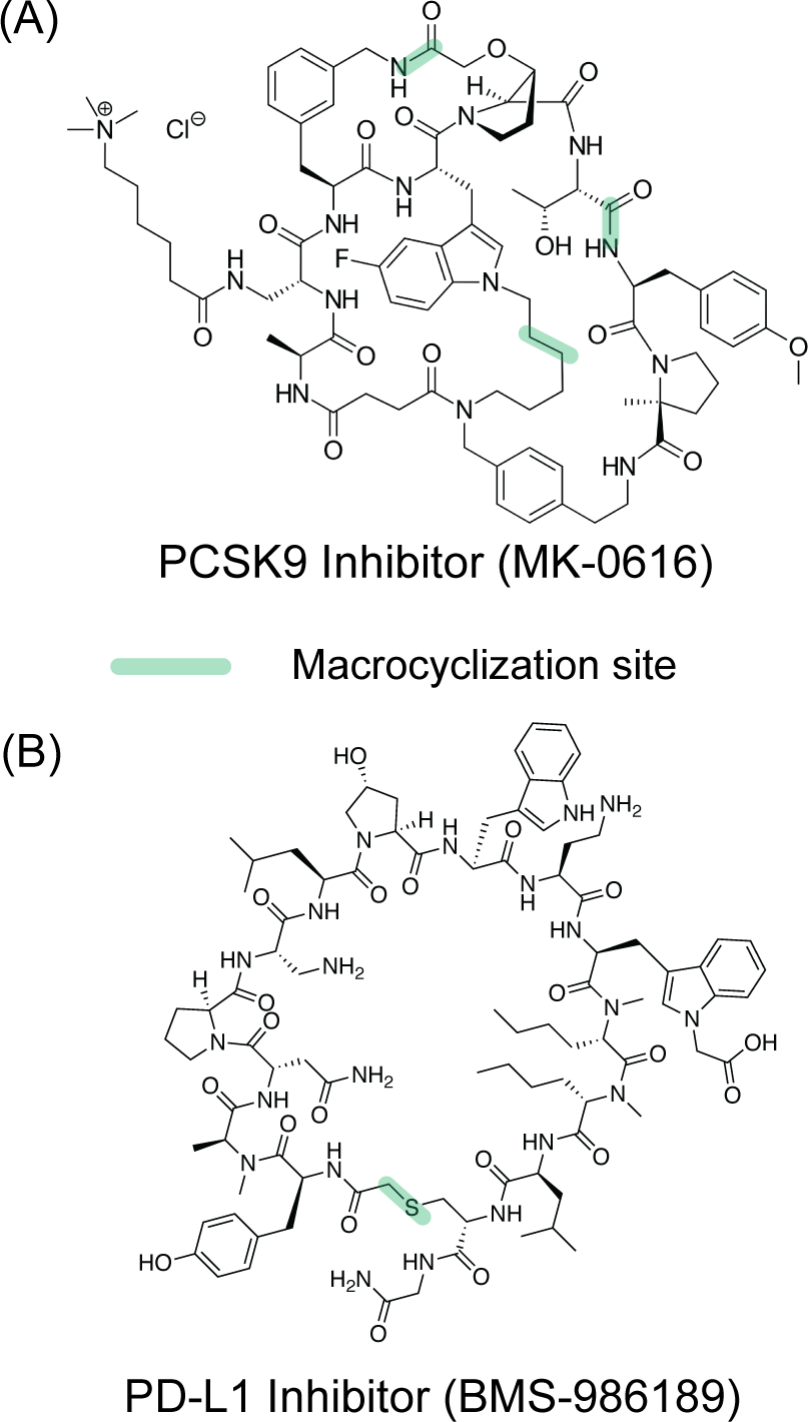
Macrocyclic peptides that have been synthesized at process scale.

Similarly, BMS’s PD-L1-targeted macrocyclic peptide, BMS-986189, faced substantial synthesis challenges due to extensive N-methylation. Multiple N-methylated unnatural amino acids led to increased susceptibility to hydrolysis during global side-chain deprotection, significantly impacting yield and purity.^476^ The acid-mediated formation of oxazolone intermediates near these N-methyl residues was identified as a critical mechanistic problem, prompting BMS researchers to meticulously optimize reaction conditions—such as modifying scavenger compositions in the cleavage cocktail—to significantly mitigate side reactions and impurity formation. Additionally, the synthesis faced escalating complexity upon scale-up, underscoring the necessity of stringent impurity control and robust process optimization. Macrocyclization efficiency markedly decreased with larger ring sizes, requiring extensive refinement of reaction parameters (e.g., dilution levels, reagent stoichiometries, and reaction timing) to minimize undesired polymeric products and ensure successful ring closure (Figure 43B).

Collectively, these recent examples underscore the synthetic challenge to access peptide macrocycles, but also highlight the advancements in synthetic methodologies to successfully addressing these challenges.

### 6.2 Challenges and Progress in Cellular Uptake of Large Molecules

This review outlines methods to optimize engagement of a protein target, however intracellular targeting of proteins also requires the ligand to have sufficient cellular uptake. The cell membrane is a semipermeable lipid bilayer that passively and actively mediates transport in and out of cells. Small molecules can often passively diffuse across the cell membrane.^477^ Efforts are underway to understand rules for passive uptake of peptides and peptide-based macrocycles. Common approaches to enhance peptide and macrocycle cellular uptake include pro-drug strategies reducing overall polarity like esterification, amide bond surrogates reducing hydrogen bonding groups, and macrocyclization sequestering polar and hydrogen bonding groups.^478–483^ Natural macrocycles, such as cyclosporine A (CsA), serve as the model for these efforts.^484–486^ N^α^-Methylation of amino acid residues to remove the hydrogen bond donors has led to macrocycles with enhanced permeability.^323,487^

Large biological molecules such as proteins, peptides, and nucleic acids have tremendous potential as therapeutics and research tools, but delivering these macromolecules into the cell interior is notoriously challenging. The discovery that the HIV-1 Tat peptide can translocate across cell membranes provided a breakthrough.^488^ This finding led to the concept of cell-penetrating peptides (CPPs)—typically short (10–30 amino acid) cationic or amphipathic peptides capable of traversing membranes at micromolar concentrations.^489^ Over the years, many CPPs have been identified and used to deliver diverse cargoes (small molecules, proteins, nucleic acids, etc.) into cells. However, despite their promise, most CPP-cargo complexes remain trapped inside endosomal vesicles, resulting in poor release to the cytosol. Endosomal sequestration and inefficient escape represent a fundamental bottleneck that has limited the success of CPPs and other delivery vectors.^490^

To overcome these challenges, researchers have explored a variety of strategies for improving the cytosolic delivery of large biomolecules. In this discussion, we highlight five noteworthy approaches (Figure 45): (A) the development of cyclic CPPs that trigger a vesicle budding and collapse escape mechanism; (B) the use of cell-surface-anchoring to promote cytosolic entry; (C) the re-engineering of cell-permeant miniature proteins that exploit the endosomal homotypic fusion and protein sorting complex to facilitate escape; (D) leveraging of reversible covalent chemistry to cloak peptides and proteins to enhance uptake and (E) the exploitation of macropinocytosis as a route for internalizing extracellular proteins. Each of these strategies provides unique insights into how large biomolecules can bypass or overcome cellular membranes.

**Figure 45:**
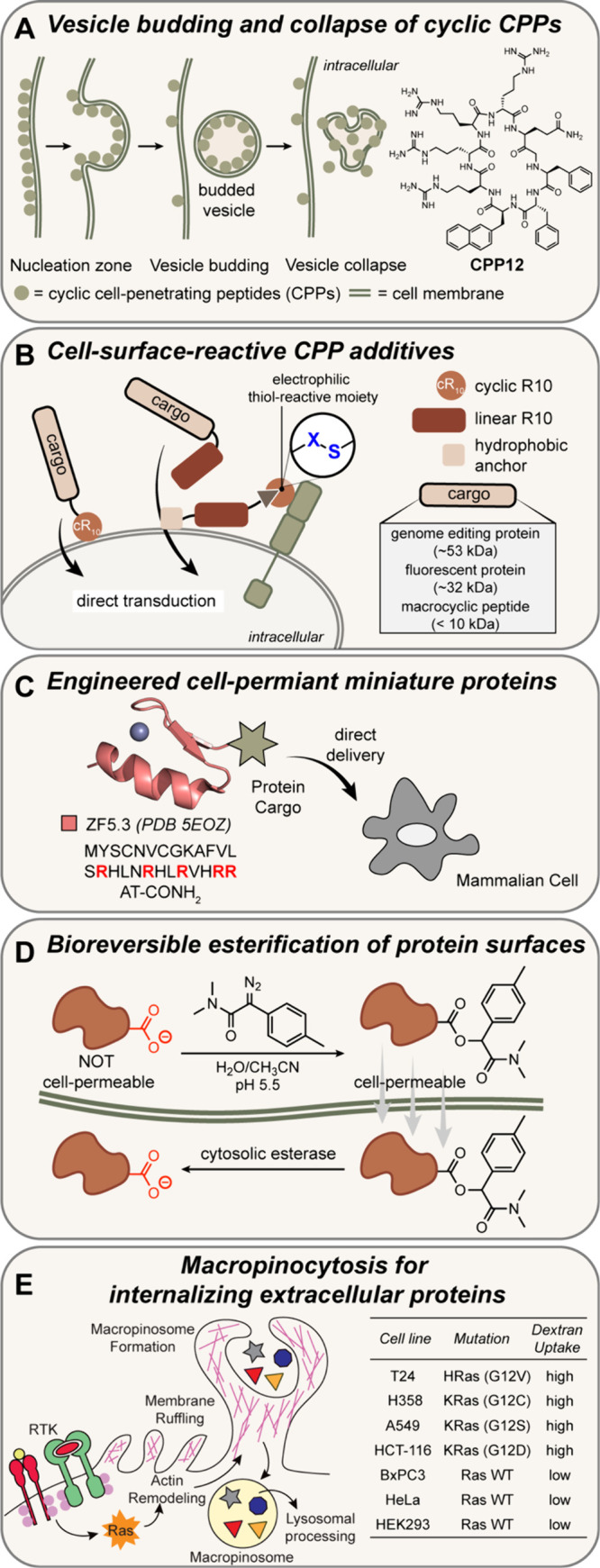
Schematic illustration of rapidly developing approaches to enhance uptake of peptides and small proteins: (A) cyclic CPPs have been designed to leverage vesicle budding mechanisms; (B) covalent targeting of cell-surface allows anchoring of cargo to promote cytosolic entry; (C) miniproteins have been reengineered to exploit endosomal homotypic fusion and protein sorting complexes; (D) reversible covalent chemistry has been leveraged to to cloak protein carboxylic acids as esters to reduce protein surface negative charge; and (E) macropinocytosis, and other active transport mechanisms, have been leveraged as a cell-specific route for internalizing peptides.

Dehua Pei and co-workers discovered that certain small amphipathic cyclic peptides can achieve remarkably high cytosolic delivery efficiencies. These cyclic CPPs not only enter cells readily but also escape endosomal compartments far more effectively than linear CPPs.^491^ Unlike classic CPPs (i.e., a linear Tat peptide) that often get trapped in endosomes, some of the optimized cyclic peptides achieved improved delivery. These cyclic CPPs, typically 9–12 residues in length, bind strongly to plasma membrane phospholipids, enter cells via endocytosis, and they escape endosomes. By tracking their behavior in vesicles and live cells, Pei and co-workers described this phenomenon as vesicle budding. These vesicles then collapse—presumably rupturing or fusing back—to release their contents into the cytosol. Microscopy provided visual evidence of CPP-rich lipid buds forming and breaking apart, consistent with this escape route (Figure 45A).^492,493^

Covalent conjugation of the cargo to the cell surface may initiate internalization; this concept has been leveraged to develop a suite of elegant chemistries. The Raines group showed that modified boronate esters that can engage diols of cell surface saccharides to internalize proteins.^494^ Hackenberger and co-workers demonstrated that large biomolecules can be delivered intracellularly by designing cell-penetrating peptide additives that covalently anchor to the plasma membrane. They showed that an arginine-rich peptide equipped with a thiol-reactive electrophile (e.g., a maleimide) will attach to cell-surface membrane’s thiols and dramatically enhance the uptake of protein cargo–CPP conjugates via a nonendocytic mechanism.^347^ The “surface-anchoring” strategy requires minimal modification of the cargo (Figure 45B).

Moving beyond peptides, Alanna Schepartz and co-workers introduced a new class of cell-penetrating molecules: cell-permeant miniature proteins (CPMPs). These are small-engineered proteins (30–40 residues) that are folded into defined structures and contain strategically placed cationic motifs to enable cell entry.^82,495^ One such molecule, ZF5.3, is a 27-amino-acid mini-protein derived from a zinc-finger domain.^348^ Remarkably, ZF5.3 can deliver a variety of payloads into cells and *in vivo* (Figure 45C).^496^

Raines and colleague have developed methods for direct esterification of carboxyl groups on protein surfaces to reducing negative charge and increasing hydrophobicity.^346^ Such modifications have been shown to facilitate the protein’s passage through the cell membrane. Once inside the cytosol, endogenous esterases cleave these ester groups, restoring the protein to its native, functional form. This approach is based on prodrug strategies to enhance uptake of small molecules but requires specialized esterification strategies to mask and unmask proteins without causing unfolding and denaturation. In an elegant study, the group showed that functional enzymes may be delivered inside cells (Figure 45D).^497^

The above strategies involve designed molecules to enhance uptake, but some cells have naturally enhanced potential to engulf large biomolecules making them suitable for model and therapeutic studies. Work by Bar-Sagi and colleagues has shown that cancer cells driven by oncogenic Ras make upregulate macropinocytosis—an endocytic process of nonselectively engulfing extracellular fluid into large vesicles—to acquire nutrients from their environment (Figure 46).^498^ In a classic 1986 experiment, microinjection of Ras protein into quiescent fibroblasts was shown to increase membrane ruffling and fluid-phase pinocytosis.^499^ From a drug delivery standpoint, this finding suggests that macropinocytosis might be co-opted or stimulated to deliver therapeutic macromolecules to cells.^498^ Significantly, blocking macropinocytosis starved the Ras mutant tumors and stunted their growth *in vivo*. Proteins required for macropinocytosis include Ras, Rac1, and Cdc42, which play an important role during the early phases of macropinocytosis. Additionally, PI3-kinase (PI3K) activation and activity are critical for macropinosome closure and maturation within tumor cells.^500–502^ Several cancerous cell lines with enhanced nutrient uptake demonstrated increased uptake of peptido- and proteomimetics providing potential opportunity for specific targeting of cancer cells (Figure 45E).^500,503^

**Figure 46:**
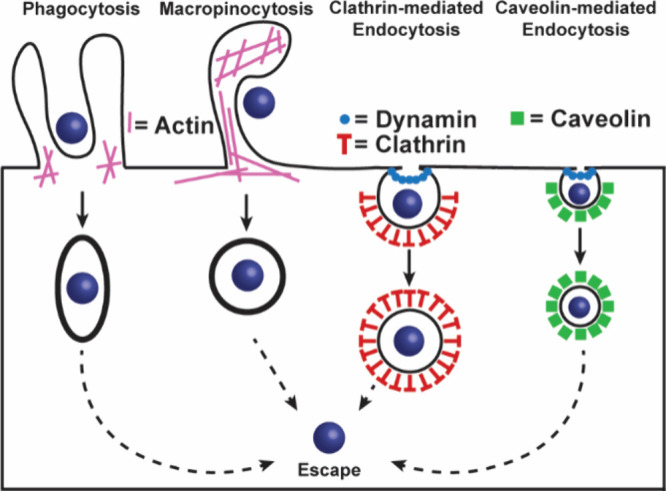
General schematic of endocytotic uptake mechanisms.

Together, these five perspectives underscore the multifaceted approaches being advanced to address the significant challenge of intracellular delivery of peptides and proteins. Carefully designed peptides and miniature proteins can be engineered to either actively disrupt endosomes or to engage host machinery for efficient escape, achieving unprecedented delivery efficiencies. Clever chemical strategies such as esterification and cell-surface-anchored CPPs can physically reroute uptake. Understanding cellular uptake pathways such as macropinocytosis opens the door to exploiting natural routes for therapeutic delivery. Ongoing research is building on these principles to further improve the cytosolic access of biologics.

## 7 Conclusions: Future Directions in PPI Targeting

The field of protein–protein interaction inhibitor design has emerged as a transformative frontier in drug discovery and chemical biology. In this review, we sought to highlight the key advances in the field that have guided success over the past two decades. Technological breakthroughs in computational analysis and screening have reshaped the perception of PPIs as undruggable to being critical for therapeutic innovation. The approval of PPI inhibitors, such as venetoclax (for BCL2 targeting) and Sotorasib (for Ras inhibition), has demonstrated the clinical feasibility of this approach. The development of novel chemical scaffolds, including constrained peptides, macrocycles, and proteomimetics optimized for PPI surfaces, coupled with the convergence of technologies such as fragment-based drug discovery, phage display, mRNA display and DNA-encoded libraries, has refined the identification and optimization of PPI inhibitors.

Advances in artificial intelligence and machine learning are already critical technologies for targeting PPIs. These technologies will accelerate the identification of binding sites, predict interaction hotspots, optimize lead compounds, and analyze vast data sets to uncover trends that guide discovery. The merging of AI with screening strategies is likely to have a revolutionary impact on both methods. At its core, the challenge in PPI inhibition is the challenge in recognizing protein surfaces that lack hydrophobic pockets. Creation of ligands for these surfaces will aid emerging classes of modalities such as proteolysis-targeting chimeras (PROTACs) and molecular glues.^504^ A key disadvantage of high-throughput screening is that, often, the ligand may not bind the orthosteric interface and inhibit protein complexation. Conversion of such ligands into PROTACs will delete the protein from the cell, thereby inhibiting the PPI. We expect that the strategies and methods for developing PPI inhibitors discussed herein will lead to new classes of PROTACs.

The next frontier in the field lies in addressing the critical challenge of targeting dynamic and disordered proteins. Many PPIs involve transient or conformationally dynamic interactions that remain difficult to target with existing approaches. Evidence reveals that many proteins lack a fixed three-dimensional shape, i.e., are “intrinsically disordered”. These proteins acquire their folded structure upon binding a partner protein or ligand. Dysregulated PPIs where one partner is disordered have been implicated in various diseases, including cancer (e.g., p53, MYC), neurodegenerative disorders (e.g., α-synuclein, tau), etc. A systematic approach for targeting such interactions is not yet available.
